# Activation of peroxisome proliferator-activated receptor alpha in human peripheral blood mononuclear cells reveals an individual gene expression profile response

**DOI:** 10.1186/1471-2164-9-262

**Published:** 2008-06-02

**Authors:** Mark Bouwens, Lydia A Afman, Michael Müller

**Affiliations:** 1 Nutrition, Metabolism and Genomics Group, Division of Human Nutrition, Wageningen University, Bomenweg 2, 6703 HD Wageningen, The Netherlands; 2 Dutch Nutrigenomics Consortium, TI Food and Nutrition, Wageningen, The Netherlands

## Abstract

**Background:**

Peripheral blood mononuclear cells (PBMCs) are relatively easily obtainable cells in humans. Gene expression profiles of PBMCs have been shown to reflect the pathological and physiological state of a person. Recently, we showed that the nuclear receptor peroxisome proliferator-activated receptor alpha (PPARα) has a functional role in human PBMCs during fasting. However, the extent of the role of PPARα in human PBMCs remains unclear. In this study, we therefore performed gene expression profiling of PBMCs incubated with the specific PPARα ligand WY14,643.

**Results:**

Incubation of PBMCs with WY14,643 for 12 hours resulted in a differential expression of 1,373 of the 13,080 genes expressed in the PBMCs. Gene expression profiles showed a clear individual response to PPARα activation between six healthy human blood donors. Pathway analysis showed that genes in fatty acid metabolism, primarily in β-oxidation were up-regulated upon activation of PPARα with WY14,643, and genes in several amino acid metabolism pathways were down-regulated.

**Conclusion:**

This study shows that PPARα in human PBMCs regulates fatty acid and amino acid metabolism. In addition, PBMC gene expression profiles show individual responses to WY14,643 activation. We showed that PBMCs are a suitable model to study changes in PPARα activation in healthy humans.

## Background

The function of the nuclear receptor peroxisome proliferator-activated receptor alpha (PPARα) has been studied extensively from the time of its discovery in the early 1990s [1]. PPARα is a ligand activated nuclear receptor, which is known to be activated by free fatty acids and their derivatives [2,3]. Besides fatty acids, several synthetic compounds are available that specifically activate PPARα, including hypolipidemic drugs, such as fibrates and pirinixic acid (WY14,643) [4]. Synthetic PPARα agonists mimic effects of dietary unsaturated fatty acids on hepatic gene expression in terms of regulation of target genes and molecular mechanism [5]. Activation of PPARα with WY14,643 in mice showed that the main function of PPARα in liver is the regulation of lipid metabolism, and more specifically fatty acid β-oxidation [6]. PPARα was also found to be involved in regulation of amino acid metabolism [7] and inflammation [8,9]. In humans, the function of PPARα is examined less thoroughly, because functional studies are more complicated. There is no human genetic disease linked to a dysfunctional *PPARα *gene and tissue sampling is often not feasible in healthy volunteers. Blood is one of the few tissues which is readily available in healthy humans. Peripheral blood mononuclear cells (PBMCs) are relatively easily obtainable by isolation from blood. These cells consist of lymphocytes and monocytes/macrophages and it is known that PPARα is expressed in these cells [10,11]. The use of PBMCs has proven to be highly robust in distinguishing a disease state from healthy state, by studying gene expression profiles of these cells [12,13]. Recently, we showed that PBMC gene expression profiles of healthy volunteers can also reflect changes between 24 and 48 hours fasting, when plasma fatty acid concentrations are elevated. In addition, we showed that PPARα seems to have a functional role in human PBMC during fasting as several of the genes changed upon fasting were also changed upon incubation of PBMC with the specific PPARα agonist WY14,643 [14]. However, the extent of the role of PPARα in human PBMCs remains unclear. Therefore, we tried to elucidate the function of PPARα in human PBMCs by whole genome microarray analysis of the PBMCs incubated with the specific PPARα ligand WY14,643. Furthermore, to examine the complete role of PPARα within PBMCs during fasting, we compared microarray analysis of PBMCs activated with WY14,643, with microarray analysis of PBMCs during 24 hours of fasting.

## Results

### PPARα regulation in PBMCs after incubation with WY14,643

**Figure 1:**
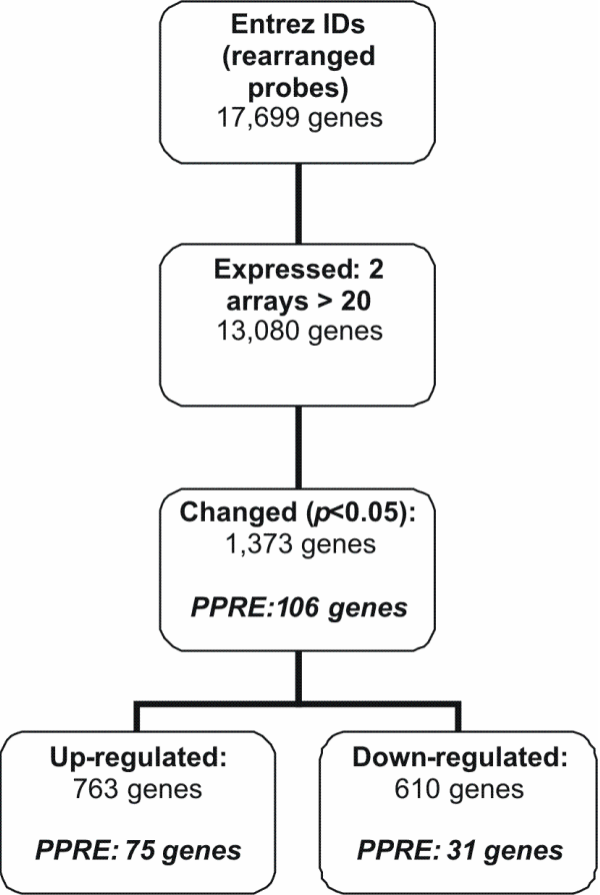
**Gene selection procedure after microarray analysis of WY14,643 incubated PBMCs**. Flow chart of the followed gene selection procedure after microarray analysis of WY14,643 incubated PBMCs from 6 donors. PPRE; number of genes containing a peroxisome proliferator response element according to Lemay *et al*.

Incubation of PBMC with the specific PPARα ligand WY14,643 for 12 hours resulted in a differential expression of 1,373 of the 13,080 genes expressed in the PBMCs, indicating a PPARα-dependent regulation of 10.5% of the genes expressed in PBMC (Figure 1). More than half of these genes (56%) were up-regulated. Pathway analysis of the genes changed upon activation of PPARα with WY14,643, showed a marked increase in fatty acid metabolism, primarily in β-oxidation, and a decrease in several amino acid metabolism pathways (data not shown).

**Figure 2:**
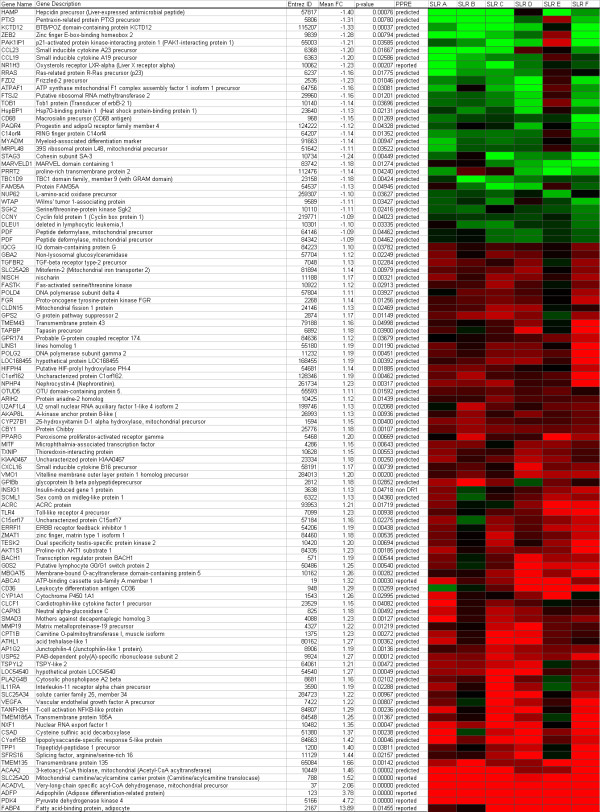
**Genes changed after incubation with WY14,643, containing a predicted or reported PPRE**. Heatmap of the signal log ratio of genes changed upon incubation with WY14,643 that contained a predicted or reported PPRE. Red indicates up-regulation compared to the vehicle incubated PBMCs and green indicates down-regulation. SL R, signal log ratio; PPRE, peroxisome proliferator response element

**Figure 3:**
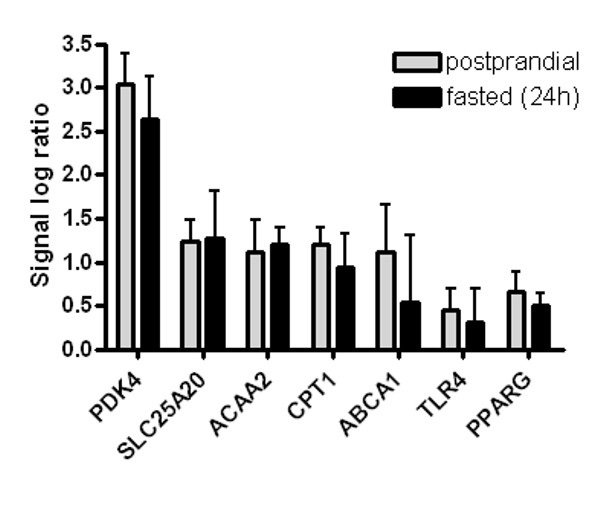
**Gene expression changes of PBMCs incubated with WY14,643 isolated postprandial or after an overnight fast**. Mean gene expression changes of PBMCs incubated with WY14,643, isolated postprandial or after an overnight fast. Error bars indicate standard deviations. *PDK4, Pyruvate dehydrogenase kinase 4; SLC25A20, carnitine-acylcarnitine translocase; ACAA2, acetyl-Coenzyme A acyltransferase 2; CPT1, Carnitine palmitoyltransferase 1; ABCA1, ATP binding cassette transporter 1; TLR4, Toll-like receptor-4; PPARγ, peroxisome proliferator-activated receptor gamma*.

A peroxisome proliferator response elements (PPREs) was ascribed to 106 out of the 1,373 genes changed, using the study of Lemay *et al *[15]. Of these genes, 75 were up-regulated and 31 were down-regulated (Figure 2). Figure 2 shows the responses to activation of PPARα for each person by illustrating the changes in gene expression of these 106 genes per individual. For several genes a clear variation in response upon PPARα activation between individuals is present. Donor B and, especially, donor E show an obvious distinction from the other donors. However, no difference could be found in the expression of *PPARα *between the donors at basal level, and also after incubation with WY14,643 no change in expression of *PPARα *was observed (data not shown). Another reason for the variation could be the difference in concentration of the PPARα ligands, i.e. fatty acids, and other nutritional factors present in the blood during donation. Blood donors are commonly advised to eat before donating blood. To investigate whether the nutritional status influences changes in gene expression, we incubated PBMCs of four volunteers, obtained after a meal and after an overnight fast, with WY14,643. Using QPCR, we determined the changes in PBMC gene expression of genes that showed either a low (*PDK4, SLC25A20, ACAA2*) or a high variation (*CPT1, ABCA1, TLR4, PPARγ*) between donors in the microarray analyzes of the first study (Figure 3). No significant differences were observed in gene expression between the fasted and postprandial state.

To analyze whether the genes that did not have a PPRE according to Lemay *et al*. might have other transcriptional binding sites, a network analysis and a subsequent transcription factor binding site search with Genomatix software was performed. The network analysis showed that, besides the transcription factor PPAR, the transcription factors NFkB, JUN, TP53, SP1 and CTNNB1 were also directly linked to at least 10 genes from the list of 1,373 genes, The subsequent search for binding sites resulted in an additional 122 genes that could be linked to a PPRE and revealed that another 371 genes could be linked to at least one of the other selected transcription factors (see Additional file 1).

**Figure 4:**
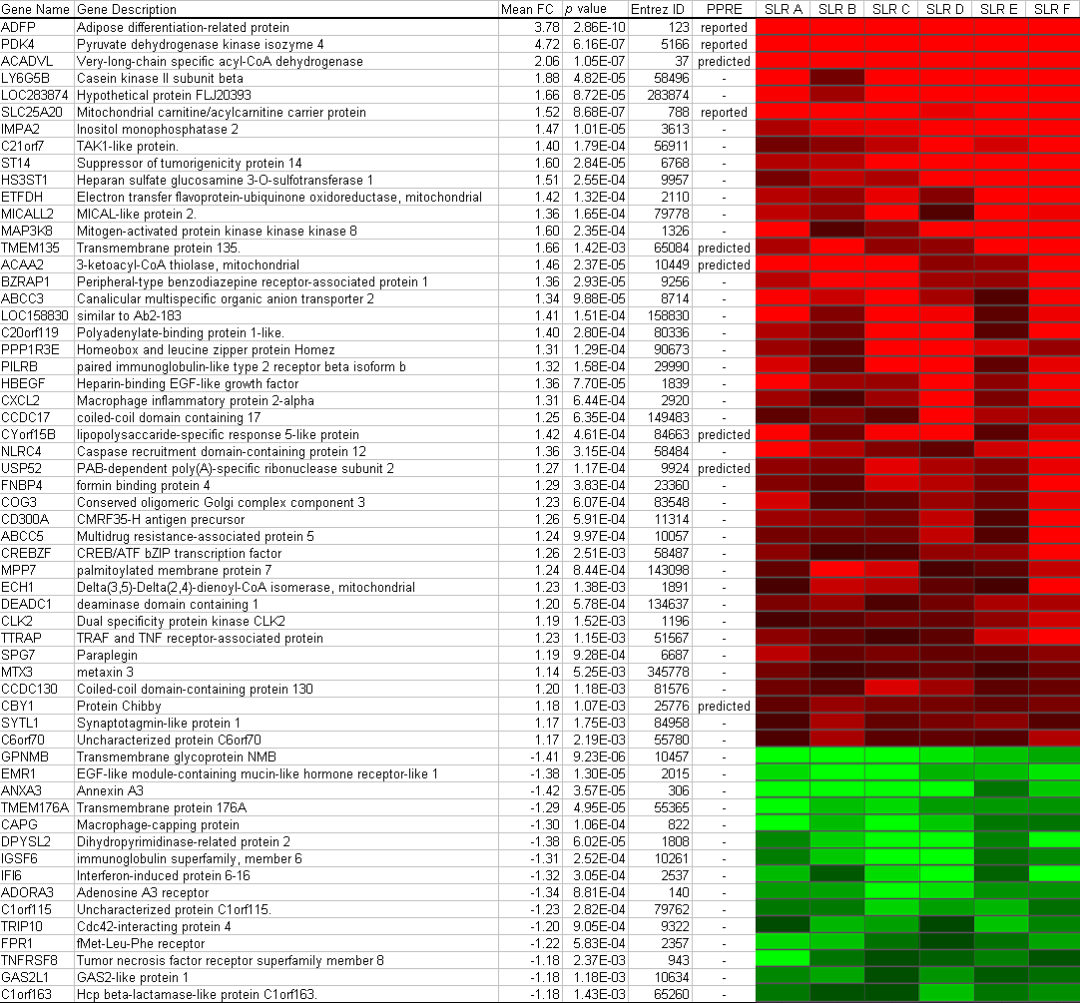
**Genes changed more than 10% in all individuals after incubation with WY14,643**. Heat map of genes changed more than 10% in all individuals after incubation with WY14,643. Red indicates up-regulation and green indicates down-regulation. SLR, signal log ratio; PPRE, peroxisome proliferator response element

To obtain a selection of robust responding genes upon activation of PPARα with WY14.643, genes were selected that were more than 10% up or down regulated in all donors. This resulted in a list of 58 genes, including several known PPARα target genes (*ADFP, PDK4, SLC25A20*) (Figure 4), with a main function in fatty acid β-oxidation. Remarkably, only 16% of the genes in this list contained a predicted or reported PPRE.

**Figure 5:**
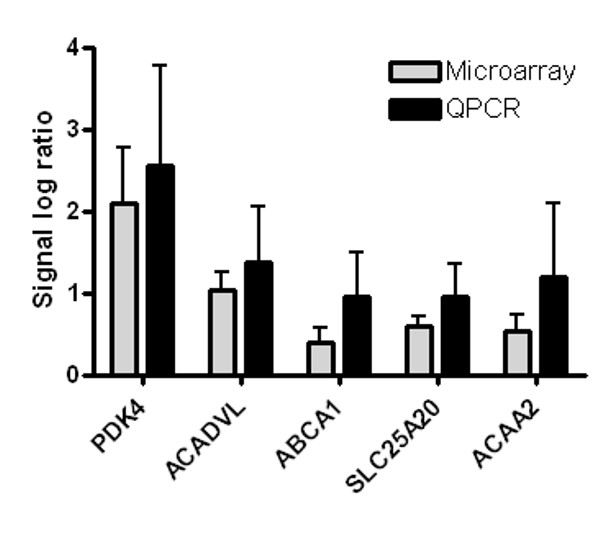
**Comparison of microarray and quantitative real time PCR analysis**. Mean gene expression changes of microarray and quantitative real time PCR analysis (Q-PCR) of six genes after incubation with WY14,643. Error bars indicated standard deviations. *PDK4, Pyruvate dehydrogenase kinase 4; ACADVL, acyl-Coenzyme A dehydrogenase, very long chain ; ABCA1, ATP binding cassette transporter 1; SLC25A20, carnitine-acylcarnitine translocase; ACAA2, acetyl-Coenzyme A acyltransferase 2*.

To validate our data observed with microarray analyzes a selection of genes changed in the microarray analyzes was also measured with quantitative real time PCR (Q-PCR). In concordance with our microarray results, Q-PCR analyzes resulted in similar changes in expression of all genes analyzed (Figure 5).

### PPARα regulation in PBMCs during fasting

**Figure 6:**
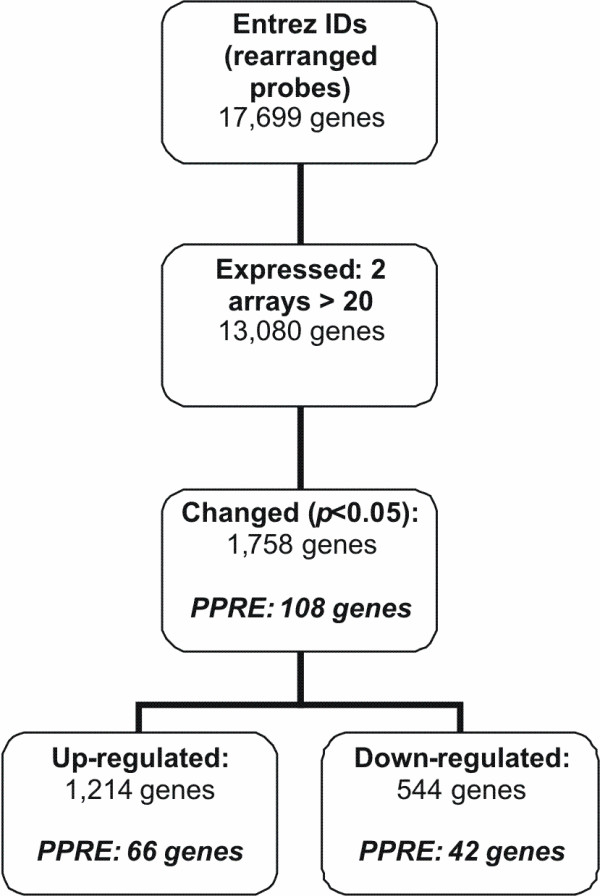
**Gene selection procedure after microarray analysis of PBMC of three 24 hour fasted subjects**. Flow chart of the followed gene selection procedure after microarray analysis of PBMC of three 24 hour fasted subjects. PPRE; number of genes containing a peroxisome proliferator response element according to Lemay *et al*. Data from this fasting study was published previously [14], but has been used here after applying a different annotation procedure.

**Figure 7:**
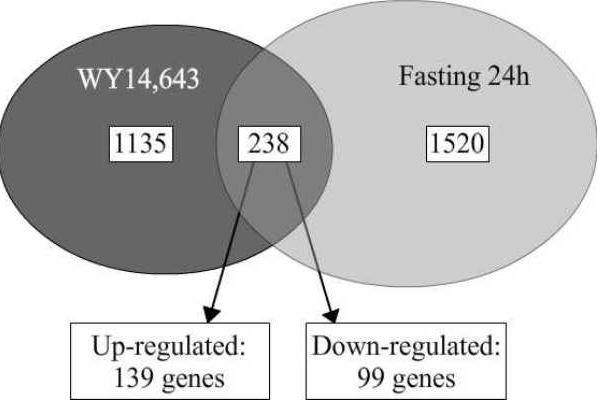
**Overlap between genes changed upon WY14,643 incubation and after 24 hours fasting**. Venn diagram of overlap between genes changed upon WY14,643 incubation and after 24 hours fasting.

Figure 6 shows the genes changed upon 24 hours fasting in healthy human volunteers with the number of genes that contain a PPRE. Comparison of gene expression profiles of PBMCs incubated with the PPARα ligand WY14,643 and fasted for 24 hours resulted in an overlap of 238 genes, indicating that around 14% of the genes changed during fasting are regulated by PPARα (Figure 7). Pathway analysis showed that these 238 genes were primarily involved in fatty acid metabolism. We found no overlap in pathways involved in amino acid metabolism. Exploration of the genes involved in fatty acid metabolism showed that fatty acid β-oxidation was specifically regulated, both in WY14,643 incubated cells and in PBMCs isolated after fasting (data not shown)

## Discussion

In the present study, we showed that activation of the nuclear receptor PPARα in peripheral blood mononuclear cells results in a considerable change in gene expression profiles, as 10.5% of the genes expressed exhibited altered gene expression levels after incubation with the specific PPARα agonist WY14,643. The main function of PPARα in PBMCs appeared to be the regulation of fatty acid β-oxidation and other lipid metabolism related functions, which is in line with results from mice studies in liver [16] and intestine [17], and human cell line studies [18,19]. Moreover, the observed down-regulation of amino acid metabolism has been shown before in liver in studies comparing wild type mice to the PPARα knock out mouse model [7].

Besides the possible roles of PPARα in PBMCs, this study also demonstrates strong individual variability between the subjects in gene expression responses to activation with WY14,643. It appears that each donor has its own specific gene expression profile response to PPARα activation, which results in distinct differences in the expression of certain genes after WY14,643 incubation. Beck *et al*. also reported differences in responsiveness in gene expression between individuals, after incubation of endothelial cells with LPS. However, endothelial cell cultures were already divided beforehand into type I or type II responders based on their LPS mediated IL8 production [20]. In another study, incubation of cultured macrophages with oxidized low-density lipoprotein resulted in a person-specific inflammatory gene expression response that could be correlated to changes in gene expression of scavenger receptors [21]. However, we did not find a correlation between basal *PPARα *expression or changes in *PPARα *expression and the observed variation in gene expression changes. In addition, the differences observed are probably not caused by the nutritional status of the subjects at baseline, as we did not observe differences in expression changes of selected PPARα target genes between the postprandial and the fasted state of PBMCs incubated with WY14,643. However, it should be noted here that only four subjects were studied. A reason for the difference in response of the donors in the first study could be genetic variation, such as single nucleotide polymorphisms (SNPs) in the *PPARα *gene, its target genes or PPARα co-factors involved in activation of gene transcription. Furthermore, epi-genetic variation such as methylation status of the PPARα promoter or its target genes may have caused between-subject differences in gene expression levels. Additional studies are required to elucidate whether gene expression profiles can be clustered in different response profiles, simplifying the identification of factors responsible for these individual responses. With respect to personalized nutrition these individual responses are of great interest as it can be expected that nutrients such as fatty acids can induce similar variations in response as WY14,643, which in the end might lead to personalized dietary advice.

The PPRE analyzes of the genes changed showed that approximately 8% of the genes changed after incubation with the PPARα ligand WY14,643 contained a predicted or reported PPRE, using the list as described by Lemay et al [15]. However, Lemay *et al*. report that they tolerate a low false-positive, and a high (60%) false-negative rate, suggesting that their list of PPREs is far from complete. Our additional transcription factor binding site analysis increase the number of genes that contain a PPRE to a total of 17% of the genes changed. A network search showed that, besides PPAR, five other transcription factors were involved in direct regulation of at least 10 out of the 1,373 changed genes. Interestingly, all these transcription factors are known to be affected by PPARα activation [22-26]. Transcription factor binding site analysis revealed that, out of the changed genes that did not contain a PPRE, 27% contained a binding site for at least one of the other five selected transcription factors These genes appear not to be regulated by PPARα directly, but indirectly, via these other transcription factors, a mechanism which has been suggested before [27,28]. The role of PPARα in this respect seems to be extensively larger than expected based on the outcome of PPRE analyzes alone.

An interesting observation is the decrease in expression level of genes containing a PPRE. Activation of PPARα by a ligand may result in a negative regulation of genes by means of transrepression as has been reported in several studies and reviewed by Ricote and Glass (2007) [29]. This transrepression, however, does not require the presence of PPREs in the promoter regions of the target genes. Apparently, negative regulation of these genes, regardless of its mechanism, is stronger than the transcriptional activation of PPARα. Previously, Degenhardt et al. (2006) also showed down regulation of an insulin-like growth factor-binding protein gene (IGFBP-6) that contained a predicted PPRE, in response to the presence of a PPARα ligand [30].

The overlap in gene expression profiles between fasting and incubation with WY14,643 shows that PPARα in PBMCs carries out a substantial part of its function during fasting, when concentrations of its natural ligands, free fatty acids, are elevated in the blood. The main role of PPARα in PBMCs during fasting is fatty acid β-oxidation, most likely to cope with the reduced availability of glucose for utilization in energy production and the increase of fatty acids.

Direct comparison between the two array analysis should be examined with care, since the two studies are distinctly different in set-up. The fasting intervention study was conducted in vivo, while the WY14,643 incubation experiments were performed ex vivo. Moreover, fasting involves many more changes in physiology, apart from the before-mentioned increase in plasma free fatty acids, including changes in plasma insulin, glucose and leptin concentrations. The PPARα ligand incubations were set-up to elucidate the specific effects of activation of one nuclear factor, controlling for all other parameters.

Summarizing, this study gives us valuable information on the extent of the effect of PPARα activation, during fasting and in general, on human PBMC gene expression. It also shows that persons respond differently to PPARα activation with respect to their gene expression changes, indicating a possible person-specific nutrient response. It seems justified to conclude that human PBMCs are a suitable model to study changes in PPARα activation. This opens up the possibilities for more specific PPARα signaling studies in healthy humans using these relatively easily obtainable blood cells.

## Methods

### PBMC incubation

PBMCs from six healthy Caucasian male blood donors, aged between 30 and 48 yr, were isolated directly after arrival of the buffy coat (max. 8 hours after donation) by Ficol-paque Plus density gradient centrifugation (Amersham Biosciences, Roosendaal, the Netherlands). PBMCs were incubated in RPMI1640 medium with 2 mmol/L L-glutamine, 10% fetal bovine serum and antibiotics (penicillin and streptomycin) in the presence of 5% CO_2 _at 37°C. at 1.0 × 10^6 ^cells per ml with either WY14,643 (50 μM) or vehicle (DMSO, 0.1%) for 12 hours. All donors gave full written informed consent.

### Pre- vs. postprandial incubation

PBMCs of four healthy volunteers, aged between 28 and 34, were isolated after a meal and after an overnight fast. PBMCs were incubated at 1.0 × 10^6 ^cells per ml with either WY14,643 (50 μM) or vehicle (DMSO, 0.1%) for 12 hours. All volunteers gave full written informed consent.

### Statistical methods

A 2-tailed paired *t *test was used to determine significant differences in Q-PCR gene expression values between the postprandial and the fasted state. Statistical significance was accepted at *p *0.05. All calculations were performed with the use of the SPSS (version 12.0.1; SPSS, Chicago, IL).

### Microarray processing

For 6 donors of the incubation experiments, total RNA from PBMCs was labeled using a one-cycle cDNA labeling kit (Affymetrix Inc, Santa Clara, CA) and hybridized to Affymetrix Human whole genome U133 plus 2.0 arrays (Affymetrix). Sample labeling, hybridization to chips and image scanning was performed according to the manufacturer's GeneChip Expression Analysis Technical Manual (Affymetrix).

### Intervention study

For comparison of microarray data of the abovementioned incubation study, with microarray data of PBMC of fasted volunteers, we used the earlier described microarray data of a 48 hours fasting study [14]. Briefly, four healthy male Caucasian volunteers, between 19 and 22 year of age were fasted for 48 hours. PBMCs were isolated out of blood taken at baseline, after 24 hours and after 48 hours of fasting. All volunteers gave full written informed consent and the study protocol was approved by the medical ethics committee of Wageningen University.

### Microarray analysis

Microarrays were analyzed using the reorganized oligonucleotide probes as described by Dai *et al *(2005) [31]. Dai *et al*. combined all individual probes for a gene, enabling the possibility to detect the overall transcription activity of a gene, based on the latest genome and transcriptome information, instead of the Affymetrix probe set annotation. Application of this annotation procedure on the previously published data from the 48 hours fasting study [14] resulted in a difference in number of genes expressed and changed as compared to the previously used annotation method as this analysis was performed on probe set level.

Expression values were calculated using the Robust Multichip Average (RMA) method. RMA signal value estimates are based on a robust average of background corrected perfect match intensities and normalization was performed using quantile normalization [32,33]. Only genes with normalized signals higher then 20 on at least two out of twelve arrays were defined as expressed and selected for further analysis. Genes were defined as 'changed' when comparison of the normalized signal intensities showed a p-value lower then 0.05 in a two-tailed paired t test with Bayesian correction (Limma) [34]. Pathway analysis was performed using Ingenuity Pathway Analysis 5.5 (Ingenuity Systems). Array data have been submitted to the Gene Expression Omnibus, accession number GSE11289.

### PPRE incidence

To indicated which of the genes changed upon activation of PPARα had a predicted or reported peroxisome proliferator response element (PPRE), we used information from Lemay *et al*. [15]. This paper recently reported predicted PPRE on a genome wide scale, using computational genomics and also summarized known PPRE. Using Genomatix software [35], network analysis was performed on 1,373 genes in BiblioSphere, from which transcription factors were selected that were directly linked to more than 10 genes from our list of changed genes. Subsequent transcription factor binding site analysis identified transcription factor binding sites in the promoters of our genes of interest that were cocited at least once in an abstract with these transcription factors. Heat maps were created by using Spotfire software.

### cDNA synthesis and quantitative real-time PCR

RNA was reverse transcribed with the use of the cDNA synthesis kit (Promega, Leiden, the Netherlands). Standard quantitative real-time polymerase chain reaction (Q-PCR) was performed with the use of Platinum Taq DNA polymerase and SYBR Green on an iCycler PCR machine (Bio-Rad Laboratories BV) and duplicated at least twice. Primer sequences used in the PCRs were chosen based on the sequences available in PRIMERBANK [36]. Q-PCR data were normalized by measuring cycle threshold ratios between candidate genes and a housekeeping gene, human acidic ribosomal phosphoprotein PO, which was shown to be consistent within PBMCs [37].

## Authors' contributions

MB collected and analyzed the data and wrote the manuscript. LAA an MM participated in critical revising of the manuscript. None of the authors has a personal or financial conflict of interest.

## Supplementary Material

Additional file 1 **Transcription factor binding site analysis**. Presence of transcription factor binding sites in the genes changed in PBMC after incubation with WY14,643. Transcription factors were selected if they directly affected at least 10 genes that were changed after WY14,643 incubation, in a network search using BiblioSphere (Genomatix). FC, fold change; PPRE (Lemay), peroxisome proliferator response element according to Lemay *et al*.[15]; NFkB, Nuclear factor kappa B binding site; JUN, Jun oncogene binding site; TP53, Tumor protein 53 binding site; SP1, Specificity protein 1 binding site; CTNNB1, catenin beta 1 binding site. Red indicates up regulated, green indicates down regulated Click here for file
