# Supplementary material for: Activation of peroxisome proliferator-activated receptor alpha in human peripheral blood mononuclear cells reveals an individual gene expression profile response
Source: BMC Genomics. 2008 Jun 2;9:262. doi: 10.1186/1471-2164-9-262 (PMC2430976; doi:10.1186/1471-2164-9-262)
Supplement: Additional file 1 — Transcription factor binding site analysis. Presence of transcription factor binding sites in the genes changed in PBMC after incubation with WY14,643. Transcription factors were selected if they directly affected at least 10 genes that were changed after WY14,643 incubation, in a network search using BiblioSphere (Genomatix). FC, fold change; PPRE (Lemay), peroxisome proliferator response element according to Lemay et al.[15]; NFkB, Nuclear factor kappa B binding site; JUN, Jun oncogene binding site; TP53, Tumor protein 53 binding site; SP1, Specificity protein 1 binding site; CTNNB1, catenin beta 1 binding site. Red indicates up regulated, green indicates down regulated [file 1471-2164-9-262-S1.pdf]

Gene Name	Gene Description	Entrez ID	Mean FC	*p*-value	PPRE (Lemay)	PPRE	NFkB	JUN	TP53	SP1	CTNNB1
VEGFA	VASCULAR ENDOTHELIAL GROWTH FACTOR	7422	1.221779	0.0080749	X	X	X	X	X	X	X
TLR4	TOLL-LIKE RECEPTOR 4	7099	1.234441	0.0093773	X	X	X	X	X	X	X
PPARG	PEROXISOME PROLIFERATIVE ACTIVATED RECEPTOR, GAMMA	5468	1.201591	0.0066852	X	X	X	X	X	X	X
FABP4	FATTY ACID BINDING PROTEIN 4, ADIPOCYTE	2167	13.88884	0.0145549	X	X	X	X	X	X	X
CYP1A1	CYTOCHROME P450, FAMILY 1, SUBFAMILY A, POLYPEPTIDE 1	1543	1.259719	0.0299543	X	X	X	X	X	X	X
SMAD3	SMAD, MOTHERS AGAINST DPP HOMOLOG 3 (DROSOPHILA)	4088	1.230543	0.0012659	X	X	X	X	X	X	X
TGFBR2	TRANSFORMING GROWTH FACTOR, BETA RECEPTOR II (70/80KDA)	7048	1.129787	0.0228447	X	X	X	X	X	X	X
FGR	GARDNER-RASHEED FELINE SARCOMA VIRAL (V-FGR) ONCOGENE HOMOLOG	2268	1.140862	0.0125577	X	X	X	X	X	X	-
MITF	MICROPHTHALMIA-ASSOCIATED TRANSCRIPTION FACTOR	4286	1.15412	0.006431	X	X	X	X	X	-	X
CD36	CD36 ANTIGEN (COLLAGEN TYPE I RECEPTOR, THROMBOSPONDIN RECEPTOR)	948	1.291836	0.032585	X	X	X	X	X	-	-
TXNIP	THIOREDOXIN INTERACTING PROTEIN	10628	1.148212	0.0055335	X	X	X	X	X	-	-
PDK4	PYRUVATE DEHYDROGENASE KINASE, ISOZYME 4	5166	4.715723	6.16E-07	X	X	X	X	-	X	-
ABCA1	ATP-BINDING CASSETTE, SUB-FAMILY A (ABC1), MEMBER 1	19	1.319921	0.0002982	X	X	X	X	-	X	-
ADFP	ADIPOSE DIFFERENTIATION-RELATED PROTEIN	123	3.783926	2.86E-10	X	X	X	X	-	-	X
SLC25A20	SOLUTE CARRIER FAMILY 25 (CARNITINE/ACYLCARNITINE TRANSLOCASE), MEMBER 20	788	1.521131	8.68E-07	X	X	-	X	X	X	-
ACADVL	ACYL-COENZYME A DEHYDROGENASE, VERY LONG CHAIN	37	2.058207	1.05E-07	X	X	-	-	X	X	X
CXCL16	CHEMOKINE (C-X-C MOTIF) LIGAND 16	58191	1.165929	0.007387	X	X	X	X	-	-	-
MMP19	MATRIX METALLOPROTEINASE 18	4327	1.217718	0.0121907	X	X	X	X	-	-	-
INSIG1	INSULIN INDUCED GENE 1	3638	1.12813	0.0471767	X	X	X	-	X	-	-
CYP27B1	CYTOCHROME P450, FAMILY 27, SUBFAMILY B, POLYPEPTIDE 1	1594	1.150047	0.0039956	X	X	-	X	-	X	-
CPT1B	CARNITINE PALMITOYLTRANSFERASE 1B (MUSCLE)	1375	1.228796	0.0027246	X	X	-	-	-	-	-
CSAD	CYSTEINE SULFINIC ACID DECARBOXYLASE	51380	1.374212	0.0023752	X	X	-	-	-	-	-
MBOAT5	O-ACYLTRANSFERASE (MEMBRANE BOUND) DOMAIN CONTAINING 5	10162	1.264312	0.0028218	X	X	-	-	-	-	-
SLC25A28	SOLUTE CARRIER FAMILY 25, MEMBER 28	81894	1.140181	0.0097874	X	X	-	-	-	-	-
TSPYL2	TSPY-LIKE 2	64061	1.205101	0.0047185	X	-	X	X	X	-	X
CAPN3	CALPAIN 3, (P94)	825	1.175736	0.0049199	X	-	X	X	X	-	X
TAPBP	TAP BINDING PROTEIN (TAPASIN)	6892	1.181845	0.0390021	X	-	X	X	-	X	-
ERRFI1	ERBB RECEPTOR FEEDBACK INHIBITOR 1	54206	1.192953	0.0043822	X	-	X	X	-	-	-
CBY1	PKD2 INTERACTOR, GOLGI AND ENDOPLASMIC RETICULUM ASSOCIATED 1	25776	1.175397	0.0010717	X	-	X	-	-	-	X
BACH1	BTB AND CNC HOMOLOGY 1, BASIC LEUCINE ZIPPER TRANSCRIPTION FACTOR 1	571	1.194431	0.0054392	X	-	-	X	X	-	-
GPS2	G PROTEIN PATHWAY SUPPRESSOR 2	2874	1.170868	0.0114851	X	-	-	X	X	-	-
IL11RA	INTERLEUKIN 11 RECEPTOR, ALPHA	3590	1.193033	0.0228847	X	-	-	X	-	X	-
NISCH	NISCHARIN	11188	1.171259	0.0032111	X	-	X	-	-	-	-
CLCF1	CARDIOTROPHIN-LIKE CYTOKINE FACTOR 1	23529	1.15456	0.0408161	X	-	X	-	-	-	-
AP1G2	ADAPTOR-RELATED PROTEIN COMPLEX 1, GAMMA 2 SUBUNIT	8906	1.193766	0.0013565	X	-	-	X	-	-	-
G0S2	G0/G1SWITCH 2	50486	1.254146	0.0054033	X	-	-	-	X	-	-
FASTK	FAS-ACTIVATED SERINE/THREONINE KINASE	10922	1.115629	0.0291284	X	-	-	-	X	-	-
LINS1	LINES HOMOLOG 1 (DROSOPHILA)	55180	1.187408	0.0118996	X	-	-	-	-	-	X
TMEM135	HYPOTHETICAL PROTEIN FLJ22104	65084	1.663445	0.001419	X	-	-	-	-	-	-
ACAA2	ACETYL-COENZYME A ACYLTRANSFERASE 2 (MITOCHONDRIAL 3-OXOACYL-COENZYME A THIOLASE)	10449	1.459782	2.37E-05	X	-	-	-	-	-	-
SFRS16	SPLICING FACTOR, ARGININE/SERINE-RICH 16 (SUPPRESSOR-OF-WHITE-APRICOT HOMOLOG, DROSOPHILA)	11129	1.435275	0.0215718	X	-	-	-	-	-	-
CYorf15B	CHROMOSOME Y OPEN READING FRAME 15B	84663	1.415708	0.0004614	X	-	-	-	-	-	-
TPP1	TRIPEPTIDYL PEPTIDASE I	1200	1.395244	0.0381088	X	-	-	-	-	-	-
NXF1	NUCLEAR RNA EXPORT FACTOR 1	10482	1.353484	0.0004731	X	-	-	-	-	-	-
	T-CELL ACTIVATION NFKB-LIKE PROTEIN	84807	1.287299	0.0023648	X	-	-	-	-	-	-
ATHL1	ATH1, ACID TREHALASE-LIKE 1 (YEAST)	80162	1.272204	0.0036191	X	-	-	-	-	-	-
USP52	UBIQUITIN SPECIFIC PEPTIDASE 52	9924	1.26817	0.0001171	X	-	-	-	-	-	-
FLJ10404	HYPOTHETICAL PROTEIN FLJ10404	54540	1.267159	0.0004941	X	-	-	-	-	-	-
TMEM185A	FAMILY WITH SEQUENCE SIMILARITY 11, MEMBER A	84548	1.24973	0.0136671	X	-	-	-	-	-	-
NPHP4	KIAA0673 PROTEIN	261734	1.231587	0.0031745	X	-	-	-	-	-	-
AKT1S1	AKT1 SUBSTRATE 1 (PROLINE-RICH)	84335	1.225616	0.0018536	X	-	-	-	-	-	-
SLC25A34	SOLUTE CARRIER FAMILY 25, MEMBER 34	284723	1.217161	0.0096719	X	-	-	-	-	-	-
ACRC	ACIDIC REPEAT CONTAINING	93953	1.209953	0.0171939	X	-	-	-	-	-	-
TESK2	TESTIS-SPECIFIC KINASE 2	10420	1.204855	0.0069398	X	-	-	-	-	-	-
VMO1	VITELLINE MEMBRANE OUTER LAYER 1 HOMOLOG (CHICKEN)	284013	1.198301	0.0020021	X	-	-	-	-	-	-
POLG2	POLYMERASE (DNA DIRECTED), GAMMA 2, ACCESSORY SUBUNIT	11232	1.192566	0.0045133	X	-	-	-	-	-	-
C1orf162	CHROMOSOME 1 OPEN READING FRAME 162	128346	1.192297	0.004622	X	-	-	-	-	-	-
NA	HYPOTHETICAL PROTEIN FLJ36031	168455	1.19209	0.0039225	X	-	-	-	-	-	-
NA	GLYCOPROTEIN IB (PLATELET), BETA POLYPEPTIDE	2812	1.181017	0.0285219	X	-	-	-	-	-	-
ZMAT1	ZINC FINGER, MATRIN TYPE 1	84460	1.17932	0.0053479	X	-	-	-	-	-	-
KIAA0467	KIAA0467	23334	1.176629	0.0024951	X	-	-	-	-	-	-
PLA2G4B	PHOSPHOLIPASE A2, GROUP IVB (CYTOSOLIC)	8681	1.162921	0.0210223	X	-	-	-	-	-	-
C15orf17	CHROMOSOME 15 OPEN READING FRAME 17	57184	1.161011	0.0227504	X	-	-	-	-	-	-
TMEM43	TRANSMEMBRANE PROTEIN 43	79188	1.160679	0.0499804	X	-	-	-	-	-	-
	HYPOXIA-INDUCIBLE FACTOR PROLYL 4-HYDROXYLASE	54681	1.136526	0.0188489	X	-	-	-	-	-	-
SCML1	SEX COMB ON MIDLEG-LIKE 1 (DROSOPHILA)	6322	1.134464	0.0435967	X	-	-	-	-	-	-
U2AF1L4	U2(RNU2) SMALL NUCLEAR RNA AUXILIARY FACTOR 1-LIKE 3	199746	1.133753	0.0206752	X	-	-	-	-	-	-
AKAP8L	A KINASE (PRKA) ANCHOR PROTEIN 8-LIKE	26993	1.133022	0.0093551	X	-	-	-	-	-	-
CLDN15	CLAUDIN 15	24146	1.12813	0.0246879	X	-	-	-	-	-	-
GPR174	G PROTEIN-COUPLED RECEPTOR 174	84636	1.117606	0.036795	X	-	-	-	-	-	-
GBA2	GLUCOSIDASE, BETA (BILE ACID) 2	57704	1.116318	0.0224914	X	-	-	-	-	-	-
ARIH2	ARIADNE HOMOLOG 2 (DROSOPHILA)	10425	1.115828	0.0143872	X	-	-	-	-	-	-
POLD4	POLYMERASE (DNA-DIRECTED), DELTA 4	57804	1.111602	0.0392711	X	-	-	-	-	-	-
OTUD5	OTU DOMAIN CONTAINING 5	55593	1.111158	0.0159154	X	-	-	-	-	-	-
IQCG	HYPOTHETICAL PROTEIN FLJ23571	84223	1.096807	0.0378196	X	-	-	-	-	-	-
CD68	CD68 ANTIGEN	968	-1.153985	0.0126924	X	X	X	X	X	-	-
NR1H3	NUCLEAR RECEPTOR SUBFAMILY 1, GROUP H, MEMBER 3	10062	-1.230728	0.0020667	X	X	X	X	-	-	-
CCL19	CHEMOKINE (C-C MOTIF) LIGAND 19	6363	-1.195769	0.0258567	X	X	X	-	-	X	-
ZEB2	ZINC FINGER HOMEOBOX 1B	9839	-1.27539	0.0079351	X	-	X	X	X	-	X
CCL23	CHEMOKINE (C-C MOTIF) LIGAND 23	6368	-1.203001	0.0166702	X	-	X	X	X	-	-
NUP62	INTERLEUKIN 4 INDUCED 1	259307	-1.104729	0.036268	X	-	X	-	X	-	-
HAMP	HEPCIDIN ANTIMICROBIAL PEPTIDE	57817	-1.401492	0.000765	X	-	-	X	X	-	-
PAK1IP1	PAK1 INTERACTING PROTEIN 1	55003	-1.210652	0.0358472	X	-	X	-	-	-	-
PTX3	PENTRAXIN-RELATED GENE, RAPIDLY INDUCED BY IL-1 BETA	5806	-1.310949	0.0077976	X	-	X	-	-	-	-
NA	DELETED IN LYMPHOCYTIC LEUKEMIA, 1	10301	-1.104861	0.0333512	X	-	-	-	X	-	-
TOB1	TRANSDUCER OF ERBB2, 1	10140	-1.139279	0.036956	X	-	-	-	X	-	-
STAG3	STROMAL ANTIGEN 3	10734	-1.235672	0.0044916	X	-	-	-	X	-	-
C14orf4	CHROMOSOME 14 OPEN READING FRAME 4	64207	-1.144712	0.0135168	X	-	-	-	-	X	-
FZD2	FRIZZLED HOMOLOG 2 (DROSOPHILA)	2535	-1.229252	0.0104611	X	-	-	-	-	-	X
CCNY	CHROMOSOME 10 OPEN READING FRAME 9	219771	-1.090363	0.040228	X	-	-	-	-	-	-
PDF	PEPTIDE DEFORMYLASE-LIKE PROTEIN	64146	-1.092063	0.0446236	X	-	-	-	-	-	-
PDF	COMPONENT OF OLIGOMERIC GOLGI COMPLEX 8	84342	-1.092063	0.0446236	X	-	-	-	-	-	-
SGK2	SERUM/GLUCOCORTICOID REGULATED KINASE 2	10110	-1.10771	0.0241608	X	-	-	-	-	-	-
MRPL48	MITOCHONDRIAL RIBOSOMAL PROTEIN L48	51642	-1.112999	0.0352178	X	-	-	-	-	-	-
WTAP	WILMS TUMOR 1 ASSOCIATED PROTEIN	9589	-1.113961	0.0342689	X	-	-	-	-	-	-
PAQR4	PROGESTIN AND ADIPOQ RECEPTOR FAMILY MEMBER IV	124222	-1.11501	0.0432815	X	-	-	-	-	-	-
FAM35A	FAMILY WITH SEQUENCE SIMILARITY 35, MEMBER A	54537	-1.125351	0.0494486	X	-	-	-	-	-	-
	HSP70-INTERACTING PROTEIN	23640	-1.133021	0.0213065	X	-	-	-	-	-	-
PRRT2	PROLINE-RICH TRANSMEMBRANE PROTEIN 2	112476	-1.14348	0.0423987	X	-	-	-	-	-	-
MYADM	MYELOID-ASSOCIATED DIFFERENTIATION MARKER	91663	-1.144163	0.0094688	X	-	-	-	-	-	-
FTSJ2	FTSJ HOMOLOG 2 (E. COLI)	29960	-1.155742	0.0120124	X	-	-	-	-	-	-
ATPAF1	ATP SYNTHASE MITOCHONDRIAL F1 COMPLEX ASSEMBLY FACTOR 1	64756	-1.155908	0.0308137	X	-	-	-	-	-	-
RRAS	RELATED RAS VIRAL (R-RAS) ONCOGENE HOMOLOG	6237	-1.16246	0.0177454	X	-	-	-	-	-	-
NA	MARVEL DOMAIN CONTAINING 1	83742	-1.178018	0.012738	X	-	-	-	-	-	-
TBC1D9	TBC1 DOMAIN FAMILY, MEMBER 9	23158	-1.182574	0.0042362	X	-	-	-	-	-	-
KCTD12	POTASSIUM CHANNEL TETRAMERISATION DOMAIN CONTAINING 12	115207	-1.330155	0.000366	X	-	-	-	-	-	-
IL1A	INTERLEUKIN 1, ALPHA	3552	1.580047	0.0130165	-	X	X	X	X	X	X
IKBKB	INHIBITOR OF KAPPA LIGHT POLYPEPTIDE GENE ENHANCER IN B-CELLS, KINASE BETA	3551	1.206644	0.0106094	-	X	X	X	X	X	X
CAT	CATALASE	847	1.178474	0.0334862	-	X	X	X	X	X	X
BCL6	B-CELL CLL/LYMPHOMA 6 (ZINC FINGER PROTEIN 51)	604	1.16478	0.0069348	-	X	X	X	X	X	X
RELA	V-REL RETICULOENDOTHELIOSIS VIRAL ONCOGENE HOMOLOG A, NUCLEAR FACTOR OF KAPPA LIGHT POLYPEPTIDE GENE ENHANCER IN B-CELLS 3, P65 (AVIAN)	5970	1.132497	0.0202377	-	X	X	X	X	X	X
CDKN1A	CYCLIN-DEPENDENT KINASE INHIBITOR 1A (P21, CIP1)	1026	1.095638	0.0488206	-	X	X	X	X	X	X

Gene Name	Gene Description	Entrez ID	Mean FC	*p*- value	PPRE (Lemay)	PPRE	NFkB	JUN	TP53	SP1	CTNNB1
ATF3	ACTIVATING TRANSCRIPTION FACTOR 3	467	1.194593	0.0019194	-	X	X	X	X	X	X
LDLR	LOW DENSITY LIPOPROTEIN RECEPTOR (FAMILIAL HYPERCHOLESTEROLEMIA)	3949	1.164872	0.0080429	-	X	X	X	X	X	X
TNFRSF10B	TUMOR NECROSIS FACTOR RECEPTOR SUPERFAMILY, MEMBER 10B	8795	1.147862	0.0165286	-	X	X	X	X	X	X
G6PD	GLUCOSE-6-PHOSPHATE DEHYDROGENASE	2539	1.12554	0.0128427	-	X	X	X	X	X	X
JAK1	JANUS KINASE 1 (A PROTEIN TYROSINE KINASE)	3716	1.234495	0.0107605	-	X	X	X	X	X	X
STAT5B	SIGNAL TRANSDUCER AND ACTIVATOR OF TRANSCRIPTION 5B	6777	1.125936	0.0230477	-	X	X	X	X	X	X
MUC2	MUCIN 2, INTESTINAL/TRACHEAL	4583	1.119772	0.0345983	-	X	X	X	X	X	X
SAFB	SCAFFOLD ATTACHMENT FACTOR B	6294	1.106228	0.0308906	-	X	X	X	X	X	X
TIMP1	TIMP METALLOPEPTIDASE INHIBITOR 1	7076	1.097179	0.0327067	-	X	X	X	X	X	X
LCK	LYMPHOCYTE-SPECIFIC PROTEIN TYROSINE KINASE	3932	1.097059	0.0330428	-	X	X	X	X	X	X
HBEGF	HEPARIN-BINDING EGF-LIKE GROWTH FACTOR	1839	1.357261	7.70E-05	-	X	X	X	X	X	X
TYK2	TYROSINE KINASE 2	7297	1.186942	0.0045561	-	X	X	X	X	X	X
ADAM17	ADAM METALLOPEPTIDASE DOMAIN 17 (TUMOR NECROSIS FACTOR, ALPHA, CONVERTING ENZYME)	6868	1.148641	0.047253	-	X	X	X	X	X	X
SHC1	SHC (SRC HOMOLOGY 2 DOMAIN CONTAINING) TRANSFORMING PROTEIN 1	6464	1.137184	0.0241625	-	X	X	X	X	X	X
PPP1R15A	PROTEIN PHOSPHATASE 1, REGULATORY (INHIBITOR) SUBUNIT 15A	23645	1.109591	0.0319806	-	X	X	X	X	X	X
ITGAX	INTEGRIN, ALPHA X (COMPLEMENT COMPONENT 3 RECEPTOR 4 SUBUNIT)	3687	1.280605	0.0008406	-	X	X	X	X	X	-
ATF2	ACTIVATING TRANSCRIPTION FACTOR 2	1386	1.253265	0.0471235	-	X	X	X	X	X	-
TLR2	TOLL-LIKE RECEPTOR 2	7097	1.236468	0.0071802	-	X	X	X	X	X	-
CDK2	CYCLIN-DEPENDENT KINASE 2	1017	1.175347	0.011076	-	X	X	X	X	X	-
MCL1	MYELOID CELL LEUKEMIA SEQUENCE 1 (BCL2-RELATED)	4170	1.131151	0.0211987	-	X	X	X	X	X	-
STAT6	SIGNAL TRANSDUCER AND ACTIVATOR OF TRANSCRIPTION 6, INTERLEUKIN-4 INDUCED	6778	1.111134	0.0302022	-	X	X	X	X	X	-
ABCC3	ATP-BINDING CASSETTE, SUB-FAMILY C (CFTR/MRP), MEMBER 3	8714	1.34346	9.88E-05	-	X	X	X	X	X	-
DUSP1	DUAL SPECIFICITY PHOSPHATASE 1	1843	1.273508	0.0150068	-	X	X	X	X	X	-
CPT1A	CARNITINE PALMITOYLTRANSFERASE 1A (LIVER)	1374	1.234261	0.0019691	-	X	X	X	X	X	-
KLF10	KRUPPEL-LIKE FACTOR 10	7071	1.239398	0.0085498	-	X	X	X	X	X	-
AKR1C1	ALDO-KETO REDUCTASE FAMILY 1, MEMBER C1 (DIHYDRODIOL DEHYDROGENASE 1; 20-ALPHA (3-ALPHA)-HYDROXYSTEROID DEHYDROGENASE)	1645	1.195878	0.0021747	-	X	X	X	X	X	-
NA	THIOREDOXIN REDUCTASE 1	7296	1.184046	0.0061542	-	X	X	X	X	X	-
AKR1B1	ALDO-KETO REDUCTASE FAMILY 1, MEMBER B1 (ALDOSE REDUCTASE)	231	1.110223	0.0397764	-	X	X	X	X	X	-
SCD	STEAROYL-COA DESATURASE (DELTA-9-DESATURASE)	6319	1.151128	0.0308351	-	X	X	X	-	X	X
PDE3B	PHOSPHODIESTERASE 3B, CGMP-INHIBITED	5140	1.479661	0.0074567	-	X	X	-	X	X	X
VCAN	CHONDROITIN SULFATE PROTEOGLYCAN 2 (VERSICAN)	1462	1.386547	0.0047469	-	X	-	X	X	X	X
FMOD	FIBROMODULIN	2331	1.12505	0.0393231	-	X	-	X	X	X	X
MAPK7	MITOGEN-ACTIVATED PROTEIN KINASE 7	5598	1.117557	0.0378458	-	X	X	X	X	-	-
OLR1	OXIDISED LOW DENSITY LIPOPROTEIN (LECTIN-LIKE) RECEPTOR 1	4973	1.426989	0.007251	-	X	X	X	X	-	-
CTSK	CATHEPSIN K (PYCNODYSOSTOSIS)	1513	1.262247	0.0010452	-	X	X	X	X	-	-
GSR	GLUTATHIONE REDUCTASE	2936	1.178142	0.0344841	-	X	X	X	X	-	-
HDC	HISTIDINE DECARBOXYLASE	3067	1.286056	0.0014223	-	X	X	X	-	X	-
GCN5L2	GCN5 GENERAL CONTROL OF AMINO-ACID SYNTHESIS 5-LIKE 2 (YEAST)	2648	1.100095	0.029188	-	X	X	-	X	X	-
HSP90AB1	HEAT SHOCK PROTEIN 90KDA ALPHA (CYTOSOLIC), CLASS B MEMBER 1	3326	1.099833	0.0463645	-	X	-	X	X	X	-
MECP2	METHYL CPG BINDING PROTEIN 2 (RETT SYNDROME)	4204	1.091365	0.0440683	-	X	-	X	X	X	-
IL23A	INTERLEUKIN 23, ALPHA SUBUNIT P19	51561	1.143005	0.0232282	-	X	X	X	-	-	-
CXCL2	CHEMOKINE (C-X-C MOTIF) LIGAND 2	2920	1.308054	0.0006436	-	X	X	X	-	-	-
EDG4	ENDOTHELIAL DIFFERENTIATION, LYSOPHOSPHATIDIC ACID G-PROTEIN-COUPLED RECEPTOR, 4	9170	1.174263	0.003865	-	X	X	X	-	-	-
S100A13	S100 CALCIUM BINDING PROTEIN A13	6284	1.13045	0.0121952	-	X	X	-	X	-	-
LTB4R	LEUKOTRIENE B4 RECEPTOR	1241	1.170665	0.0167303	-	X	X	-	-	X	-
SETDB1	SET DOMAIN, BIFURCATED 1	9869	1.093947	0.0441637	-	X	X	-	-	X	-
CXCL5	CHEMOKINE (C-X-C MOTIF) LIGAND 5	6374	2.320665	0.0082481	-	X	X	-	-	X	-
LCAT	LECITHIN-CHOLESTEROL ACYLTRANSFERASE	3931	1.182232	0.0163856	-	X	X	-	-	X	-
CUGBP1	CUG TRIPLET REPEAT, RNA BINDING PROTEIN 1	10658	1.180837	0.0023564	-	X	-	X	-	X	-
SLC22A5	SOLUTE CARRIER FAMILY 22 (ORGANIC CATION TRANSPORTER), MEMBER 5	6584	1.17802	0.023073	-	X	-	X	-	X	-
MUTYH	MUTY HOMOLOG (E. COLI)	4595	1.105156	0.0251853	-	X	-	-	X	-	X
RXRB	RETINOID X RECEPTOR, BETA	6257	1.189742	0.0198119	-	X	X	-	-	-	-
TREM1	TRIGGERING RECEPTOR EXPRESSED ON MYELOID CELLS 1	54210	1.375605	0.0025191	-	X	X	-	-	-	-
SCARB2	SCAVENGER RECEPTOR CLASS B, MEMBER 2	950	1.351224	0.0231159	-	X	-	X	-	-	-
GPR77	G PROTEIN-COUPLED RECEPTOR 77	27202	1.135725	0.0458415	-	X	-	X	-	-	-
SQLE	SQUALENE EPOXIDASE	6713	1.130551	0.0230116	-	X	-	-	-	X	-
STX4	SYNTAXIN 4A (PLACENTAL)	6810	1.16156	0.0027387	-	X	-	-	-	-	X
ACSL1	FATTY-ACID-COENZYME A LIGASE, LONG-CHAIN 1	2180	1.212256	0.0080522	-	X	-	-	-	-	-
LPIN1	LIPIN 1	23175	1.155458	0.0081319	-	X	-	-	-	-	-
ARRDC3	ARRESTIN DOMAIN CONTAINING 3	57561	1.109565	0.045853	-	X	-	-	-	-	-
NRBP2	NUCLEAR RECEPTOR BINDING PROTEIN 2	340371	1.21139	0.0180779	-	X	-	-	-	-	-
CCNT2	CYCLIN T2	905	1.206694	0.0373391	-	X	-	-	-	-	-
ATG9B	ATG9 AUTOPHAGY RELATED 9 HOMOLOG B (S. CEREVISIAE)	285973	1.153495	0.0317491	-	X	-	-	-	-	-
	APOLIPOPROTEIN B48 RECEPTOR	55911	1.121929	0.0207631	-	X	-	-	-	-	-
IFNG	INTERFERON, GAMMA	3458	-1.102063	0.0291348	-	X	X	X	X	X	X
HSPA5	HEAT SHOCK 70KDA PROTEIN 5 (GLUCOSE-REGULATED PROTEIN, 78KDA)	3309	-1.110691	0.0278061	-	X	X	X	X	X	X
APP	AMYLOID BETA (A4) PRECURSOR PROTEIN (PEPTIDASE NEXIN-II, ALZHEIMER DISEASE)	351	-1.195187	0.0053673	-	X	X	X	X	X	X
SERPINA1	SERPIN PEPTIDASE INHIBITOR, CLADE A (ALPHA-1 ANTIPROTEINASE, ANTITRYPSIN), MEMBER 1	5265	-1.098291	0.0409775	-	X	X	X	X	X	X
MMP9	MATRIX METALLOPEPTIDASE 9 (GELATINASE B, 92KDA GELATINASE, 92KDA TYPE IV COLLAGENASE)	4318	-1.120419	0.0456701	-	X	X	X	X	X	X
MYOD1	MYOGENIC DIFFERENTIATION 1	4654	-1.138861	0.0096635	-	X	X	X	X	X	X
IVL	INVOLUCRIN	3713	-1.147025	0.0289809	-	X	X	X	X	X	X
CD38	CD38 ANTIGEN (P45)	952	-1.148147	0.0155044	-	X	X	X	X	X	X
IRS1	INSULIN RECEPTOR SUBSTRATE 1	3667	-1.208172	0.0111794	-	X	X	X	X	X	X
HES1	HAIRY AND ENHANCER OF SPLIT 1, (DROSOPHILA)	3280	-1.118693	0.0443647	-	X	X	X	X	X	X
LYN	V-YES-1 YAMAGUCHI SARCOMA VIRAL RELATED ONCOGENE HOMOLOG	4067	-1.126055	0.039063	-	X	X	X	X	X	X
EIF2AK2	EUKARYOTIC TRANSLATION INITIATION FACTOR 2-ALPHA KINASE 2	5610	-1.131951	0.0188806	-	X	X	X	X	X	X
F2R	COAGULATION FACTOR II (THROMBIN) RECEPTOR	2149	-1.157264	0.0122552	-	X	X	X	X	X	X
TIMP2	TIMP METALLOPEPTIDASE INHIBITOR 2	7077	-1.198009	0.0017053	-	X	X	X	X	X	X
LGALS3	LECTIN, GALACTOSIDE-BINDING, SOLUBLE, 3 (GALECTIN 3)	3958	-1.106458	0.0251063	-	X	X	X	X	X	X
NDRG1	N-MYC DOWNSTREAM REGULATED GENE 1	10397	-1.109282	0.042077	-	X	X	X	X	X	X
SYK	SPLEEN TYROSINE KINASE	6850	-1.127996	0.0175201	-	X	X	X	X	X	X
CD24	CD24 ANTIGEN (SMALL CELL LUNG CARCINOMA CLUSTER 4 ANTIGEN)	934	-1.129404	0.0260757	-	X	X	X	X	X	X
TCF4	TRANSCRIPTION FACTOR 4	6925	-1.14268	0.0346143	-	X	X	X	X	X	X
KRT5	KERATIN 4	3852	-1.182212	0.0158816	-	X	X	X	X	X	X
S100B	S100 CALCIUM BINDING PROTEIN, BETA (NEURAL)	6285	-1.192039	0.0154852	-	X	X	X	X	X	X
CCR5	CHEMOKINE (C-C MOTIF) RECEPTOR 5	1234	-1.160036	0.006676	-	X	X	X	X	X	-
IL18	INTERLEUKIN 18 (INTERFERON-GAMMA-INDUCING FACTOR)	3606	-1.236457	0.009133	-	X	X	X	X	X	-
UCP2	UNCOUPLING PROTEIN 2 (MITOCHONDRIAL, PROTON CARRIER)	7351	-1.118057	0.0216205	-	X	X	X	X	X	-
LY96	LYMPHOCYTE ANTIGEN 96	23643	-1.203485	0.0076904	-	X	X	X	X	X	-
PROCR	PROTEIN C RECEPTOR, ENDOTHELIAL (EPCR)	10544	-1.349698	0.005856	-	X	X	X	X	X	-
SOCS1	SUPPRESSOR OF CYTOKINE SIGNALING 1	8651	-1.138771	0.0064606	-	X	X	X	X	-	X
RAP1A	RAP1A, MEMBER OF RAS ONCOGENE FAMILY	5906	-1.118606	0.0414657	-	X	X	X	X	-	X
LCN2	LIPOCALIN 2 (ONCOGENE 24P3)	3934	-1.147608	0.0269466	-	X	X	X	X	-	X
TSC22D1	TSC22 DOMAIN FAMILY, MEMBER 1	8848	-1.120919	0.0167199	-	X	X	X	X	-	X
ENPP2	ECTONUCLEOTIDE PYROPHOSPHATASE/PHOSPHODIESTERASE 2 (AUTOTAXIN)	5168	-1.393675	0.0025781	-	X	X	X	-	X	X
GADD45G	GROWTH ARREST AND DNA-DAMAGE-INDUCIBLE, GAMMA	10912	-1.1592	0.0278591	-	X	X	X	X	-	-
INDO	INDOLEAMINE-PYRROLE 2,3 DIOXYGENASE	3620	-1.243826	0.0056476	-	X	X	X	X	-	-
HAS1	HYALURONAN SYNTHASE 1	3036	-1.114357	0.0352975	-	X	X	X	-	X	-
PIK3R1	PHOSPHOINOSITIDE-3-KINASE, REGULATORY SUBUNIT 1 (P85 ALPHA)	5295	-1.162636	0.0273528	-	X	X	X	-	-	X
RHOQ	RAS HOMOLOG GENE FAMILY, MEMBER Q	23433	-1.239093	0.0041901	-	X	X	X	-	-	X
S100A12	S100 CALCIUM BINDING PROTEIN A12 (CALGRANULIN C)	6283	-1.140022	0.0051456	-	X	X	-	X	-	X
NOG	NOGGIN	9241	-1.145907	0.0204701	-	X	X	-	X	-	X
HK2	HEXOKINASE 2	3099	-1.191321	0.0306796	-	X	-	X	X	X	-
PMP22	PERIPHERAL MYELIN PROTEIN 22	5376	-1.200327	0.0054017	-	X	-	X	X	-	X
CCL8	CHEMOKINE (C-C MOTIF) LIGAND 8	6355	-1.387893	0.00641	-	X	X	X	-	-	-
CLEC12A	C-TYPE LECTIN DOMAIN FAMILY 12, MEMBER A	160364	-1.160629	0.0463552	-	X	X	X	-	-	-
CCL13	CHEMOKINE (C-C MOTIF) LIGAND 13	6357	-1.231796	0.0020399	-	X	X	-	X	-	-
C3	COMPLEMENT COMPONENT 3	718	-1.136218	0.0167714	-	X	X	-	X	-	-
SMARCD3	SWI/SNF RELATED, MATRIX ASSOCIATED, ACTIN DEPENDENT REGULATOR OF CHROMATIN, SUBFAMILY D, MEMBER 3	6604	-1.145148	0.0132595	-	X	X	-	X	-	-
ALDH2	ALDEHYDE DEHYDROGENASE 2 FAMILY (MITOCHONDRIAL)	217	-1.139984	0.0397131	-	X	-	-	X	X	-

Gene Name	Gene Description	Entrez ID	Mean FC	*p*-value	PPRE (Lemay)	PPRE	NFkB	JUN	TP53	SP1	CTNNB1
UGDH	UDP-GLUCOSE DEHYDROGENASE	7358	-1.147466	0.0129344	-	X	-	-	-	X	-
CFD	COMPLEMENT FACTOR D (ADIPSIN)	1675	-1.12664	0.0376273	-	X	-	-	-	-	X
CRAT	CARNITINE ACETYLTRANSFERASE	1384	-1.110268	0.0451144	-	X	-	-	-	-	-
ALLC	ALLANTOICASE	55821	-1.117525	0.0274678	-	X	-	-	-	-	-
IGJ	IMMUNOGLOBULIN J POLYPEPTIDE, LINKER PROTEIN FOR IMMUNOGLOBULIN ALPHA AND MU POLYPEPTIDES	3512	-1.149491	0.0493812	-	X	-	-	-	-	-
AGPAT5	1-ACYLGLYCEROL-3-PHOSPHATE O-ACYLTRANSFERASE 5 (LYSOPHOSPHATIDIC ACID ACYLTRANSFERASE, EPSILON)	55326	-1.143501	0.0079372	-	X	-	-	-	-	-
NFKB2	NUCLEAR FACTOR OF KAPPA LIGHT POLYPEPTIDE GENE ENHANCER IN B-CELLS 2 (P49/P100)	4791	1.235595	0.0048016	-	-	X	X	X	X	X
FOSL2	FOS-LIKE ANTIGEN 2	2355	1.13692	0.0386156	-	-	X	X	X	X	X
NA	SERINE PI KAZAL TYPE 5-LIKE 3	153218	1.160847	0.0280238	-	-	X	X	X	X	X
EREG	EPIREGULIN	2069	1.551136	0.0032849	-	-	X	X	X	X	X
OGT	O-LINKED N-ACETYLGLUCOSAMINE (GLCNAC) TRANSFERASE (UDP-N-ACETYLGLUCOSAMINE:POLYPEPTIDE-N-ACETYLGLUCOSAMINYL TRANSFERASE)	8473	1.369261	0.0003444	-	-	X	X	X	X	X
SERPINB2	SERPIN PEPTIDASE INHIBITOR, CLADE B (OVALBUMIN), MEMBER 2	5055	1.319708	0.0305907	-	-	X	X	X	X	X
PLAUR	PLASMINOGEN ACTIVATOR, UROKINASE RECEPTOR	5329	1.266924	0.0042184	-	-	X	X	X	X	X
ATM	ATAXIA TELANGIECTASIA MUTATED (INCLUDES COMPLEMENTATION GROUPS A, C AND D)	472	1.206358	0.0321543	-	-	X	X	X	X	X
SLC3A2	SOLUTE CARRIER FAMILY 3 (ACTIVATORS OF DIBASIC AND NEUTRAL AMINO ACID TRANSPORT), MEMBER 2	6520	1.154967	0.0074982	-	-	X	X	X	X	X
TSC2	TUBEROUS SCLEROSIS 2	7249	1.145612	0.0086829	-	-	X	X	X	X	X
BCS1L	BCS1-LIKE (YEAST)	617	1.098568	0.0333298	-	-	X	X	X	X	X
CCL20	CHEMOKINE (C-C MOTIF) LIGAND 20	6364	1.213736	0.0247419	-	-	X	X	X	X	-
MAT2A	METHIONINE ADENOSYLTRANSFERASE II, ALPHA	4144	1.369628	0.0146805	-	-	X	X	X	X	-
KLRC4	KILLER CELL LECTIN-LIKE RECEPTOR SUBFAMILY C, MEMBER 4	22914	1.211918	0.0139737	-	-	X	X	X	X	-
PLD2	PHOSPHOLIPASE D2	5338	1.202441	0.0037298	-	-	X	X	X	X	-
MC1R	MELANOCORTIN 1 RECEPTOR (ALPHA MELANOCYTE STIMULATING HORMONE RECEPTOR)	4157	1.191449	0.0489429	-	-	X	X	X	X	-
ARAF	V-RAF MURINE SARCOMA 3611 VIRAL ONCOGENE HOMOLOG	369	1.150499	0.0048809	-	-	X	X	X	X	-
BTG2	BTG FAMILY, MEMBER 2	7832	1.148186	0.0276721	-	-	X	X	X	X	-
PLA2G6	PHOSPHOLIPASE A2, GROUP VI (CYTOSOLIC, CALCIUM-INDEPENDENT)	8398	1.139754	0.032901	-	-	X	X	X	X	-
CDC37	CDC37 CELL DIVISION CYCLE 37 HOMOLOG (S. CEREVISIAE)	11140	1.122661	0.0305307	-	-	X	X	X	X	-
UCN	UROCORTIN	7349	1.107027	0.0342558	-	-	X	X	X	X	-
ZAP70	ZETA-CHAIN (TCR) ASSOCIATED PROTEIN KINASE 70KDA	7535	1.122268	0.0111245	-	-	X	X	X	-	X
MDM4	MDM4, TRANSFORMED 3T3 CELL DOUBLE MINUTE 4, P53 BINDING PROTEIN (MOUSE)	4194	1.214216	0.0133913	-	-	X	X	X	-	X
APLP2	AMYLOID BETA (A4) PRECURSOR-LIKE PROTEIN 2	334	1.142955	0.0067595	-	-	X	X	X	-	X
HDAC6	HISTONE DEACETYLASE 6	10013	1.133876	0.0203317	-	-	X	X	X	-	X
SFRS2	SPLICING FACTOR, ARGININE/SERINE-RICH 2	6427	1.14994	0.0390999	-	-	X	X	X	-	-
PHLDA1	PLECKSTRIN HOMOLOGY-LIKE DOMAIN, FAMILY A, MEMBER 1	22822	1.377449	0.0008819	-	-	X	X	X	-	-
DHX9	DEAH (ASP-GLU-ALA-HIS) BOX POLYPEPTIDE 9	1660	1.335025	0.0169281	-	-	X	X	X	-	-
PIK3C2A	PHOSPHOINOSITIDE-3-KINASE, CLASS 2, ALPHA POLYPEPTIDE	5286	1.225092	0.0100972	-	-	X	X	X	-	-
TBC1D4	TBC1 DOMAIN FAMILY, MEMBER 4	9882	1.172202	0.0228672	-	-	X	X	X	-	-
DUSP5	DUAL SPECIFICITY PHOSPHATASE 5	1847	1.171809	0.0087639	-	-	X	X	X	-	-
RAB6A	RAB6A, MEMBER RAS ONCOGENE FAMILY	5870	1.165734	0.0335133	-	-	X	X	X	-	-
LYST	LYSOSOMAL TRAFFICKING REGULATOR	1130	1.133111	0.0460005	-	-	X	X	X	-	-
TNFRSF21	TUMOR NECROSIS FACTOR RECEPTOR SUPERFAMILY, MEMBER 21	27242	1.125742	0.0439119	-	-	X	X	X	-	-
PSG5	PREGNANCY SPECIFIC BETA-1-GLYCOPROTEIN 5	5673	1.203441	0.0162549	-	-	X	X	-	X	X
ATP8A1	ATPASE, AMINOPHOSPHOLIPID TRANSPORTER (APLT), CLASS I, TYPE 8A, MEMBER 1	10396	1.267505	0.0197789	-	-	X	X	-	X	-
FCGRT	FC FRAGMENT OF IGG, RECEPTOR, TRANSPORTER, ALPHA	2217	1.162608	0.0068935	-	-	X	X	-	X	-
MZF1	ZINC FINGER PROTEIN 42 (MYELOID-SPECIFIC RETINOIC ACID-RESPONSIVE)	7593	1.136398	0.013784	-	-	X	X	-	X	-
HARS2	HISTIDYL-TRNA SYNTHETASE-LIKE	23438	1.124307	0.0261991	-	-	X	X	-	X	-
PSCD1	PLECKSTRIN HOMOLOGY, SEC7 AND COILED-COIL DOMAINS 1(CYTOHESIN 1)	9267	1.114257	0.0471591	-	-	X	X	-	X	-
STX2	EPIMORPHIN	2054	1.183928	0.0113964	-	-	X	X	-	-	X
TLR6	TOLL-LIKE RECEPTOR 6	10333	1.246636	0.0055319	-	-	X	X	-	-	-
MAP3K8	MITOGEN-ACTIVATED PROTEIN KINASE KINASE KINASE 8	1326	1.602521	0.000235	-	-	X	X	-	-	-
FCAR	FC FRAGMENT OF IGA, RECEPTOR FOR	2204	1.568706	0.0183386	-	-	X	X	-	-	-
CXCL3	CHEMOKINE (C-X-C MOTIF) LIGAND 3	2921	1.459202	0.0022673	-	-	X	X	-	-	-
PDE7A	PHOSPHODIESTERASE 7A	5150	1.314759	0.0009109	-	-	X	X	-	-	-
NPEPL1	SYNTAXIN 16	8675	1.257295	0.0027588	-	-	X	X	-	-	-
ATXN7	ATAXIN 7	6314	1.255968	0.0313858	-	-	X	X	-	-	-
KLRG1	KILLER CELL LECTIN-LIKE RECEPTOR SUBFAMILY G, MEMBER 1	10219	1.237819	0.0029266	-	-	X	X	-	-	-
TXK	TXK TYROSINE KINASE	7294	1.190536	0.0410083	-	-	X	X	-	-	-
AGER	ADVANCED GLYCOSYLATION END PRODUCT-SPECIFIC RECEPTOR	177	1.11053	0.0421925	-	-	X	X	-	-	-
TRPC4AP	TRANSIENT RECEPTOR POTENTIAL CATION CHANNEL, SUBFAMILY C, MEMBER 4 ASSOCIATED PROTEIN	26133	1.096761	0.0298432	-	-	X	X	-	-	-
ITK	IL2-INDUCIBLE T-CELL KINASE	3702	1.091007	0.0434902	-	-	X	X	-	-	-
HPSE	HEPARANASE	10855	1.121663	0.0371457	-	-	X	-	X	X	X
DDIT4	DNA-DAMAGE-INDUCIBLE TRANSCRIPT 4	54541	1.160405	0.0152844	-	-	X	-	X	X	X
NP	NUCLEOSIDE PHOSPHORYLASE	4860	1.160813	0.0230622	-	-	X	-	X	X	-
SLC25A1	SOLUTE CARRIER FAMILY 25 (MITOCHONDRIAL CARRIER; CITRATE TRANSPORTER), MEMBER 1	6576	1.139478	0.0161011	-	-	X	-	X	X	-
FAIM3	FAS APOPTOTIC INHIBITORY MOLECULE 3	9214	1.130872	0.0214335	-	-	X	-	X	-	X
DLG1	DISCS, LARGE HOMOLOG 1 (DROSOPHILA)	1739	1.361418	0.0122446	-	-	X	-	X	-	X
RASSF5	RAS ASSOCIATION (RALGDS/AF-6) DOMAIN FAMILY 5	83593	1.129578	0.0465051	-	-	X	-	X	-	X
CDK5RAP3	CDK5 REGULATORY SUBUNIT ASSOCIATED PROTEIN 3	80279	1.237733	0.0014025	-	-	X	-	X	-	-
NLRC4	CASPASE RECRUITMENT DOMAIN FAMILY, MEMBER 12	58484	1.355603	0.0003146	-	-	X	-	X	-	-
TIA1	TIA1 CYTOTOXIC GRANULE-ASSOCIATED RNA BINDING PROTEIN	7072	1.263602	0.021618	-	-	X	-	X	-	-
PPBP	PRO-PLATELET BASIC PROTEIN (CHEMOKINE (C-X-C MOTIF) LIGAND 7)	5473	1.247852	0.0196399	-	-	X	-	X	-	-
DEADC1	DEAMINASE DOMAIN CONTAINING 1	134637	1.203338	0.0005777	-	-	X	-	X	-	-
UPP1	URIDINE PHOSPHORYLASE 1	7378	1.139971	0.013681	-	-	X	-	X	-	-
BTG3	BTG FAMILY, MEMBER 3	10950	1.134087	0.0462874	-	-	X	-	X	-	-
MX2	MYXOVIRUS (INFLUENZA VIRUS) RESISTANCE 2 (MOUSE)	4600	1.090936	0.0477149	-	-	X	-	X	-	-
GALNS	GALACTOSAMINE (N-ACETYL)-6-SULFATE SULFATASE (MORQUIO SYNDROME, MUCOPOLYSACCHARIDOSIS TYPE IVA)	2588	1.105897	0.0236463	-	-	X	-	-	X	X
P2RX4	PURINERGIC RECEPTOR P2X, LIGAND-GATED ION CHANNEL, 4	5025	1.221286	0.0018855	-	-	X	-	-	X	-
ROCK2	RHO-ASSOCIATED, COILED-COIL CONTAINING PROTEIN KINASE 2	9475	1.242963	0.0007042	-	-	X	-	-	-	X
DVL3	DISHEVELLED, DSH HOMOLOG 3 (DROSOPHILA)	1857	1.153175	0.0047967	-	-	X	-	-	-	X
IHPK2	DKFZP586M0617 PROTEIN	51447	1.106448	0.0425998	-	-	X	-	-	-	X
CCR3	CHEMOKINE (C-C MOTIF) RECEPTOR 3	1232	1.265277	0.0446316	-	-	X	-	-	-	-
TTRAP	TRAF AND TNF RECEPTOR ASSOCIATED PROTEIN	51567	1.226402	0.0011483	-	-	X	-	-	-	-
DGKA	DIACYLGLYCEROL KINASE, ALPHA 80KDA	1606	1.34632	0.0039158	-	-	X	-	-	-	-
TPST1	TYROSYLPROTEIN SULFOTRANSFERASE 1	8460	1.328188	0.0015022	-	-	X	-	-	-	-
CCL24	CHEMOKINE (C-C MOTIF) LIGAND 24	6369	1.325575	0.0153003	-	-	X	-	-	-	-
NLRP1	NACHT, LEUCINE RICH REPEAT AND PYD (PYRIN DOMAIN) CONTAINING 1	22861	1.324044	0.0003859	-	-	X	-	-	-	-
ARL6IP2	ADP-RIBOSYLATION FACTOR-LIKE 6 INTERACTING PROTEIN 2	64225	1.253964	0.0027367	-	-	X	-	-	-	-
AQP9	AQUAPORIN 9	366	1.252194	0.0303952	-	-	X	-	-	-	-
GTF3C3	GENERAL TRANSCRIPTION FACTOR IIIC, POLYPEPTIDE 3, 102KDA	9330	1.207779	0.0157839	-	-	X	-	-	-	-
FCRL3	FC RECEPTOR-LIKE 3	115352	1.187554	0.0059376	-	-	X	-	-	-	-
PRPF3	PRP3 PRE-MRNA PROCESSING FACTOR 3 HOMOLOG (YEAST)	9129	1.182113	0.0018531	-	-	X	-	-	-	-
SYTL1	SYNAPTOTAGMIN-LIKE 1	84958	1.171324	0.0017504	-	-	X	-	-	-	-
LPXN	LEUPAXIN	9404	1.171053	0.0143063	-	-	X	-	-	-	-
THEM2	THIOESTERASE SUPERFAMILY MEMBER 2	55856	1.159261	0.02806	-	-	X	-	-	-	-
ZCCHC11	ZINC FINGER, CCHC DOMAIN CONTAINING 11	23318	1.158596	0.0130283	-	-	X	-	-	-	-
ACBD5	ACYL-COENZYME A BINDING DOMAIN CONTAINING 5	91452	1.158351	0.0159518	-	-	X	-	-	-	-
IL18RAP	INTERLEUKIN 18 RECEPTOR ACCESSORY PROTEIN	8807	1.15739	0.0310144	-	-	X	-	-	-	-
NOLC1	NUCLEOLAR AND COILED-BODY PHOSPHOPROTEIN 1	9221	1.139717	0.0442955	-	-	X	-	-	-	-
MTMR11	MYOTUBULARIN RELATED PROTEIN 11	10903	1.120994	0.0385757	-	-	X	-	-	-	-
NPLOC4	NUCLEAR PROTEIN LOCALIZATION 4 HOMOLOG (S. CEREVISIAE)	55666	1.118979	0.0177439	-	-	X	-	-	-	-
GOLGA1	GOLGI AUTOANTIGEN, GOLGIN SUBFAMILY A, 1	2800	1.11837	0.0477539	-	-	X	-	-	-	-
FKRP	FUKUTIN RELATED PROTEIN	79147	1.117538	0.0315691	-	-	X	-	-	-	-
SEC61A1	SEC61 ALPHA 1 SUBUNIT (S. CEREVISIAE)	29927	1.110923	0.0260134	-	-	X	-	-	-	-
ODC1	ORNITHINE DECARBOXYLASE 1	4953	1.109804	0.0456792	-	-	X	-	-	-	-
	ARS2 PROTEIN	51593	1.10558	0.0427812	-	-	X	-	-	-	-
USP21	UBIQUITIN SPECIFIC PEPTIDASE 21	27005	1.091007	0.0389259	-	-	X	-	-	-	-
ST14	SUPPRESSION OF TUMORIGENICITY 14 (COLON CARCINOMA)	6768	1.59697	2.84E-05	-	-	-	X	X	X	X
MGEA5	MENINGIOMA EXPRESSED ANTIGEN 5 (HYALURONIDASE)	10724	1.246093	0.0020754	-	-	-	X	X	X	X
ASNS	ASPARAGINE SYNTHETASE	440	1.217094	0.0120274	-	-	-	X	X	X	-
ENO3	ENOLASE 1, (ALPHA)	2027	1.145273	0.0308203	-	-	-	X	X	X	-

Gene Name	Gene Description	Entrez ID	Mean FC	*p*-value	PPRE (Lemay)	PPRE	NFkB	JUN	TP53	SP1	CTNNB1
NUP214	NUCLEOPORIN 214KDA	8021	1.343981	0.0001471	-	-	-	X	X	-	X
		728642	1.285525	0.0009337	-	-	-	X	X	-	-
TAGLN	TRANSGELIN	6876	1.213331	0.0067784	-	-	-	X	X	-	-
TMOD4	TROPOMODULIN 4 (MUSCLE)	29765	1.209879	0.0140845	-	-	-	X	X	-	-
BTN2A1	BUTYROPHILIN, SUBFAMILY 2, MEMBER A1	11120	1.182641	0.0102522	-	-	-	X	X	-	-
C13orf15	RESPONSE GENE TO COMPLEMENT 32	28984	1.13443	0.0164725	-	-	-	X	X	-	-
CDK10	CYCLIN-DEPENDENT KINASE (CDC2-LIKE) 10	8558	1.103178	0.0335485	-	-	-	X	X	-	-
MAFG	V-MAF MUSCULOAPONEUROTIC FIBROSARCOMA ONCOGENE HOMOLOG G (AVIAN)	4097	1.102583	0.0383607	-	-	-	X	X	-	-
PCYT1A	PHOSPHATE CYTIDYLYLTRANSFERASE 1, CHOLINE, ALPHA	5130	1.176331	0.0462311	-	-	-	X	-	X	-
DHRS9	DEHYDROGENASE/REDUCTASE (SDR FAMILY) MEMBER 9	10170	1.353644	0.0038646	-	-	-	X	-	-	X
RAPGEF1	RAP GUANINE NUCLEOTIDE EXCHANGE FACTOR (GEF) 1	2889	1.316	0.0121788	-	-	-	X	-	-	X
ARFGEF2	ADP-RIBOSYLATION FACTOR GUANINE NUCLEOTIDE-EXCHANGE FACTOR 2 (BREFELDIN A-INHIBITED)	10564	1.145501	0.0246067	-	-	-	X	-	-	X
KIAA1509	KIAA1509	440193	1.130306	0.0342305	-	-	-	X	-	-	X
DNAJA4	DNAJ (HSP40) HOMOLOG, SUBFAMILY A, MEMBER 4	55466	1.213101	0.0042369	-	-	-	X	-	-	-
CXorf15	CHROMOSOME X OPEN READING FRAME 15	55787	1.212336	0.0066309	-	-	-	X	-	-	-
GGA3	GOLGI ASSOCIATED, GAMMA ADAPTIN EAR CONTAINING, ARF BINDING PROTEIN 3	23163	1.193256	0.0125325	-	-	-	X	-	-	-
GGA2	GOLGI ASSOCIATED, GAMMA ADAPTIN EAR CONTAINING, ARF BINDING PROTEIN 2	23062	1.179916	0.0024164	-	-	-	X	-	-	-
GGA1	GOLGI ASSOCIATED, GAMMA ADAPTIN EAR CONTAINING, ARF BINDING PROTEIN 1	26088	1.172256	0.0236234	-	-	-	X	-	-	-
ATE1	ARGINYLTRANSFERASE 1	11101	1.165327	0.0040994	-	-	-	X	-	-	-
RBM14	RNA BINDING MOTIF PROTEIN 14	10432	1.15496	0.0038852	-	-	-	X	-	-	-
TMC6	TRANSMEMBRANE CHANNEL-LIKE 6	11322	1.144897	0.004527	-	-	-	X	-	-	-
MCCC2	METHYLCROTONOYL-COENZYME A CARBOXYLASE 2 (BETA)	64087	1.142413	0.0251142	-	-	-	X	-	-	-
UBR2	UBIQUITIN PROTEIN LIGASE E3 COMPONENT N-RECOGNIN 2	23304	1.142168	0.0070213	-	-	-	X	-	-	-
MKNK1	MAP KINASE INTERACTING SERINE/THREONINE KINASE 1	8569	1.139561	0.0311508	-	-	-	X	-	-	-
SCYL2	SCY1-LIKE 2 (S. CEREVISIAE)	55681	1.139115	0.0372344	-	-	-	X	-	-	-
TNNI3	TROPONIN I TYPE 3 (CARDIAC)	7137	1.138429	0.0316155	-	-	-	X	-	-	-
RBM39	RNA-BINDING REGION (RNP1, RRM) CONTAINING 2	9584	1.138109	0.0269826	-	-	-	X	-	-	-
ZNF589	ZINC FINGER PROTEIN 589	51385	1.138033	0.0175473	-	-	-	X	-	-	-
MAFF	V-MAF MUSCULOAPONEUROTIC FIBROSARCOMA ONCOGENE HOMOLOG F (AVIAN)	23764	1.135014	0.0385719	-	-	-	X	-	-	-
PHKA2	PHOSPHORYLASE KINASE, ALPHA 2 (LIVER)	5256	1.1348	0.0146996	-	-	-	X	-	-	-
STMN3	STATHMIN-LIKE 3	50861	1.131419	0.0146115	-	-	-	X	-	-	-
CABP2	CALCIUM BINDING PROTEIN 2	51475	1.129444	0.0152903	-	-	-	X	-	-	-
PTPN7	PROTEIN TYROSINE PHOSPHATASE, NON-RECEPTOR TYPE 7	5778	1.127773	0.037278	-	-	-	X	-	-	-
AP4M1	ADAPTOR-RELATED PROTEIN COMPLEX 4, MU 1 SUBUNIT	9179	1.119404	0.0383511	-	-	-	X	-	-	-
TCIRG1	T-CELL, IMMUNE REGULATOR 1, ATPASE, H+ TRANSPORTING, LYSOSOMAL V0 SUBUNIT A3	10312	1.115942	0.0314652	-	-	-	X	-	-	-
ARFIP2	ADP-RIBOSYLATION FACTOR INTERACTING PROTEIN 2 (ARFAPTIN 2)	23647	1.115156	0.0152347	-	-	-	X	-	-	-
RUTBC3	RUN AND TBC1 DOMAIN CONTAINING 3	27352	1.10888	0.0449784	-	-	-	X	-	-	-
MTA1	METASTASIS ASSOCIATED 1	9112	1.126892	0.0147657	-	-	-	-	X	X	X
ZNF76	ZINC FINGER PROTEIN 76 (EXPRESSED IN TESTIS)	7629	1.164331	0.0021687	-	-	-	-	X	X	-
KIF22	KINESIN FAMILY MEMBER 22	3835	1.154986	0.0098753	-	-	-	-	X	X	-
HOXB2	HOMEOBOX B2	3212	1.152062	0.0201916	-	-	-	-	X	X	-
TPP2	TRIPEPTIDYL PEPTIDASE II	7174	1.151212	0.0044215	-	-	-	-	X	X	-
ESPL1	EXTRA SPINDLE POLES LIKE 1 (S. CEREVISIAE)	9700	1.150667	0.0432862	-	-	-	-	X	X	-
SYVN1	SYNOVIAL APOPTOSIS INHIBITOR 1, SYNOVIOLIN	84447	1.142177	0.0122984	-	-	-	-	X	X	-
MBD1	METHYL-CPG BINDING DOMAIN PROTEIN 1	4152	1.118557	0.0369778	-	-	-	-	X	X	-
PNN	PININ, DESMOSOME ASSOCIATED PROTEIN	5411	1.318845	0.0016569	-	-	-	-	X	-	X
STAB1	STABILIN 1	23166	1.259015	0.0019828	-	-	-	-	X	-	X
MED12	MEDIATOR OF RNA POLYMERASE II TRANSCRIPTION, SUBUNIT 12 HOMOLOG (YEAST)	9968	1.128224	0.0378068	-	-	-	-	X	-	X
MTCH1	MITOCHONDRIAL CARRIER HOMOLOG 1 (C. ELEGANS)	23787	1.118842	0.0374181	-	-	-	-	X	-	X
FMNL1	FORMIN-LIKE 1	752	1.108594	0.0497026	-	-	-	-	X	-	X
TNCRNA	TROPHOBLAST-DERIVED NONCODING RNA	283131	2.07056	0.0072429	-	-	-	-	X	-	-
CCNL1	CYCLIN L1	57018	1.411922	0.0015001	-	-	-	-	X	-	-
JARID1D	SMCY HOMOLOG, Y-LINKED (MOUSE)	8284	1.337343	0.0009864	-	-	-	-	X	-	-
TBRG1	TRANSFORMING GROWTH FACTOR BETA REGULATOR 1	84897	1.291541	0.0029275	-	-	-	-	X	-	-
FNBP4	FORMIN BINDING PROTEIN 4	23360	1.288743	0.0003825	-	-	-	-	X	-	-
POLI	POLYMERASE (DNA DIRECTED) IOTA	11201	1.250385	0.010098	-	-	-	-	X	-	-
JARID2	JUMONJI, AT RICH INTERACTIVE DOMAIN 2	3720	1.24979	0.0017626	-	-	-	-	X	-	-
ABCC5	ATP-BINDING CASSETTE, SUB-FAMILY C (CFTR/MRP), MEMBER 5	10057	1.237836	0.0009969	-	-	-	-	X	-	-
CHKB	CHOLINE KINASE BETA	1120	1.236901	0.0012345	-	-	-	-	X	-	-
CCNL2	CYCLIN L2	81669	1.236433	0.001746	-	-	-	-	X	-	-
ECH1	ENOYL COENZYME A HYDRATASE 1, PEROXISOMAL	1891	1.230146	0.0013774	-	-	-	-	X	-	-
LGALS8	LECTIN, GALACTOSIDE-BINDING, SOLUBLE, 8 (GALECTIN 8)	3964	1.217587	0.0380604	-	-	-	-	X	-	-
SFRS5	SPLICING FACTOR, ARGININE/SERINE-RICH 5	6430	1.216697	0.0133567	-	-	-	-	X	-	-
MS4A1	MEMBRANE-SPANNING 4-DOMAINS, SUBFAMILY A, MEMBER 1	931	1.207699	0.0260652	-	-	-	-	X	-	-
MAN2C1	MANNOSIDASE, ALPHA, CLASS 2C, MEMBER 1	4123	1.204503	0.0087098	-	-	-	-	X	-	-
RBM5	RNA BINDING MOTIF PROTEIN 5	10181	1.191709	0.0027828	-	-	-	-	X	-	-
CLK2	CDC-LIKE KINASE 2	1196	1.186027	0.0015156	-	-	-	-	X	-	-
LIG1	LIGASE I, DNA, ATP-DEPENDENT	3978	1.172517	0.0039648	-	-	-	-	X	-	-
NONO	NON-POU DOMAIN CONTAINING, OCTAMER-BINDING	4841	1.163635	0.0151507	-	-	-	-	X	-	-
GOLGB1	GOLGI AUTOANTIGEN, GOLGIN SUBFAMILY B, MACROGOLGIN (WITH TRANSMEMBRANE SIGNAL), 1	2804	1.157957	0.0208498	-	-	-	-	X	-	-
TRIM25	TRIPARTITE MOTIF-CONTAINING 25	7706	1.155234	0.0226342	-	-	-	-	X	-	-
BTAF1	BTAF1 RNA POLYMERASE II, B-TFIID TRANSCRIPTION FACTOR-ASSOCIATED, 170KDA (MOT1 HOMOLOG, S. CEREVISIAE)	9044	1.152126	0.0121452	-	-	-	-	X	-	-
SCO2	SCO CYTOCHROME OXIDASE DEFICIENT HOMOLOG 2 (YEAST)	9997	1.145281	0.0255324	-	-	-	-	X	-	-
AK1	ADENYLATE KINASE 1	203	1.130693	0.0264247	-	-	-	-	X	-	-
NUP107	NUCLEOPORIN 107KDA	57122	1.130611	0.0223903	-	-	-	-	X	-	-
NA	VPR (HIV-1) BINDING PROTEIN	9730	1.129708	0.0347139	-	-	-	-	X	-	-
LETMD1	LETM1 DOMAIN CONTAINING 1	25875	1.120216	0.0248971	-	-	-	-	X	-	-
DMAP1	DNA METHYLTRANSFERASE 1 ASSOCIATED PROTEIN 1	55929	1.115771	0.0397448	-	-	-	-	X	-	-
MTSS1	METASTASIS SUPPRESSOR 1	9788	1.108461	0.0297534	-	-	-	-	X	-	-
NAP1L4	NUCLEOSOME ASSEMBLY PROTEIN 1-LIKE 4	4676	1.105934	0.0281513	-	-	-	-	X	-	-
HSPH1	HEAT SHOCK 105KDA/110KDA PROTEIN 1	10808	1.098787	0.0363109	-	-	-	-	X	-	-
REV1	REV1-LIKE (YEAST)	51455	1.09799	0.0492732	-	-	-	-	X	-	-
WHSC1	WOLF-HIRSCHHORN SYNDROME CANDIDATE 1	7468	1.086327	0.0443893	-	-	-	-	X	-	-
COPA	COATOMER PROTEIN COMPLEX, SUBUNIT ALPHA	1314	1.166114	0.0311392	-	-	-	-	-	X	X
IMPA2	INOSITOL(MYO)-1(OR 4)-MONOPHOSPHATASE 2	3613	1.465573	1.01E-05	-	-	-	-	-	X	-
P2RX1	PURINERGIC RECEPTOR P2X, LIGAND-GATED ION CHANNEL, 1	5023	1.213791	0.0065381	-	-	-	-	-	X	-
HPD	4-HYDROXYPHENYLPYRUVATE DIOXYGENASE	3242	1.176786	0.0064513	-	-	-	-	-	X	-
LCP1	LYMPHOCYTE CYTOSOLIC PROTEIN 1 (L-PLASTIN)	3936	1.162391	0.0325606	-	-	-	-	-	X	-
DDX51	DEAD (ASP-GLU-ALA-ASP) BOX POLYPEPTIDE 51	317781	1.156671	0.0211543	-	-	-	-	-	X	-
ITGAE	INTEGRIN, ALPHA E (ANTIGEN CD103, HUMAN MUCOSAL LYMPHOCYTE ANTIGEN 1; ALPHA POLYPEPTIDE)	3682	1.135062	0.0095314	-	-	-	-	-	X	-
TLE4	TRANSDUCIN-LIKE ENHANCER OF SPLIT 4 (E(SP1) HOMOLOG, DROSOPHILA)	7091	1.240594	0.0062951	-	-	-	-	-	-	X
HGS	HEPATOCYTE GROWTH FACTOR-REGULATED TYROSINE KINASE SUBSTRATE	9146	1.214905	0.0020026	-	-	-	-	-	-	X
HAND2	HEART AND NEURAL CREST DERIVATIVES EXPRESSED 2	9464	1.203039	0.0413883	-	-	-	-	-	-	X
RASGRP2	RAS GUANYL RELEASING PROTEIN 2 (CALCIUM AND DAG-REGULATED)	10235	1.181577	0.004454	-	-	-	-	-	-	X
PLEC1	PLECTIN 1, INTERMEDIATE FILAMENT BINDING PROTEIN 500KDA	5339	1.176561	0.0020615	-	-	-	-	-	-	X
ARIH1	ARIADNE HOMOLOG	25820	1.138315	0.0431209	-	-	-	-	-	-	X
EVL	ENAH/VASP-LIKE	51466	1.134599	0.0111857	-	-	-	-	-	-	X
SH3BGRL3	SH3 DOMAIN BINDING GLUTAMIC ACID-RICH PROTEIN LIKE 3	83442	1.117533	0.0153052	-	-	-	-	-	-	X
OFD1	ORAL-FACIAL-DIGITAL SYNDROME 1	8481	1.115716	0.0306719	-	-	-	-	-	-	X
WDR42A	WD REPEAT DOMAIN 42A	50717	1.107688	0.0287361	-	-	-	-	-	-	X
TNFRSF8	TUMOR NECROSIS FACTOR RECEPTOR SUPERFAMILY, MEMBER 8	943	-1.18259	0.0023737	-	-	X	X	X	X	X
S100A4	S100 CALCIUM BINDING PROTEIN A4 (CALCIUM PROTEIN, CALVASCULIN, METASTASIN, MURINE PLACENTAL HOMOLOG)	6275	-1.252421	0.0006637	-	-	X	X	X	X	X
ADA	ADENOSINE DEAMINASE	100	-1.101698	0.0286091	-	-	X	X	X	X	X
HDAC4	HISTONE DEACETYLASE 4	9759	-1.106653	0.0376649	-	-	X	X	X	X	X
PLAT	PLASMINOGEN ACTIVATOR, TISSUE	5327	-1.110631	0.04906	-	-	X	X	X	X	X
FES	FELINE SARCOMA ONCOGENE	2242	-1.121785	0.0410044	-	-	X	X	X	X	X
S100A6	S100 CALCIUM BINDING PROTEIN A6 (CALCYCLIN)	6277	-1.145191	0.0056957	-	-	X	X	X	X	X
MARCKS	MYRISTOYLATED ALANINE-RICH PROTEIN KINASE C SUBSTRATE	4082	-1.145285	0.0175691	-	-	X	X	X	X	X
RRM2	RIBONUCLEOTIDE REDUCTASE M2 POLYPEPTIDE	6241	-1.145421	0.0384581	-	-	X	X	X	X	X
LYZ	LYSOZYME (RENAL AMYLOIDOSIS)	4069	-1.165296	0.0181383	-	-	X	X	X	X	X

Gene Name	Gene Description	Entrez ID	Mean FC	*p*-value	PPRE (Lemay)	PPRE	NFkB	JUN	TP53	SP1	CTNNB1
PTMA	PROTHYMOSIN, ALPHA (GENE SEQUENCE 28)	5757	-1.179494	0.0279334	-	-	X	X	X	X	X
QPCT	GLUTAMINYL-PEPTIDE CYCLOTRANSFERASE (GLUTAMINYL CYCLASE)	25797	-1.198046	0.0044182	-	-	X	X	X	X	X
GJB2	GAP JUNCTION PROTEIN, BETA 2, 26KDA (CONNEXIN 26)	2706	-1.224013	0.0225943	-	-	X	X	X	X	X
IFI6	INTERFERON, ALPHA-INDUCIBLE PROTEIN (CLONE IFI-6-16)	2537	-1.317363	0.0003046	-	-	X	X	X	X	X
UGCG	UDP-GLUCOSE CERAMIDE GLUCOSYLTRANSFERASE	7357	-1.20814	0.012798	-	-	X	X	X	X	-
GZMB	GRANZYME B (GRANZYME 2, CYTOTOXIC T-LYMPHOCYTE-ASSOCIATED SERINE ESTERASE 1)	3002	-1.096857	0.032401	-	-	X	X	X	X	-
PLD1	PHOSPHOLIPASE D1, PHOSPHATIDYLCHOLINE-SPECIFIC	5337	-1.111004	0.0211089	-	-	X	X	X	X	-
ABP1	AMILORIDE BINDING PROTEIN 1 (AMINE OXIDASE (COPPER-CONTAINING))	26	-1.113615	0.0190214	-	-	X	X	X	X	-
DPYD	DIHYDROPYRIMIDINE DEHYDROGENASE	1806	-1.13849	0.0374125	-	-	X	X	X	X	-
CD33	CD33 ANTIGEN (GP67)	945	-1.156924	0.0151161	-	-	X	X	X	X	-
IGFBP7	INSULIN-LIKE GROWTH FACTOR BINDING PROTEIN 7	3490	-1.178792	0.0206573	-	-	X	X	X	X	-
LMO2	LIM DOMAIN ONLY 2 (RHOMBOTIN-LIKE 1)	4005	-1.200344	0.0024876	-	-	X	X	X	X	-
A2M	ALPHA-2-MACROGLOBULIN	2	-1.23145	0.0133353	-	-	X	X	X	X	-
SNRPE	SMALL NUCLEAR RIBONUCLEOPROTEIN POLYPEPTIDE E	6635	-1.108765	0.0306235	-	-	X	X	X	-	X
PEBP1	PHOSPHATIDYLETHANOLAMINE BINDING PROTEIN 1	5037	-1.109728	0.0440823	-	-	X	X	X	-	X
NUDT1	NUDIX (NUCLEOSIDE DIPHOSPHATE LINKED MOIETY X)-TYPE MOTIF 1	4521	-1.118962	0.0333523	-	-	X	X	X	-	X
DAB2	DISABLED HOMOLOG 2, MITOGEN-RESPONSIVE PHOSPHOPROTEIN (DROSOPHILA)	1601	-1.137875	0.0084613	-	-	X	X	X	-	X
P2RX7	PURINERGIC RECEPTOR P2X, LIGAND-GATED ION CHANNEL, 7	5027	-1.104888	0.0434914	-	-	X	X	X	-	-
PRDX2	PEROXIREDOXIN 2	7001	-1.135138	0.039388	-	-	X	X	X	-	-
ITPK1	INOSITOL 1,3,4-TRIPHOSPHATE 5/6 KINASE	3705	-1.106791	0.0292959	-	-	X	X	X	-	-
PTPN2	PROTEIN TYROSINE PHOSPHATASE, NON-RECEPTOR TYPE 2	5771	-1.108902	0.0494007	-	-	X	X	X	-	-
S100A9	S100 CALCIUM BINDING PROTEIN A9 (CALGRANULIN B)	6280	-1.121712	0.0108515	-	-	X	X	X	-	-
PRDX4	PEROXIREDOXIN 4	10549	-1.152294	0.0310292	-	-	X	X	X	-	-
CD47	CD47 ANTIGEN (RH-RELATED ANTIGEN, INTEGRIN-ASSOCIATED SIGNAL TRANSDUCER)	961	-1.158915	0.0094536	-	-	X	X	X	-	-
RBM3	RNA BINDING MOTIF (RNP1, RRM) PROTEIN 3	5935	-1.168868	0.012362	-	-	X	X	X	-	-
PF4	PLATELET FACTOR 4 (CHEMOKINE (C-X-C MOTIF) LIGAND 4)	5196	-1.172978	0.0105529	-	-	X	X	X	-	-
F7	COAGULATION FACTOR VII (SERUM PROTHROMBIN CONVERSION ACCELERATOR)	2155	-1.092473	0.0476932	-	-	X	X	-	X	-
STOM	STOMATIN	2040	-1.098089	0.0419832	-	-	X	X	-	X	-
FGL2	FIBRINOGEN-LIKE 2	10875	-1.132791	0.0362513	-	-	X	X	-	X	-
CCR6	CHEMOKINE (C-C MOTIF) RECEPTOR 6	1235	-1.134726	0.00685	-	-	X	X	-	X	-
SLA	SRC-LIKE-ADAPTOR	6503	-1.145782	0.0066093	-	-	X	X	-	-	X
TRIP10	THYROID HORMONE RECEPTOR INTERACTOR 10	9322	-1.201569	0.0009053	-	-	X	X	-	-	X
ADORA3	ADENOSINE A3 RECEPTOR	140	-1.342378	0.0008809	-	-	X	X	-	-	X
TLR5	TOLL-LIKE RECEPTOR 5	7100	-1.318934	0.0002003	-	-	X	X	-	-	-
ACP5	ACID PHOSPHATASE 5, TARTRATE RESISTANT	54	-1.095127	0.0421248	-	-	X	X	-	-	-
MTDH	METADHERIN	92140	-1.113046	0.033133	-	-	X	X	-	-	-
P2RY6	PYRIMIDINERGIC RECEPTOR P2Y, G-PROTEIN COUPLED, 6	5031	-1.118231	0.0405457	-	-	X	X	-	-	-
PELI1	PELLINO HOMOLOG 1 (DROSOPHILA)	57162	-1.127696	0.0495851	-	-	X	X	-	-	-
GLRX2	GLUTAREDOXIN 2	51022	-1.132056	0.0189088	-	-	X	X	-	-	-
CAMLG	CALCIUM MODULATING LIGAND	819	-1.13552	0.0411787	-	-	X	X	-	-	-
GBP1	GUANYLATE BINDING PROTEIN 1, INTERFERON-INDUCIBLE, 67KDA	2633	-1.159622	0.0440595	-	-	X	X	-	-	-
PPP3R1	PROTEIN PHOSPHATASE 3 (FORMERLY 2B), REGULATORY SUBUNIT B, 19KDA, ALPHA ISOFORM (CALCINEURIN B, TYPE I)	5534	-1.1639	0.03451	-	-	X	X	-	-	-
BATF	BASIC LEUCINE ZIPPER TRANSCRIPTION FACTOR, ATF-LIKE	10538	-1.172606	0.0014913	-	-	X	X	-	-	-
STK39	SERINE THREONINE KINASE 39 (STE20/SPS1 HOMOLOG, YEAST)	27347	-1.188726	0.0269206	-	-	X	X	-	-	-
LXN	LATEXIN	56925	-1.208306	0.0103919	-	-	X	X	-	-	-
FPR1	FORMYL PEPTIDE RECEPTOR 1	2357	-1.216843	0.0005826	-	-	X	X	-	-	-
ZBTB7A	ZINC FINGER AND BTB DOMAIN CONTAINING 7A	51341	-1.098898	0.0486972	-	-	X	-	X	X	-
ACPP	ACID PHOSPHATASE, PROSTATE	55	-1.170794	0.032204	-	-	X	-	X	X	-
CHI3L1	CHITINASE 3-LIKE 1 (CARTILAGE GLYCOPROTEIN-39)	1116	-1.210294	0.0093417	-	-	X	-	X	X	-
EXT1	EXOSTOSES (MULTIPLE) 1	2131	-1.160885	0.0319773	-	-	X	-	X	-	X
F8	COAGULATION FACTOR VIII, PROCOAGULANT COMPONENT (HEMOPHILIA A)	2157	-1.191863	0.0016976	-	-	X	-	X	-	X
PYCARD	PYD AND CARD DOMAIN CONTAINING	29108	-1.1826	0.0124132	-	-	X	-	X	-	-
TLR8	TOLL-LIKE RECEPTOR 8	51311	-1.323112	0.0021936	-	-	X	-	X	-	-
LYL1	LYMPHOBLASTIC LEUKEMIA DERIVED SEQUENCE 1	4066	-1.089925	0.0374519	-	-	X	-	X	-	-
NDUFV2	NADH DEHYDROGENASE (UBIQUINONE) FLAVOPROTEIN 2, 24KDA	4729	-1.109133	0.0404742	-	-	X	-	X	-	-
TNFSF13	TUMOR NECROSIS FACTOR (LIGAND) SUPERFAMILY, MEMBER 13	8741	-1.109936	0.0418372	-	-	X	-	X	-	-
PPAP2A	PHOSPHATIDIC ACID PHOSPHATASE TYPE 2A	8611	-1.117242	0.0432702	-	-	X	-	X	-	-
ATP5E	ATP SYNTHASE, H+ TRANSPORTING, MITOCHONDRIAL F1 COMPLEX, EPSILON SUBUNIT	514	-1.118368	0.048741	-	-	X	-	X	-	-
UBE2E4P	UBIQUITIN-CONJUGATING ENZYME E2E 3 (UBC4/5 HOMOLOG, YEAST)	10477	-1.140465	0.0358171	-	-	X	-	X	-	-
SSB	SJOGREN SYNDROME ANTIGEN B (AUTOANTIGEN LA)	6741	-1.142493	0.007959	-	-	X	-	X	-	-
TRIM24	TRIPARTITE MOTIF-CONTAINING 24	8805	-1.157415	0.0115367	-	-	X	-	X	-	-
EIF2AK4	EUKARYOTIC TRANSLATION INITIATION FACTOR 2 ALPHA KINASE 4	440275	-1.188739	0.019527	-	-	X	-	X	-	-
CCL18	CHEMOKINE (C-C MOTIF) LIGAND 18 (PULMONARY AND ACTIVATION-REGULATED)	6362	-1.401858	0.0048651	-	-	X	-	-	X	-
LILRB2	LEUKOCYTE IMMUNOGLOBULIN-LIKE RECEPTOR, SUBFAMILY B (WITH TM AND ITIM DOMAINS), MEMBER 2	10288	-1.104854	0.0379968	-	-	X	-	-	X	-
POU2AF1	POU DOMAIN, CLASS 2, ASSOCIATING FACTOR 1	5450	-1.117463	0.0485338	-	-	X	-	-	X	-
SULT2B1	SULFOTRANSFERASE FAMILY, CYTOSOLIC, 2B, MEMBER 1	6820	-1.146025	0.0411008	-	-	X	-	-	X	-
PARD6A	PAR-6 PARTITIONING DEFECTIVE 6 HOMOLOG ALPHA (C.ELEGANS)	50855	-1.106429	0.0292279	-	-	X	-	-	-	X
SGK3	SERUM/GLUCOCORTICOID REGULATED KINASE FAMILY, MEMBER 3	23678	-1.170917	0.0154308	-	-	X	-	-	-	X
STAG2	STROMAL ANTIGEN 2	10735	-1.114865	0.0374377	-	-	X	-	-	-	-
LAMP3	LYSOSOMAL-ASSOCIATED MEMBRANE PROTEIN 3	27074	-1.145336	0.004299	-	-	X	-	-	-	-
IL12B	INTERLEUKIN 12B (NATURAL KILLER CELL STIMULATORY FACTOR 2, CYTOTOXIC LYMPHOCYTE MATURATION FACTOR 2, P40)	3593	-1.276274	0.0393269	-	-	X	-	-	-	-
IFI27	INTERFERON, ALPHA-INDUCIBLE PROTEIN 27	3429	-1.111231	0.0380865	-	-	X	-	-	-	-
STRN3	STRIATIN, CALMODULIN BINDING PROTEIN 3	29966	-1.113692	0.0299771	-	-	X	-	-	-	-
AP1B1	ADAPTOR-RELATED PROTEIN COMPLEX 1, BETA 1 SUBUNIT	162	-1.114217	0.0215013	-	-	X	-	-	-	-
ESD	ESTERASE D/FORMYLGLUTATHIONE HYDROLASE	2098	-1.117877	0.0413493	-	-	X	-	-	-	-
RGS10	REGULATOR OF G-PROTEIN SIGNALLING 10	6001	-1.119062	0.0235578	-	-	X	-	-	-	-
MAL	MAL, T-CELL DIFFERENTIATION PROTEIN	4118	-1.12159	0.0477366	-	-	X	-	-	-	-
ATOX1	ATX1 ANTIOXIDANT PROTEIN 1 HOMOLOG (YEAST)	475	-1.127995	0.0171042	-	-	X	-	-	-	-
STIM1	STROMAL INTERACTION MOLECULE 1	6786	-1.152342	0.0042869	-	-	X	-	-	-	-
CLEC4D	C-TYPE LECTIN-LIKE RECEPTOR 6	338339	-1.159928	0.0319351	-	-	X	-	-	-	-
ATG10	HYPOTHETICAL PROTEIN FLJ13954	83734	-1.169149	0.00835	-	-	X	-	-	-	-
GNG11	GUANINE NUCLEOTIDE BINDING PROTEIN (G PROTEIN), GAMMA 11	2791	-1.203741	0.0183315	-	-	X	-	-	-	-
EBI3	EPSTEIN-BARR VIRUS INDUCED GENE 3	10148	-1.203943	0.0036131	-	-	X	-	-	-	-
MT1M	METALLOTHIONEIN 1G	4499	-1.209561	0.0421802	-	-	X	-	-	-	-
ETV5	ETS VARIANT GENE 5 (ETS-RELATED MOLECULE)	2119	-1.347346	0.0031939	-	-	-	X	X	X	X
LMNB1	LAMIN B1	4001	-1.16033	0.0424466	-	-	-	X	X	X	-
CTSH	CATHEPSIN H	1512	-1.170733	0.0044175	-	-	-	X	X	X	-
PDPN	PODOPLANIN	10630	-1.190601	0.0055849	-	-	-	X	X	X	-
WWP1	WW DOMAIN CONTAINING E3 UBIQUITIN PROTEIN LIGASE 1	11059	-1.170074	0.0183829	-	-	-	X	X	-	X
H1F0	H1 HISTONE FAMILY, MEMBER 0	3005	-1.133434	0.0090986	-	-	-	X	X	-	-
	JUN DIMERIZATION PROTEIN 2	122953	-1.136025	0.0395723	-	-	-	X	X	-	-
MMP8	MATRIX METALLOPEPTIDASE 8 (NEUTROPHIL COLLAGENASE)	4317	-1.152052	0.0246393	-	-	-	X	X	-	-
CASP5	CASPASE 5, APOPTOSIS-RELATED CYSTEINE PEPTIDASE	838	-1.159866	0.0173156	-	-	-	X	X	-	-
GCA	GRANCALCIN, EF-HAND CALCIUM BINDING PROTEIN	25801	-1.190042	0.021554	-	-	-	X	X	-	-
ATP2C1	ATPASE, CA++ TRANSPORTING, TYPE 2C, MEMBER 1	27032	-1.133175	0.0286051	-	-	-	X	-	X	X
GYG1	GLYCOGENIN 1	2992	-1.097957	0.0254107	-	-	-	X	-	X	-
PCCB	PROPIONYL COENZYME A CARBOXYLASE, BETA POLYPEPTIDE	5096	-1.178114	0.0091354	-	-	-	X	-	X	-
GLTP	GLYCOLIPID TRANSFER PROTEIN	51228	-1.090194	0.0482235	-	-	-	X	-	-	-
VAMP8	VESICLE-ASSOCIATED MEMBRANE PROTEIN 8 (ENDOBREVIN)	8673	-1.114554	0.0369165	-	-	-	X	-	-	-
AZU1	AZUROCIDIN 1 (CATIONIC ANTIMICROBIAL PROTEIN 37)	566	-1.120699	0.042459	-	-	-	X	-	-	-
SLN	SARCOLIPIN	6588	-1.127112	0.0271523	-	-	-	X	-	-	-
AP1S2	ADAPTOR-RELATED PROTEIN COMPLEX 1, SIGMA 2 SUBUNIT	8905	-1.137431	0.0045881	-	-	-	X	-	-	-
SERPINB7	SERPIN PEPTIDASE INHIBITOR, CLADE B (OVALBUMIN), MEMBER 7	8710	-1.138297	0.0366392	-	-	-	X	-	-	-
ATF5	ACTIVATING TRANSCRIPTION FACTOR 5	22809	-1.147807	0.0353178	-	-	-	X	-	-	-
AANAT	ARYLALKYLAMINE N-ACETYLTRANSFERASE	15	-1.163419	0.0259112	-	-	-	X	-	-	-
PROK2	PROKINETICIN 2	60675	-1.169178	0.0476226	-	-	-	X	-	-	-
BASP1	BRAIN ABUNDANT, MEMBRANE ATTACHED SIGNAL PROTEIN 1	10409	-1.189484	0.0069699	-	-	-	X	-	-	-
ANXA3	ANNEXIN A3	306	-1.421232	3.57E-05	-	-	-	X	-	-	-
TCP1	T-COMPLEX 1	6950	-1.098446	0.0477542	-	-	-	-	X	X	-
CNDP2	CNDP DIPEPTIDASE 2 (METALLOPEPTIDASE M20 FAMILY)	55748	-1.104816	0.0338293	-	-	-	-	X	X	-
DCK	DEOXYCYTIDINE KINASE	1633	-1.148351	0.0263228	-	-	-	-	X	X	-
MNDA	MYELOID CELL NUCLEAR DIFFERENTIATION ANTIGEN	4332	-1.255802	0.0023399	-	-	-	-	X	X	-

Gene Name	Gene Description	Entrez ID	Mean FC	*p*-value	PPRE (Lemay)	PPRE	NFkB	JUN	TP53	SP1	CTNNB1
DPYSL2	DIHYDROPYRIMIDINASE-LIKE 2	1808	-1.379984	6.02E-05	-	-	-	-	X	X	-
CEACAM6	CARCINOEMBRYONIC ANTIGEN-RELATED CELL ADHESION MOLECULE 6 (NON-SPECIFIC CROSS REACTING ANTIGEN)	4680	-1.111261	0.0364025	-	-	-	-	X	-	X
MSH3	MUTS HOMOLOG 3 (E. COLI)	4437	-1.113627	0.0415125	-	-	-	-	X	-	X
C19orf33	CHROMOSOME 19 OPEN READING FRAME 33	64073	-1.129711	0.0387808	-	-	-	-	X	-	X
EFNB1	EPHRIN-B1	1947	-1.163656	0.0260756	-	-	-	-	X	-	X
GPNMB	GLYCOPROTEIN (TRANSMEMBRANE) NMB	10457	-1.407444	9.23E-06	-	-	-	-	X	-	X
DTYMK	DEOXYTHYMIDYLATE KINASE (THYMIDYLATE KINASE)	1841	-1.093312	0.0436781	-	-	-	-	X	-	-
POMP	CHROMOSOME 13 OPEN READING FRAME 12	51371	-1.108534	0.0404092	-	-	-	-	X	-	-
TIMM8B	TRANSLOCASE OF INNER MITOCHONDRIAL MEMBRANE 8 HOMOLOG B (YEAST)	26521	-1.113292	0.0243947	-	-	-	-	X	-	-
RFC2	REPLICATION FACTOR C (ACTIVATOR 1) 2, 40KDA	5982	-1.115968	0.0496638	-	-	-	-	X	-	-
CREG1	CELLULAR REPRESSOR OF E1A-STIMULATED GENES 1	8804	-1.120086	0.0429072	-	-	-	-	X	-	-
C21orf45	CHROMOSOME 21 OPEN READING FRAME 45	54069	-1.121171	0.0430327	-	-	-	-	X	-	-
GMNN	GEMININ, DNA REPLICATION INHIBITOR	51053	-1.126028	0.0324011	-	-	-	-	X	-	-
TOPORS	TOPOISOMERASE I BINDING, ARGININE/SERINE-RICH	10210	-1.132767	0.0453242	-	-	-	-	X	-	-
SRI	SORCIN	6717	-1.135121	0.0104541	-	-	-	-	X	-	-
SDCCAG8	SEROLOGICALLY DEFINED COLON CANCER ANTIGEN 8	10806	-1.137023	0.0183922	-	-	-	-	X	-	-
ARMC10	SVH PROTEIN	83787	-1.140455	0.0256157	-	-	-	-	X	-	-
	NUCLEAR DNA-BINDING PROTEIN	10438	-1.141979	0.017472	-	-	-	-	X	-	-
PODN	PODOCAN	127435	-1.149914	0.0227184	-	-	-	-	X	-	-
GTF2H5	GENERAL TRANSCRIPTION FACTOR IIH, POLYPEPTIDE 5	404672	-1.163696	0.0065977	-	-	-	-	X	-	-
	HYPOTHETICAL PROTEIN FLJ11259	55332	-1.16468	0.010886	-	-	-	-	X	-	-
IMPDH1	IMP (INOSINE MONOPHOSPHATE) DEHYDROGENASE 1	3614	-1.169506	0.0061298	-	-	-	-	X	-	-
CDKN2AIP	COLLABORATES/COOPERATES WITH ARF (ALTERNATE READING FRAME) PROTEIN	55602	-1.176391	0.0116499	-	-	-	-	X	-	-
CKS2	CDC28 PROTEIN KINASE REGULATORY SUBUNIT 2	1164	-1.18515	0.012657	-	-	-	-	X	-	-
TSNAX	TRANSLIN-ASSOCIATED FACTOR X	7257	-1.235848	0.0031316	-	-	-	-	X	-	-
IGSF6	IMMUNOGLOBULIN SUPERFAMILY, MEMBER 6	10261	-1.3087	0.0002524	-	-	-	-	X	-	-
OLIG1	OLIGODENDROCYTE TRANSCRIPTION FACTOR 1	116448	-1.341195	0.0011096	-	-	-	-	X	-	-
MSRB2	METHIONINE SULFOXIDE REDUCTASE B2	22921	-1.11133	0.0405387	-	-	-	-	-	X	-
CDCA7L	CELL DIVISION CYCLE ASSOCIATED 7-LIKE	55536	-1.125191	0.0403762	-	-	-	-	-	X	-
SRP9	SIGNAL RECOGNITION PARTICLE 9KDA	6726	-1.128623	0.0126046	-	-	-	-	-	X	-
CDK2AP1	CDK2-ASSOCIATED PROTEIN 1	8099	-1.141918	0.0172775	-	-	-	-	-	X	-
FKBP1B	FK506 BINDING PROTEIN 1B, 12.6 KDA	2281	-1.142334	0.0120565	-	-	-	-	-	X	-
PLCL1	PHOSPHOLIPASE C-LIKE 1	5334	-1.151723	0.0388965	-	-	-	-	-	X	-
LILRA2	LEUKOCYTE IMMUNOGLOBULIN-LIKE RECEPTOR, SUBFAMILY A (WITH TM DOMAIN), MEMBER 2	11027	-1.159322	0.007717	-	-	-	-	-	X	-
CSRP2	CYSTEINE AND GLYCINE-RICH PROTEIN 2	1466	-1.200711	0.0016308	-	-	-	-	-	X	-
KCNJ2	POTASSIUM INWARDLY-RECTIFYING CHANNEL, SUBFAMILY J, MEMBER 2	3759	-1.212394	0.0052964	-	-	-	-	-	X	-
CTSZ	CATHEPSIN Z	1522	-1.277364	0.0030363	-	-	-	-	-	X	-
PAPSS2	3'-PHOSPHOADENOSINE 5'-PHOSPHOSULFATE SYNTHASE 2	9060	-1.27879	0.0020034	-	-	-	-	-	X	-
KTN1	KINECTIN 1 (KINESIN RECEPTOR)	3895	-1.105691	0.0392875	-	-	-	-	-	-	X
MTMR2	MYOTUBULARIN RELATED PROTEIN 2	8898	-1.114398	0.0272037	-	-	-	-	-	-	X
TTC12	TETRATRICOPEPTIDE REPEAT DOMAIN 12	54970	-1.120432	0.0375907	-	-	-	-	-	-	X
FSCN1	FASCIN HOMOLOG 1, ACTIN-BUNDLING PROTEIN (STRONGYLOCENTROTUS PURPURATUS)	6624	-1.121379	0.0430706	-	-	-	-	-	-	X
PRCP	PROLYLCARBOXYPEPTIDASE (ANGIOTENSINASE C)	5547	-1.129218	0.034688	-	-	-	-	-	-	X
VASH1	VASOHIBIN 1	22846	-1.134063	0.0240812	-	-	-	-	-	-	X
LYPLA1	LYSOPHOSPHOLIPASE I	10434	-1.154367	0.0323873	-	-	-	-	-	-	X
LAMB3	LAMININ, BETA 3	3914	-1.154375	0.0420518	-	-	-	-	-	-	X
LY6G5B	LYMPHOCYTE ANTIGEN 6 COMPLEX, LOCUS G5B	58496	1.879472	4.82E-05	-	-	-	-	-	-	-
GOLGA8A	GOLGI AUTOANTIGEN, GOLGIN SUBFAMILY A, 8B	440270	1.667753	0.0008828	-	-	-	-	-	-	-
LOC283874	HYPOTHETICAL PROTEIN FLJ20393	283874	1.6616	8.72E-05	-	-	-	-	-	-	-
NA	RNA, U64 SMALL NUCLEOLAR	26784	1.653722	0.0017431	-	-	-	-	-	-	-
NA	FLJ38717 PROTEIN	401261	1.592361	0.0115702	-	-	-	-	-	-	-
REEP3	RECEPTOR ACCESSORY PROTEIN 3	221035	1.557306	0.0160273	-	-	-	-	-	-	-
NA	RAB12, MEMBER RAS ONCOGENE FAMILY	201475	1.538866	0.0024482	-	-	-	-	-	-	-
NA	T CELL RECEPTOR ALPHA JOINING 41	28714	1.526372	0.0272955	-	-	-	-	-	-	-
HS3ST1	HEPARAN SULFATE (GLUCOSAMINE) 3-O-SULFOTRANSFERASE 1	9957	1.512685	0.0002548	-	-	-	-	-	-	-
TKTL1	TRANSKETOLASE-LIKE 1	8277	1.502433	0.0245723	-	-	-	-	-	-	-
	HYPOTHETICAL PROTEIN FLJ25006	124923	1.488533	0.000896	-	-	-	-	-	-	-
NA	RNA, U72 SMALL NUCLEOLAR	26775	1.460701	0.0038881	-	-	-	-	-	-	-
ZNF514	ZINC FINGER PROTEIN 514	84874	1.456647	0.0034934	-	-	-	-	-	-	-
FLJ10213	HYPOTHETICAL PROTEIN FLJ10213	55096	1.450432	0.0009918	-	-	-	-	-	-	-
ATP8B4	ATPASE, CLASS I, TYPE 8B, MEMBER 4	79895	1.437528	0.007555	-	-	-	-	-	-	-
PIK3R5	PHOSPHOINOSITIDE-3-KINASE, REGULATORY SUBUNIT 5, P101	23533	1.432398	0.0046993	-	-	-	-	-	-	-
NA	METASTASIS ASSOCIATED LUNG ADENOCARCINOMA TRANSCRIPT 1 (NON-CODING RNA)	378938	1.431453	0.0086338	-	-	-	-	-	-	-
GABBR1	GAMMA-AMINOBUTYRIC ACID (GABA) B RECEPTOR, 1	2550	1.426144	0.0024803	-	-	-	-	-	-	-
GOLGA8A	GOLGI AUTOANTIGEN, GOLGIN SUBFAMILY A, 8A	23015	1.42222	0.000755	-	-	-	-	-	-	-
RNASE1	RIBONUCLEASE, RNASE A FAMILY, 1 (PANCREATIC)	6035	1.418564	0.0051646	-	-	-	-	-	-	-
ETFDH	ELECTRON-TRANSFERRING-FLAVOPROTEIN DEHYDROGENASE	2110	1.418314	0.0001317	-	-	-	-	-	-	-
FFAR2	FREE FATTY ACID RECEPTOR 2	2867	1.411246	0.0250116	-	-	-	-	-	-	-
LOC158830	SIMILAR TO AB2-183	158830	1.406762	0.0001507	-	-	-	-	-	-	-
USP34	UBIQUITIN SPECIFIC PEPTIDASE 34	9736	1.405321	0.0486316	-	-	-	-	-	-	-
C20orf119	CHROMOSOME 20 OPEN READING FRAME 119	80336	1.400493	0.0002804	-	-	-	-	-	-	-
C21orf7	CHROMOSOME 21 OPEN READING FRAME 7	56911	1.395685	0.0001791	-	-	-	-	-	-	-
MICALL2	MICAL-LIKE 2	79778	1.363246	0.000165	-	-	-	-	-	-	-
BZRAP1	BENZODIAZAPINE RECEPTOR (PERIPHERAL) ASSOCIATED PROTEIN 1	9256	1.359276	2.93E-05	-	-	-	-	-	-	-
	M-PHASE PHOSPHOPROTEIN, MPP8	54737	1.355857	0.0070679	-	-	-	-	-	-	-
NA	HYPOTHETICAL PROTEIN HS322B1A	25774	1.353304	0.0122333	-	-	-	-	-	-	-
	HYPOTHETICAL PROTEIN MGC14376	84981	1.348687	0.0024786	-	-	-	-	-	-	-
	NASOPHARYNGEAL CARCINOMA ASSOCIATED GENE PROTEIN-8	27099	1.328481	0.0072753	-	-	-	-	-	-	-
SLC6A6	SOLUTE CARRIER FAMILY 6 (NEUROTRANSMITTER TRANSPORTER, TAURINE), MEMBER 6	6533	1.325948	0.0139964	-	-	-	-	-	-	-
C19orf6	CHROMOSOME 19 OPEN READING FRAME 6	91304	1.325905	0.0121162	-	-	-	-	-	-	-
PILRB	PAIRED IMMUNOGLOBIN-LIKE TYPE 2 RECEPTOR BETA	29990	1.323695	0.0001577	-	-	-	-	-	-	-
CCDC84	SIMILAR TO DLNB14	338657	1.321056	0.006291	-	-	-	-	-	-	-
		728392	1.318199	0.0003225	-	-	-	-	-	-	-
AHSA2	AHA1, ACTIVATOR OF HEAT SHOCK 90KDA PROTEIN ATPASE HOMOLOG 2 (YEAST)	130872	1.318001	0.0015094	-	-	-	-	-	-	-
NA	AFG3 ATPASE FAMILY GENE 3-LIKE 1 (YEAST)	172	1.3175	0.0101148	-	-	-	-	-	-	-
METT11D1	FLJ20859 GENE	64745	1.316438	0.0022432	-	-	-	-	-	-	-
PPP1R3E	KIAA1443	90673	1.311025	0.000129	-	-	-	-	-	-	-
C17orf56	CHROMOSOME 17 OPEN READING FRAME 56	146705	1.304387	0.000554	-	-	-	-	-	-	-
AMY2B	AMYLASE, ALPHA 2B; PANCREATIC	280	1.303736	0.0093615	-	-	-	-	-	-	-
C21orf66	GC-RICH SEQUENCE DNA-BINDING FACTOR CANDIDATE	94104	1.30221	0.0033728	-	-	-	-	-	-	-
FAM13A1OS	FAMILY WITH SEQUENCE SIMILARITY 13, MEMBER A1 OPPOSITE STRAND	285512	1.301011	0.0005712	-	-	-	-	-	-	-
KIAA1468	KIAA1468	57614	1.30033	0.0250465	-	-	-	-	-	-	-
	MS4A13 PROTEIN	84689	1.29616	0.0033449	-	-	-	-	-	-	-
SRA1	AMYLOID BETA (A4) PRECURSOR PROTEIN-BINDING, FAMILY B, MEMBER 3	10307	1.291801	0.0004478	-	-	-	-	-	-	-
PARP15	POLY (ADP-RIBOSE) POLYMERASE FAMILY, MEMBER 15	165631	1.290632	0.0007658	-	-	-	-	-	-	-
PPIF	PEPTIDYLPROLYL ISOMERASE F (CYCLOPHILIN F)	10105	1.28669	0.0003693	-	-	-	-	-	-	-
C3orf62	CHROMOSOME 3 OPEN READING FRAME 62	375341	1.284501	0.0031359	-	-	-	-	-	-	-
CENPT	CHROMOSOME 16 OPEN READING FRAME 56	80152	1.284194	0.013471	-	-	-	-	-	-	-
SFRS15	SPLICING FACTOR, ARGININE/SERINE-RICH 15	57466	1.283173	0.0010925	-	-	-	-	-	-	-
DDX26B	DEAD/H (ASP-GLU-ALA-ASP/HIS) BOX POLYPEPTIDE 26B	203522	1.281855	0.0051982	-	-	-	-	-	-	-
ZNF224	ZINC FINGER PROTEIN 224	7767	1.279023	0.0045246	-	-	-	-	-	-	-
NA	HYPOTHETICAL LOC197135	197135	1.276714	0.0107338	-	-	-	-	-	-	-
ZBTB20	ZINC FINGER AND BTB DOMAIN CONTAINING 20	26137	1.273672	0.0223477	-	-	-	-	-	-	-
KCTD7	POTASSIUM CHANNEL TETRAMERISATION DOMAIN CONTAINING 7	154881	1.271696	0.0020926	-	-	-	-	-	-	-
NA	HYPOTHETICAL LOC344887	344887	1.27053	0.0023282	-	-	-	-	-	-	-
SUPT16H	SUPPRESSOR OF TY 16 HOMOLOG (S. CEREVISIAE)	11198	1.269654	0.0057717	-	-	-	-	-	-	-
ANKRD44	ANKYRIN REPEAT DOMAIN 44	91526	1.265273	0.0468072	-	-	-	-	-	-	-
NA	HIPPOCAMPUS ABUNDANT GENE TRANSCRIPT-LIKE 2	84278	1.264609	0.002961	-	-	-	-	-	-	-
		727852	1.263894	0.0007678	-	-	-	-	-	-	-
FLJ13197	HYPOTHETICAL PROTEIN FLJ13197	79667	1.263894	0.0007678	-	-	-	-	-	-	-
CCDC131	KIAA0546 PROTEIN	196441	1.263155	0.0027262	-	-	-	-	-	-	-
CD300A	CD300A ANTIGEN	11314	1.262597	0.000591	-	-	-	-	-	-	-
TARBP1	TAR (HIV-1) RNA BINDING PROTEIN 1	6894	1.260177	0.0009011	-	-	-	-	-	-	-

Gene Name	Gene Description	Entrez ID	Mean FC	*p*- value	PPRE (Lemay)	PPRE	NFkB	JUN	TP53	SP1	CTNNB1
CREBZF	HCF-BINDING TRANSCRIPTION FACTOR ZHANGFEI	58487	1.256312	0.0025145	-	-	-	-	-	-	-
ANKRD57	CHROMOSOME 2 OPEN READING FRAME 26	65124	1.25412	0.0011246	-	-	-	-	-	-	-
GTPBP2	GTP BINDING PROTEIN 2	54676	1.253013	0.0047571	-	-	-	-	-	-	-
PBX4	PRE-B-CELL LEUKEMIA TRANSCRIPTION FACTOR 4	80714	1.249177	0.0324698	-	-	-	-	-	-	-
CCDC17	COILED-COIL DOMAIN CONTAINING 17	149483	1.248747	0.0006349	-	-	-	-	-	-	-
NA	DDEF1 INTRONIC TRANSCRIPT 1	29065	1.247888	0.0287608	-	-	-	-	-	-	-
NA	HYPOTHETICAL LOCUS LOC338799	338799	1.244473	0.0023136	-	-	-	-	-	-	-
MPP7	MEMBRANE PROTEIN, PALMITOYLATED 7 (MAGUK P55 SUBFAMILY MEMBER 7)	143098	1.243443	0.0008444	-	-	-	-	-	-	-
NA	HYPOTHETICAL PROTEIN LOC221442	221442	1.242747	0.001539	-	-	-	-	-	-	-
CALCOCO1	CALCIUM BINDING AND COILED-COIL DOMAIN 1	57658	1.240853	0.0012992	-	-	-	-	-	-	-
SLC7A6	SOLUTE CARRIER FAMILY 7 (CATIONIC AMINO ACID TRANSPORTER, Y+ SYSTEM), MEMBER 6	9057	1.238805	0.0011314	-	-	-	-	-	-	-
NA	HYPOTHETICAL GENE SUPPORTED BY AK128346	440993	1.238667	0.0077761	-	-	-	-	-	-	-
USP9Y	UBIQUITIN SPECIFIC PEPTIDASE 9, Y-LINKED (FAT FACETS-LIKE, DROSOPHILA)	8287	1.236298	0.003669	-	-	-	-	-	-	-
C6orf111	CHROMOSOME 6 OPEN READING FRAME 111	25957	1.234771	0.0117828	-	-	-	-	-	-	-
		728225	1.234591	0.0102154	-	-	-	-	-	-	-
PIGL	PHOSPHATIDYLINOSITOL GLYCAN, CLASS L	9487	1.231785	0.010411	-	-	-	-	-	-	-
VAMP1	VESICLE-ASSOCIATED MEMBRANE PROTEIN 1 (SYNAPTOBREVIN 1)	6843	1.231429	0.0036674	-	-	-	-	-	-	-
COG3	COMPONENT OF OLIGOMERIC GOLGI COMPLEX 3	83548	1.2289	0.0006066	-	-	-	-	-	-	-
ADHFE1	ALCOHOL DEHYDROGENASE, IRON CONTAINING, 1	137872	1.227959	0.018287	-	-	-	-	-	-	-
B3GNT5	UDP-GLCNAC:BETAGAL BETA-1,3-N-ACETYLGLUCOSAMINYLTRANSFERASE 5	84002	1.225996	0.0014113	-	-	-	-	-	-	-
ATP6V1G2	HLA-B ASSOCIATED TRANSCRIPT 1	7919	1.225006	0.0049709	-	-	-	-	-	-	-
NA	SIMILAR TO R28379_1	390940	1.224694	0.0073424	-	-	-	-	-	-	-
TUBGCP6	TUBULIN, GAMMA COMPLEX ASSOCIATED PROTEIN 6	85378	1.224663	0.0012392	-	-	-	-	-	-	-
NSUN5B	NOL1/NOP2/SUN DOMAIN FAMILY, MEMBER 5B	155400	1.224562	0.0039234	-	-	-	-	-	-	-
RGL2	RAL GUANINE NUCLEOTIDE DISSOCIATION STIMULATOR-LIKE 2	5863	1.222443	0.0023328	-	-	-	-	-	-	-
MOGAT2	MONOACYLGLYCEROL O-ACYLTRANSFERASE 2	80168	1.221962	0.0248951	-	-	-	-	-	-	-
UHRF2	UBIQUITIN-LIKE, CONTAINING PHD AND RING FINGER DOMAINS, 2	115426	1.217584	0.0007408	-	-	-	-	-	-	-
ANKFY1	ANKYRIN REPEAT AND FYVE DOMAIN CONTAINING 1	51479	1.216773	0.00659	-	-	-	-	-	-	-
ATG16L2	ATG16 AUTOPHAGY RELATED 16-LIKE 2 (S. CEREVISIAE)	89849	1.216145	0.0305297	-	-	-	-	-	-	-
	HYPOTHETICAL PROTEIN LOC647217	647217	1.216005	0.0080315	-	-	-	-	-	-	-
ADPGK	ADP-DEPENDENT GLUCOKINASE	83440	1.215314	0.0156645	-	-	-	-	-	-	-
AKAP10	A KINASE (PRKA) ANCHOR PROTEIN 10	11216	1.214191	0.005904	-	-	-	-	-	-	-
NA	T CELL RECEPTOR ALPHA VARIABLE 8-3	28683	1.214115	0.004921	-	-	-	-	-	-	-
C1orf63	CHROMOSOME 1 OPEN READING FRAME 63	57035	1.213518	0.0194136	-	-	-	-	-	-	-
NA	HYPOTHETICAL PROTEIN LOC283663	283663	1.213124	0.0025048	-	-	-	-	-	-	-
CXorf45	CHROMOSOME X OPEN READING FRAME 45	79868	1.212752	0.0027593	-	-	-	-	-	-	-
NA	SIMILAR TO RNA-BINDING REGION (RNP1, RRM) CONTAINING 3	654340	1.211234	0.0198319	-	-	-	-	-	-	-
MTL5	METALLOTHIONEIN-LIKE 5, TESTIS-SPECIFIC (TESMIN)	9633	1.210152	0.0098386	-	-	-	-	-	-	-
RAB8B	RAB8B, MEMBER RAS ONCOGENE FAMILY	51762	1.210124	0.0076048	-	-	-	-	-	-	-
ECHDC2	ENOYL COENZYME A HYDRATASE DOMAIN CONTAINING 2	55268	1.20616	0.006675	-	-	-	-	-	-	-
FAM80B	FAMILY WITH SEQUENCE SIMILARITY 80, MEMBER B	57494	1.205015	0.0054452	-	-	-	-	-	-	-
ZNF655	ZINC FINGER PROTEIN 655	79027	1.204964	0.0126696	-	-	-	-	-	-	-
EIF4A2	EUKARYOTIC TRANSLATION INITIATION FACTOR 4A, ISOFORM 2	1974	1.204304	0.0390564	-	-	-	-	-	-	-
DTWD1	DTW DOMAIN CONTAINING 1	56986	1.204065	0.037276	-	-	-	-	-	-	-
ORMDL1	HYPOTHETICAL PROTEIN LOC51240	94101	1.20405	0.0039894	-	-	-	-	-	-	-
NA	CHROMOSOME 10 OPEN READING FRAME 31	414196	1.203988	0.0146713	-	-	-	-	-	-	-
OTUD3	OTU DOMAIN CONTAINING 3	23252	1.20397	0.0051029	-	-	-	-	-	-	-
NA	SIALIC ACID BINDING IG-LIKE LECTIN, PSEUDOGENE 16	400709	1.203136	0.0100518	-	-	-	-	-	-	-
	ACID-INDUCIBLE PHOSPHOPROTEIN	51747	1.202646	0.0016736	-	-	-	-	-	-	-
SPATA16	SPERMATOGENESIS ASSOCIATED 16	83893	1.202289	0.0052485	-	-	-	-	-	-	-
LENG8	LEUKOCYTE RECEPTOR CLUSTER (LRC) MEMBER 8	114823	1.202223	0.0235487	-	-	-	-	-	-	-
LTB4DH	LEUKOTRIENE B4 12-HYDROXYDEHYDROGENASE	22949	1.201318	0.0246855	-	-	-	-	-	-	-
C16orf48	CHROMOSOME 16 OPEN READING FRAME 48	84080	1.201104	0.0034523	-	-	-	-	-	-	-
ADAT1	ADENOSINE DEAMINASE, TRNA-SPECIFIC 1	23536	1.200738	0.0130366	-	-	-	-	-	-	-
ZMYND15	ZINC FINGER, MYND-TYPE CONTAINING 15	84225	1.199809	0.0055412	-	-	-	-	-	-	-
EML4	ECHINODERM MICROTUBULE ASSOCIATED PROTEIN LIKE 4	27436	1.199295	0.0087341	-	-	-	-	-	-	-
ACAD11	ACYL-COENZYME A DEHYDROGENASE FAMILY, MEMBER 11	84129	1.198369	0.0013305	-	-	-	-	-	-	-
SFRS14	SPLICING FACTOR, ARGININE/SERINE-RICH 14	10147	1.197377	0.0081843	-	-	-	-	-	-	-
ZSCAN18	ZINC FINGER PROTEIN 447	65982	1.196877	0.0067636	-	-	-	-	-	-	-
SNRP70	SMALL NUCLEAR RIBONUCLEOPROTEIN 70KDA POLYPEPTIDE (RNP ANTIGEN)	6625	1.196559	0.0033029	-	-	-	-	-	-	-
ABHD5	ABHYDROLASE DOMAIN CONTAINING 5	51099	1.196504	0.029569	-	-	-	-	-	-	-
MTMR15	KIAA1018	22909	1.196267	0.0085934	-	-	-	-	-	-	-
HP1BP3	HETEROCHROMATIN PROTEIN 1, BINDING PROTEIN 3	50809	1.195779	0.0138153	-	-	-	-	-	-	-
CCDC130	HYPOTHETICAL PROTEIN MGC10471	81576	1.195734	0.0011834	-	-	-	-	-	-	-
SEC31B	SEC31-LIKE 2 (S. CEREVISIAE)	25956	1.195585	0.0171709	-	-	-	-	-	-	-
CCDC49	COILED-COIL DOMAIN CONTAINING 49	54883	1.194413	0.0070964	-	-	-	-	-	-	-
	KIAA1530 PROTEIN	57654	1.194047	0.0410283	-	-	-	-	-	-	-
KIFC2	KINESIN FAMILY MEMBER C2	90990	1.192879	0.0104903	-	-	-	-	-	-	-
TTC31	HYPOTHETICAL PROTEIN FLJ12788	64427	1.192498	0.0026321	-	-	-	-	-	-	-
	HYPOTHETICAL PROTEIN FLJ10154	55082	1.192117	0.0048019	-	-	-	-	-	-	-
ELMO2	ENGULFMENT AND CELL MOTILITY 2	63916	1.191233	0.0061489	-	-	-	-	-	-	-
MFSD8	HYPOTHETICAL PROTEIN MGC33302	256471	1.190966	0.0073226	-	-	-	-	-	-	-
TMEM16H	TRANSMEMBRANE PROTEIN 16H	57719	1.190164	0.0161332	-	-	-	-	-	-	-
GSDML	GASDERMIN-LIKE	55876	1.190119	0.0071113	-	-	-	-	-	-	-
CYB5D1	CYTOCHROME B5 DOMAIN CONTAINING 1	124637	1.189477	0.0258855	-	-	-	-	-	-	-
HIST1H2BC	HISTONE 1, H2BD	8343	1.189425	0.0386733	-	-	-	-	-	-	-
WDR19	WD REPEAT DOMAIN 19	57728	1.189411	0.0041476	-	-	-	-	-	-	-
CST2	CYSTATIN SA	1470	1.189326	0.0239494	-	-	-	-	-	-	-
NA	TAURINE UPREGULATED GENE 1	55000	1.189266	0.0028949	-	-	-	-	-	-	-
NA	DENN/MADD DOMAIN CONTAINING 4B	9909	1.188246	0.005094	-	-	-	-	-	-	-
DDX11	DEAD/H (ASP-GLU-ALA-ASP/HIS) BOX POLYPEPTIDE 11 (CHL1-LIKE HELICASE HOMOLOG, S. CEREVISIAE)	1663	1.187521	0.0052936	-	-	-	-	-	-	-
SPG7	CELL MATRIX ADHESION REGULATOR	6687	1.187454	0.0009283	-	-	-	-	-	-	-
ZNF75	ZINC FINGER PROTEIN 75 (D8C6)	7626	1.187314	0.0145652	-	-	-	-	-	-	-
ZNF605	ZINC FINGER PROTEIN 605	90462	1.187287	0.0052488	-	-	-	-	-	-	-
MSH5	MUTS HOMOLOG 5 (E. COLI)	4439	1.186244	0.0203044	-	-	-	-	-	-	-
PRPF4B	PRP4 PRE-MRNA PROCESSING FACTOR 4 HOMOLOG B (YEAST)	8899	1.186175	0.0125448	-	-	-	-	-	-	-
KIAA1407	KIAA1407	57577	1.186043	0.0205577	-	-	-	-	-	-	-
NUP85	NUCLEOPORIN 85KDA	79902	1.185232	0.0027413	-	-	-	-	-	-	-
ZRANB2	ZINC FINGER PROTEIN 265	9406	1.185143	0.0102811	-	-	-	-	-	-	-
SIN3B	SIN3 HOMOLOG B, TRANSCRIPTION REGULATOR (YEAST)	23309	1.184751	0.0039449	-	-	-	-	-	-	-
TBC1D12	TBC1 DOMAIN FAMILY, MEMBER 12	23232	1.184559	0.0251685	-	-	-	-	-	-	-
PRAMEF12	PRAME FAMILY MEMBER 1	390999	1.184268	0.0158827	-	-	-	-	-	-	-
CRIPAK	HYPOTHETICAL PROTEIN FLJ34443	285464	1.184051	0.0152832	-	-	-	-	-	-	-
RUTBC1	RUN AND TBC1 DOMAIN CONTAINING 1	9905	1.183359	0.0109026	-	-	-	-	-	-	-
ZNF692	ZINC FINGER PROTEIN 692	55657	1.183195	0.0032822	-	-	-	-	-	-	-
NA	SIMILAR TO NON-POU DOMAIN CONTAINING, OCTAMER-BINDING	644035	1.182914	0.0172941	-	-	-	-	-	-	-
NA	SIMILAR TO LEIOMODIN 2 (CARDIAC)	442721	1.182438	0.0095302	-	-	-	-	-	-	-
SF3B1	SPLICING FACTOR 3B, SUBUNIT 1, 155KDA	23451	1.181633	0.0162794	-	-	-	-	-	-	-
RBM25	RNA BINDING MOTIF PROTEIN 25	58517	1.181342	0.0079712	-	-	-	-	-	-	-
NEK9	NIMA (NEVER IN MITOSIS GENE A)- RELATED KINASE 9	91754	1.180547	0.0173324	-	-	-	-	-	-	-
	HYPOTHETICAL PROTEIN FLJ20186	54849	1.180155	0.0086959	-	-	-	-	-	-	-
SIPA1L2	SIGNAL-INDUCED PROLIFERATION-ASSOCIATED 1 LIKE 2	57568	1.179671	0.0046108	-	-	-	-	-	-	-
		729559	1.179157	0.001934	-	-	-	-	-	-	-
PTGDR	PROSTAGLANDIN D2 RECEPTOR (DP)	5729	1.179112	0.0124522	-	-	-	-	-	-	-
	HYPOTHETICAL PROTEIN FLJ31033	91351	1.178376	0.0041672	-	-	-	-	-	-	-
PIGG	PHOSPHATIDYLINOSITOL GLYCAN, CLASS G	54872	1.177772	0.0199393	-	-	-	-	-	-	-
SLC26A11	SOLUTE CARRIER FAMILY 26, MEMBER 11	284129	1.177362	0.0127138	-	-	-	-	-	-	-
PGD	PHOSPHOGLUCONATE DEHYDROGENASE	5226	1.177191	0.0148603	-	-	-	-	-	-	-
CLEC5A	C-TYPE LECTIN DOMAIN FAMILY 5, MEMBER A	23601	1.176972	0.0020502	-	-	-	-	-	-	-
ST8SIA5	ST8 ALPHA-N-ACETYL-NEURAMINIDE ALPHA-2,8-SIALYLTRANSFERASE 5	29906	1.176511	0.0048022	-	-	-	-	-	-	-
FAM38A	FAMILY WITH SEQUENCE SIMILARITY 38, MEMBER A	9780	1.175424	0.003684	-	-	-	-	-	-	-
SS18L1	SYNOVIAL SARCOMA TRANSLOCATION GENE ON CHROMOSOME 18-LIKE 1	26039	1.175264	0.0070065	-	-	-	-	-	-	-
	HYPOTHETICAL PROTEIN LOC129530	129530	1.174951	0.0268567	-	-	-	-	-	-	-

Gene Name	Gene Description	Entrez ID	Mean FC	*p*-value	PPRE (Lemay)	PPRE	NFkB	JUN	TP53	SP1	CTNNB1
ACAD8	ACYL-COENZYME A DEHYDROGENASE FAMILY, MEMBER 8	27034	1.174864	0.0168375	-	-	-	-	-	-	-
PRPF38B	PRP38 PRE-MRNA PROCESSING FACTOR 38 (YEAST) DOMAIN CONTAINING B	55119	1.174834	0.0412416	-	-	-	-	-	-	-
EZH1	ENHANCER OF ZESTE HOMOLOG 1 (DROSOPHILA)	2145	1.174492	0.0045065	-	-	-	-	-	-	-
SEC61A2	SEC61 ALPHA 2 SUBUNIT (S. CEREVISIAE)	55176	1.173933	0.0347049	-	-	-	-	-	-	-
ZNF767	HYPOTHETICAL PROTEIN FLJ12700	79970	1.173526	0.0055114	-	-	-	-	-	-	-
LRRC6	LEUCINE RICH REPEAT CONTAINING 6	23639	1.173443	0.0251949	-	-	-	-	-	-	-
CD93	CD93 ANTIGEN	22918	1.172768	0.0208013	-	-	-	-	-	-	-
C6orf70	CHROMOSOME 6 OPEN READING FRAME 70	55780	1.172734	0.0021914	-	-	-	-	-	-	-
NA	RHOMBOID DOMAIN CONTAINING 2	57414	1.172611	0.005051	-	-	-	-	-	-	-
PDE8A	PHOSPHODIESTERASE 8A	5151	1.172327	0.0218764	-	-	-	-	-	-	-
DDX3X	DEAD (ASP-GLU-ALA-ASP) BOX POLYPEPTIDE 3, Y-LINKED	8653	1.172307	0.0118411	-	-	-	-	-	-	-
	HYPOTHETICAL PROTEIN FLJ32549	144577	1.172154	0.0380278	-	-	-	-	-	-	-
BTN3A1	BUTYROPHILIN, SUBFAMILY 3, MEMBER A1	11119	1.172036	0.0019339	-	-	-	-	-	-	-
ITGA10	INTEGRIN, ALPHA 10	8515	1.171626	0.0307048	-	-	-	-	-	-	-
C9orf80	CHROMOSOME 9 OPEN READING FRAME 80	58493	1.171024	0.0397031	-	-	-	-	-	-	-
WDR73	WD REPEAT DOMAIN 73	84942	1.170669	0.027537	-	-	-	-	-	-	-
PRR14	HYPOTHETICAL PROTEIN MGC3121	78994	1.170668	0.004488	-	-	-	-	-	-	-
LUC7L	LUC7-LIKE (S. CEREVISIAE)	55692	1.170299	0.0059868	-	-	-	-	-	-	-
CCDC45	COILED-COIL DOMAIN CONTAINING 45	90799	1.170203	0.0052371	-	-	-	-	-	-	-
TRIM41	TRIPARTITE MOTIF-CONTAINING 41	90933	1.17003	0.0102356	-	-	-	-	-	-	-
DDX17	DEAD (ASP-GLU-ALA-ASP) BOX POLYPEPTIDE 17	10521	1.169396	0.0052429	-	-	-	-	-	-	-
ITSN2	SH3 DOMAIN PROTEIN 1B	50618	1.169002	0.0026987	-	-	-	-	-	-	-
DNM2	QUEUINE TRNA-RIBOSYLTRANSFERASE 1 (TRNA-GUANINE TRANSGLYCOSYLASE)	81890	1.16777	0.0367611	-	-	-	-	-	-	-
ODF2	OUTER DENSE FIBER OF SPERM TAILS 2	4957	1.167653	0.0082177	-	-	-	-	-	-	-
COQ10A	COENZYME Q10 HOMOLOG A (YEAST)	93058	1.167541	0.0060289	-	-	-	-	-	-	-
NA	TRANSPOSON-DERIVED BUSTER1 TRANSPOSASE-LIKE PROTEIN GENE	58486	1.167273	0.01525	-	-	-	-	-	-	-
ZNF600	ZINC FINGER PROTEIN 600	162966	1.167271	0.0428906	-	-	-	-	-	-	-
BCDIN3D	HYPOTHETICAL PROTEIN LOC144233	144233	1.165869	0.0359855	-	-	-	-	-	-	-
ZNF570	ZINC FINGER PROTEIN 570	148268	1.165448	0.0091896	-	-	-	-	-	-	-
ARHGAP27	RHO GTPASE ACTIVATING PROTEIN 27	201176	1.164882	0.0267895	-	-	-	-	-	-	-
TMCO6	HYPOTHETICAL PROTEIN PRO1580	55374	1.163896	0.0039632	-	-	-	-	-	-	-
RNF214	HYPOTHETICAL PROTEIN DKFZP547C195	257160	1.163828	0.0170779	-	-	-	-	-	-	-
SMCHD1	STRUCTURAL MAINTENANCE OF CHROMOSOMES FLEXIBLE HINGE DOMAIN CONTAINING 1	23347	1.163472	0.0113673	-	-	-	-	-	-	-
NA	KIAA0125	9834	1.163462	0.002688	-	-	-	-	-	-	-
ABCA5	ATP-BINDING CASSETTE, SUB-FAMILY A (ABC1), MEMBER 5	23461	1.163421	0.0346257	-	-	-	-	-	-	-
NA	HYPOTHETICAL PROTEIN FLJ23584	79640	1.162952	0.0463213	-	-	-	-	-	-	-
METTL3	METHYLTRANSFERASE LIKE 3	56339	1.162656	0.0131107	-	-	-	-	-	-	-
	HYPOTHETICAL GENE SUPPORTED BY AK093779	399900	1.162262	0.0337752	-	-	-	-	-	-	-
NA	UNC-51-LIKE KINASE 3 (C. ELEGANS)	25989	1.161776	0.0032926	-	-	-	-	-	-	-
SUPT7L	SUPPRESSOR OF TY 7 (S. CEREVISIAE)-LIKE	9913	1.161453	0.007786	-	-	-	-	-	-	-
OR1I1	OLFACTORY RECEPTOR, FAMILY 1, SUBFAMILY I, MEMBER 1	126370	1.161397	0.0137294	-	-	-	-	-	-	-
ZNF248	ZINC FINGER PROTEIN 248	57209	1.161121	0.0172743	-	-	-	-	-	-	-
SFRS12	SPLICING FACTOR, ARGININE/SERINE-RICH 12	140890	1.160586	0.0167619	-	-	-	-	-	-	-
KIAA0141	KIAA0141	9812	1.160292	0.0087317	-	-	-	-	-	-	-
CRYGS	CRYSTALLIN, GAMMA S	1427	1.160289	0.014364	-	-	-	-	-	-	-
CCDC111	HYPOTHETICAL PROTEIN FLJ33167	201973	1.158123	0.0174617	-	-	-	-	-	-	-
GPR15	G PROTEIN-COUPLED RECEPTOR 15	2838	1.158078	0.0205056	-	-	-	-	-	-	-
KIAA1370	KIAA1370	56204	1.157891	0.0383517	-	-	-	-	-	-	-
ZNF137	ZINC FINGER PROTEIN 137 (CLONE PHZ-30)	7696	1.157829	0.0145909	-	-	-	-	-	-	-
C14orf118	CHROMOSOME 14 OPEN READING FRAME 118	55668	1.157545	0.0163662	-	-	-	-	-	-	-
CDK5RAP2	CDK5 REGULATORY SUBUNIT ASSOCIATED PROTEIN 2	55755	1.15729	0.0043495	-	-	-	-	-	-	-
DCUN1D4	DCN1, DEFECTIVE IN CULLIN NEDDYLATION 1, DOMAIN CONTAINING 4 (S. CEREVISIAE)	23142	1.156464	0.0305869	-	-	-	-	-	-	-
TMEM143	HYPOTHETICAL PROTEIN FLJ10922	55260	1.156311	0.0393842	-	-	-	-	-	-	-
TRIB2	TRIBBLES HOMOLOG 2 (DROSOPHILA)	28951	1.156114	0.0032127	-	-	-	-	-	-	-
TTLL4	TUBULIN TYROSINE LIGASE-LIKE FAMILY, MEMBER 4	9654	1.155923	0.0110752	-	-	-	-	-	-	-
ZNF592	ZINC FINGER PROTEIN 592	9640	1.155782	0.0405492	-	-	-	-	-	-	-
PCGF3	POLYCOMB GROUP RING FINGER 3	10336	1.155757	0.0081456	-	-	-	-	-	-	-
FBXO38	HYPOTHETICAL PROTEIN FLJ13962	81545	1.155723	0.0190233	-	-	-	-	-	-	-
ANKZF1	ANKYRIN REPEAT AND ZINC FINGER DOMAIN CONTAINING 1	55139	1.155531	0.0219468	-	-	-	-	-	-	-
SFI1	SFI1 HOMOLOG, SPINDLE ASSEMBLY ASSOCIATED (YEAST)	9814	1.154941	0.0130974	-	-	-	-	-	-	-
STARD5	START DOMAIN CONTAINING 5	80765	1.153384	0.0129941	-	-	-	-	-	-	-
MTERFD3	MTERF DOMAIN CONTAINING 3	80298	1.153327	0.0214932	-	-	-	-	-	-	-
PRDM14	PR DOMAIN CONTAINING 14	63978	1.152783	0.0096683	-	-	-	-	-	-	-
	HYPOTHETICAL LOC339541	339541	1.152743	0.0150446	-	-	-	-	-	-	-
RAI16	RETINOIC ACID INDUCED 16	64760	1.152253	0.0188557	-	-	-	-	-	-	-
AP4B1	ADAPTOR-RELATED PROTEIN COMPLEX 4, BETA 1 SUBUNIT	10717	1.151725	0.0085396	-	-	-	-	-	-	-
TAF1C	TATA BOX BINDING PROTEIN (TBP)-ASSOCIATED FACTOR, RNA POLYMERASE I, C, 110KDA	9013	1.151466	0.0189944	-	-	-	-	-	-	-
FAM111A	FAMILY WITH SEQUENCE SIMILARITY 111, MEMBER A	63901	1.151194	0.0083248	-	-	-	-	-	-	-
ZNF276	ZINC FINGER PROTEIN 276	92822	1.15116	0.0109712	-	-	-	-	-	-	-
SNORA25	JOSEPHIN DOMAIN CONTAINING 3	79101	1.150818	0.0137333	-	-	-	-	-	-	-
CAPRIN2	C1Q DOMAIN CONTAINING 1	65981	1.150746	0.0129854	-	-	-	-	-	-	-
LRP5L	LOW DENSITY LIPOPROTEIN RECEPTOR-RELATED PROTEIN 5-LIKE	91355	1.150295	0.004755	-	-	-	-	-	-	-
C14orf124	CHROMOSOME 14 OPEN READING FRAME 124	56948	1.150163	0.0037936	-	-	-	-	-	-	-
INPP1	INOSITOL POLYPHOSPHATE-1-PHOSPHATASE	3628	1.149428	0.0054789	-	-	-	-	-	-	-
FUT10	FUCOSYLTRANSFERASE 10 (ALPHA (1,3) FUCOSYLTRANSFERASE)	84750	1.148948	0.026485	-	-	-	-	-	-	-
TMEM127	TRANSMEMBRANE PROTEIN 127	55654	1.148941	0.005486	-	-	-	-	-	-	-
C1orf71	CHROMOSOME 1 OPEN READING FRAME 71	163882	1.148904	0.0378454	-	-	-	-	-	-	-
	KIAA1706 PROTEIN	80820	1.148525	0.0164486	-	-	-	-	-	-	-
BRD9	BROMODOMAIN CONTAINING 9	65980	1.148371	0.0150749	-	-	-	-	-	-	-
NA	KIAA0913	23053	1.148336	0.0134937	-	-	-	-	-	-	-
PARP6	POLY (ADP-RIBOSE) POLYMERASE FAMILY, MEMBER 6	56965	1.147611	0.0041995	-	-	-	-	-	-	-
NA	CHROMOSOME 14 OPEN READING FRAME 138	79609	1.146803	0.0328116	-	-	-	-	-	-	-
ZDHHC6	ZINC FINGER, DHHC-TYPE CONTAINING 6	64429	1.14611	0.0288189	-	-	-	-	-	-	-
	KIAA1505 PROTEIN	57639	1.144883	0.0169828	-	-	-	-	-	-	-
SCRT2	SULFIREDOXIN 1 HOMOLOG (S. CEREVISIAE)	140809	1.144817	0.0219598	-	-	-	-	-	-	-
ARPC4	TUBULIN TYROSINE LIGASE-LIKE FAMILY, MEMBER 3	26140	1.144718	0.0243707	-	-	-	-	-	-	-
TRAF3IP3	TRAF3 INTERACTING PROTEIN 3	80342	1.144683	0.0102931	-	-	-	-	-	-	-
WDR59	WD REPEAT DOMAIN 59	79726	1.144173	0.0111857	-	-	-	-	-	-	-
ZBTB48	GLI-KRUPPEL FAMILY MEMBER HKR3	3104	1.144167	0.0107685	-	-	-	-	-	-	-
NA	SIMILAR TO RIKEN CDNA 1110012D08	440104	1.144035	0.0156486	-	-	-	-	-	-	-
SERPINB8	SERPIN PEPTIDASE INHIBITOR, CLADE B (OVALBUMIN), MEMBER 8	5271	1.143994	0.020279	-	-	-	-	-	-	-
	HYPOTHETICAL PROTEIN BC009732	133308	1.14309	0.0431976	-	-	-	-	-	-	-
C14orf131	CHROMOSOME 14 OPEN READING FRAME 131	55778	1.142999	0.0254322	-	-	-	-	-	-	-
NA	MHC CLASS I MRNA FRAGMENT 3.8-1	352961	1.141973	0.0451092	-	-	-	-	-	-	-
CSNK1D	CASEIN KINASE 1, DELTA	1453	1.141936	0.0124443	-	-	-	-	-	-	-
MBD6	METHYL-CPG BINDING DOMAIN PROTEIN 6	114785	1.141883	0.0287339	-	-	-	-	-	-	-
DDX39	DEAD (ASP-GLU-ALA-ASP) BOX POLYPEPTIDE 39	10212	1.141059	0.0099199	-	-	-	-	-	-	-
MUC20	MUCIN 20	200958	1.140829	0.0247833	-	-	-	-	-	-	-
C11orf56	CHROMOSOME 11 OPEN READING FRAME 56	84067	1.140763	0.0391259	-	-	-	-	-	-	-
ZNF548	ZINC FINGER PROTEIN 548	147694	1.140563	0.0149551	-	-	-	-	-	-	-
KIAA0355	KIAA0355	9710	1.140436	0.0088638	-	-	-	-	-	-	-
SLC35B2	SOLUTE CARRIER FAMILY 35, MEMBER B2	347734	1.140387	0.0112078	-	-	-	-	-	-	-
ELMO3	ENGULFMENT AND CELL MOTILITY 3	79767	1.140302	0.0126115	-	-	-	-	-	-	-
TJAP1	TIGHT JUNCTION ASSOCIATED PROTEIN 1 (PERIPHERAL)	93643	1.140153	0.0075641	-	-	-	-	-	-	-
ANKHD1	ANKYRIN REPEAT AND KH DOMAIN CONTAINING 1	54882	1.139278	0.0209991	-	-	-	-	-	-	-
DNAJB5	DNAJ (HSP40) HOMOLOG, SUBFAMILY B, MEMBER 5	25822	1.13915	0.0138147	-	-	-	-	-	-	-
SENP7	SUMO1/SENTRIN SPECIFIC PEPTIDASE 7	57337	1.138104	0.0196493	-	-	-	-	-	-	-
KIAA0907	KIAA0907	22889	1.138047	0.0233271	-	-	-	-	-	-	-
CARS2	HYPOTHETICAL PROTEIN FLJ12118	79587	1.138001	0.0129964	-	-	-	-	-	-	-
	STROMAL ANTIGEN 3-LIKE	64940	1.137897	0.0157231	-	-	-	-	-	-	-
POLRMT	POLYMERASE (RNA) MITOCHONDRIAL (DNA DIRECTED)	5442	1.137243	0.0363176	-	-	-	-	-	-	-
	FLJ42957 PROTEIN	400077	1.137161	0.0130556	-	-	-	-	-	-	-
CCRK	CELL CYCLE RELATED KINASE	23552	1.137154	0.0119449	-	-	-	-	-	-	-
MTX3	METAXIN 3	345778	1.135439	0.0052483	-	-	-	-	-	-	-
	PRE-MRNA CLEAVAGE FACTOR I, 59 KDA SUBUNIT	79869	1.135035	0.0075484	-	-	-	-	-	-	-

Gene Name	Gene Description	Entrez ID	Mean FC	*p*-value	PPRE (Lemay)	PPRE	NFkB	JUN	TP53	SP1	CTNNB1
ZNF619	ZINC FINGER PROTEIN 619	285267	1.134743	0.0437492	-	-	-	-	-	-	-
NA	CHROMOSOME 14 OPEN READING FRAME 62	317786	1.13437	0.0435239	-	-	-	-	-	-	-
CRYAA	CRYSTALLIN, ALPHA A	1409	1.134274	0.0488509	-	-	-	-	-	-	-
SFRS11	SPLICING FACTOR, ARGININE/SERINE-RICH 11	9295	1.134115	0.0173368	-	-	-	-	-	-	-
MAEA	MACROPHAGE ERYTHROBLAST ATTACHER	10296	1.134047	0.0140819	-	-	-	-	-	-	-
DHX33	DEAH (ASP-GLU-ALA-HIS) BOX POLYPEPTIDE 33	56919	1.133808	0.0137701	-	-	-	-	-	-	-
SPSB3	SPLA/RYANODINE RECEPTOR DOMAIN AND SOCS BOX CONTAINING 3	90864	1.13364	0.026724	-	-	-	-	-	-	-
ENOSF1	ENOLASE SUPERFAMILY MEMBER 1	55556	1.132829	0.0198699	-	-	-	-	-	-	-
MSL3L1	MALE-SPECIFIC LETHAL 3-LIKE 1 (DROSOPHILA)	10943	1.132612	0.0156018	-	-	-	-	-	-	-
NA	SURVIVAL-RELATED GENE	654487	1.132529	0.0441936	-	-	-	-	-	-	-
	HSPC049 PROTEIN	29062	1.132396	0.0228969	-	-	-	-	-	-	-
ADCK5	AARF DOMAIN CONTAINING KINASE 5	203054	1.132186	0.0368421	-	-	-	-	-	-	-
GORASP1	GOLGI REASSEMBLY STACKING PROTEIN 1, 65KDA	64689	1.132132	0.0118071	-	-	-	-	-	-	-
QSOX2	QUIESCIN Q6-LIKE 1	169714	1.131706	0.0432172	-	-	-	-	-	-	-
DONSON	DOWNSTREAM NEIGHBOR OF SON	29980	1.131635	0.0308659	-	-	-	-	-	-	-
KIAA0195	KIAA0195	9772	1.13125	0.0261435	-	-	-	-	-	-	-
DISP1	HYPOTHETICAL PROTEIN DKFZP434I0428	84976	1.130942	0.034871	-	-	-	-	-	-	-
C1orf144	CHROMOSOME 1 OPEN READING FRAME 144	26099	1.129795	0.0228685	-	-	-	-	-	-	-
HIST1H2BC	HISTONE 1, H2BD	8346	1.129579	0.0205507	-	-	-	-	-	-	-
	ZINC FINGER DAZ INTERACTING PROTEIN 3	9666	1.129268	0.0313821	-	-	-	-	-	-	-
		727768	1.128778	0.0421327	-	-	-	-	-	-	-
VNN3	VANIN 3	55350	1.128671	0.0134192	-	-	-	-	-	-	-
RSHL2	RADIAL SPOKEHEAD-LIKE 2	83861	1.128514	0.0426471	-	-	-	-	-	-	-
FAM48A	FAMILY WITH SEQUENCE SIMILARITY 48, MEMBER A	55578	1.12823	0.0364965	-	-	-	-	-	-	-
ZNF134	ZINC FINGER PROTEIN 211	10520	1.128105	0.0271682	-	-	-	-	-	-	-
ZNF490	ZINC FINGER PROTEIN 490	57474	1.127513	0.0179267	-	-	-	-	-	-	-
STK36	SERINE/THREONINE KINASE 36 (FUSED HOMOLOG, DROSOPHILA)	27148	1.127414	0.0384332	-	-	-	-	-	-	-
GFOD1	GLUCOSE-FRUCTOSE OXIDOREDUCTASE DOMAIN CONTAINING 1	54438	1.127058	0.0294984	-	-	-	-	-	-	-
NPFF	NEUROPEPTIDE FF-AMIDE PEPTIDE PRECURSOR	8620	1.127051	0.0210196	-	-	-	-	-	-	-
		728844	1.126713	0.0154648	-	-	-	-	-	-	-
PSME3	PROTEASOME (PROSOME, MACROPAIN) ACTIVATOR SUBUNIT 3 (PA28 GAMMA; KI)	10197	1.126704	0.047745	-	-	-	-	-	-	-
ZNF513	ZINC FINGER PROTEIN 513	130557	1.126428	0.0357197	-	-	-	-	-	-	-
FAM113A	FAMILY WITH SEQUENCE SIMILARITY 113, MEMBER A	64773	1.126386	0.0249718	-	-	-	-	-	-	-
	VALYL-TRNA SYNTHETASE LIKE	57176	1.126118	0.0306361	-	-	-	-	-	-	-
PPP1R15B	PROTEIN PHOSPHATASE 1, REGULATORY (INHIBITOR) SUBUNIT 15B	84919	1.125993	0.033645	-	-	-	-	-	-	-
FCHO1	FCH DOMAIN ONLY 1	23149	1.124874	0.0327506	-	-	-	-	-	-	-
ARRB2	ARRESTIN, BETA 2	409	1.124558	0.0355642	-	-	-	-	-	-	-
AAAS	ACHALASIA, ADRENOCORTICAL INSUFFICIENCY, ALACRIMIA (ALLGROVE, TRIPLE-A)	8086	1.124523	0.0107922	-	-	-	-	-	-	-
ANKHD1	ANKYRIN REPEAT AND KH DOMAIN CONTAINING 1	404734	1.12364	0.0382476	-	-	-	-	-	-	-
RNPC3	RNA-BINDING REGION (RNP1, RRM) CONTAINING 3	55599	1.12358	0.0358383	-	-	-	-	-	-	-
DOM3Z	DOM-3 HOMOLOG Z (C. ELEGANS)	1797	1.122939	0.0256801	-	-	-	-	-	-	-
ZNF512	ZINC FINGER PROTEIN 512	84450	1.122722	0.0266253	-	-	-	-	-	-	-
TMEM55B	TRANSMEMBRANE PROTEIN 55B	90809	1.122717	0.0193005	-	-	-	-	-	-	-
KIAA0692	KIAA0692	23141	1.12256	0.026752	-	-	-	-	-	-	-
FAM73B	FAMILY WITH SEQUENCE SIMILARITY 73, MEMBER B	84895	1.122312	0.0399832	-	-	-	-	-	-	-
GHDC	HOMOLOG OF MOUSE LGP1	84514	1.122259	0.0218572	-	-	-	-	-	-	-
TTC14	TETRATRICOPEPTIDE REPEAT DOMAIN 14	151613	1.121687	0.0244599	-	-	-	-	-	-	-
PHF8	PHD FINGER PROTEIN 8	23133	1.121316	0.0319933	-	-	-	-	-	-	-
TBRG4	TRANSFORMING GROWTH FACTOR BETA REGULATOR 4	9238	1.121151	0.0431498	-	-	-	-	-	-	-
GANAB	GLUCOSIDASE, ALPHA; NEUTRAL AB	23193	1.120961	0.0255717	-	-	-	-	-	-	-
RBED1	RNA BINDING MOTIF AND ELMO/CED-12 DOMAIN 1	84173	1.120245	0.0411099	-	-	-	-	-	-	-
ANKRD49	ANKYRIN REPEAT DOMAIN 49	54851	1.120164	0.0221916	-	-	-	-	-	-	-
PPP1R12C	PROTEIN PHOSPHATASE 1, REGULATORY (INHIBITOR) SUBUNIT 12C	54776	1.12011	0.0210271	-	-	-	-	-	-	-
PLXNA3	PLEXIN A3	55558	1.12009	0.0389588	-	-	-	-	-	-	-
ZNF597	ZINC FINGER PROTEIN 597	146434	1.119935	0.0417125	-	-	-	-	-	-	-
PSCD4	PLECKSTRIN HOMOLOGY, SEC7 AND COILED-COIL DOMAINS 4	27128	1.119464	0.04579	-	-	-	-	-	-	-
ZCCHC6	ZINC FINGER, CCHC DOMAIN CONTAINING 6	79670	1.11938	0.034014	-	-	-	-	-	-	-
FCHSD1	FCH AND DOUBLE SH3 DOMAINS 1	89848	1.119319	0.0344599	-	-	-	-	-	-	-
TBC1D10C	TBC1 DOMAIN FAMILY, MEMBER 10C	374403	1.117947	0.0133473	-	-	-	-	-	-	-
CXXC1	CXXC FINGER 1 (PHD DOMAIN)	30827	1.117378	0.0209672	-	-	-	-	-	-	-
POLE	POLYMERASE (DNA DIRECTED), EPSILON	5426	1.116965	0.0433143	-	-	-	-	-	-	-
PRB3	PROLINE-RICH PROTEIN BSTNI SUBFAMILY 3	5544	1.116706	0.0280927	-	-	-	-	-	-	-
ZFAND2A	ZINC FINGER, AN1-TYPE DOMAIN 2A	90637	1.116503	0.0163845	-	-	-	-	-	-	-
C3orf19	CHROMOSOME 3 OPEN READING FRAME 19	51244	1.116431	0.0358095	-	-	-	-	-	-	-
TRIM38	TRIPARTITE MOTIF-CONTAINING 38	10475	1.115941	0.026717	-	-	-	-	-	-	-
WDR48	WD REPEAT DOMAIN 48	57599	1.115196	0.0280237	-	-	-	-	-	-	-
PCP2	PURKINJE CELL PROTEIN 2	126006	1.114766	0.0466432	-	-	-	-	-	-	-
ZSWIM5	ZINC FINGER, SWIM-TYPE CONTAINING 5	57643	1.114675	0.0262097	-	-	-	-	-	-	-
	SIMILAR TO ZINC FINGER PROTEIN 74 (HZNF7)	643641	1.114389	0.0177854	-	-	-	-	-	-	-
		728568	1.113959	0.0487215	-	-	-	-	-	-	-
SFRS8	SPLICING FACTOR, ARGININE/SERINE-RICH 8 (SUPPRESSOR-OF-WHITE-APRICOT HOMOLOG, DROSOPHILA)	6433	1.113842	0.0234939	-	-	-	-	-	-	-
AUP1	ANCIENT UBIQUITOUS PROTEIN 1	550	1.113105	0.0378828	-	-	-	-	-	-	-
ACAD11	NEPHRONOPHTHISIS 3 (ADOLESCENT)	27031	1.112981	0.0330863	-	-	-	-	-	-	-
DIP2C	DIP2 DISCO-INTERACTING PROTEIN 2 HOMOLOG C (DROSOPHILA)	22982	1.112786	0.0246173	-	-	-	-	-	-	-
ZFYVE1	ZINC FINGER, FYVE DOMAIN CONTAINING 1	53349	1.112715	0.0380159	-	-	-	-	-	-	-
RHOT2	RAS HOMOLOG GENE FAMILY, MEMBER T2	89941	1.111889	0.0431421	-	-	-	-	-	-	-
RSRC2	SIMILAR TO SPLICING FACTOR, ARGININE/SERINE-RICH 4	65117	1.111156	0.043074	-	-	-	-	-	-	-
ACTR5	ARP5 ACTIN-RELATED PROTEIN 5 HOMOLOG (YEAST)	79913	1.111087	0.0273519	-	-	-	-	-	-	-
KIAA0174	KIAA0174	9798	1.11086	0.0258994	-	-	-	-	-	-	-
PTCD3	FLJ20758 PROTEIN	55037	1.110337	0.0327295	-	-	-	-	-	-	-
EYA3	EYES ABSENT HOMOLOG 3 (DROSOPHILA)	2140	1.110134	0.0260258	-	-	-	-	-	-	-
NGLY1	N-GLYCANASE 1	55768	1.110028	0.0324262	-	-	-	-	-	-	-
KNTC1	KINETOCHORE ASSOCIATED 1	9735	1.109905	0.0294943	-	-	-	-	-	-	-
EIF2B1	EUKARYOTIC TRANSLATION INITIATION FACTOR 2B, SUBUNIT 1 ALPHA, 26KDA	1967	1.109715	0.0356712	-	-	-	-	-	-	-
SLC7A10	HYPOTHETICAL PROTEIN FLJ20839	56301	1.109064	0.0464677	-	-	-	-	-	-	-
C14orf159	CHROMOSOME 14 OPEN READING FRAME 159	80017	1.108832	0.041434	-	-	-	-	-	-	-
C7orf47	HYPOTHETICAL PROTEIN MGC22793	221908	1.108793	0.046327	-	-	-	-	-	-	-
MOBKL2C	MOB1, MPS ONE BINDER KINASE ACTIVATOR-LIKE 2C (YEAST)	148932	1.108023	0.0224991	-	-	-	-	-	-	-
YRDC	YRDC DOMAIN CONTAINING (E. COLI)	79693	1.107352	0.0462113	-	-	-	-	-	-	-
NSUN3	NOL1/NOP2/SUN DOMAIN FAMILY, MEMBER 3	63899	1.106739	0.0425696	-	-	-	-	-	-	-
TTC16	TETRATRICOPEPTIDE REPEAT DOMAIN 16	158248	1.106124	0.0328669	-	-	-	-	-	-	-
RUSC1	RUN AND SH3 DOMAIN CONTAINING 1	23623	1.106039	0.0232337	-	-	-	-	-	-	-
STK11IP	SERINE/THREONINE KINASE 11 INTERACTING PROTEIN	114790	1.105664	0.0194895	-	-	-	-	-	-	-
MXD3	MAX DIMERIZATION PROTEIN 3	83463	1.105294	0.040976	-	-	-	-	-	-	-
EDD1	E3 UBIQUITIN PROTEIN LIGASE, HECT DOMAIN CONTAINING, 1	51366	1.10501	0.0492045	-	-	-	-	-	-	-
	KIAA1545 PROTEIN	57666	1.104952	0.041571	-	-	-	-	-	-	-
STIP1	STRESS-INDUCED-PHOSPHOPROTEIN 1 (HSP70/HSP90-ORGANIZING PROTEIN)	10963	1.104858	0.0264465	-	-	-	-	-	-	-
UBAP2L	UBIQUITIN ASSOCIATED PROTEIN 2-LIKE	9898	1.103887	0.0234603	-	-	-	-	-	-	-
RLN1	RELAXIN 1	6013	1.103669	0.0349057	-	-	-	-	-	-	-
C12orf30	CHROMOSOME 12 OPEN READING FRAME 30	80018	1.103035	0.0306912	-	-	-	-	-	-	-
SGTA	SMALL GLUTAMINE-RICH TETRATRICOPEPTIDE REPEAT (TPR)-CONTAINING, ALPHA	6449	1.101873	0.0495031	-	-	-	-	-	-	-
C17orf85	ELG PROTEIN	55421	1.101763	0.036612	-	-	-	-	-	-	-
DPEP2	DIPEPTIDASE 2	64174	1.101688	0.0273856	-	-	-	-	-	-	-
TMUB2	HYPOTHETICAL PROTEIN MGC3123	79089	1.101536	0.0440581	-	-	-	-	-	-	-
PMPCA	PEPTIDASE (MITOCHONDRIAL PROCESSING) ALPHA	23203	1.10143	0.030242	-	-	-	-	-	-	-
C19orf61	HYPOTHETICAL PROTEIN FLJ12886	56006	1.100745	0.0433876	-	-	-	-	-	-	-
NFRKB	NUCLEAR FACTOR RELATED TO KAPPAB BINDING PROTEIN	4798	1.100482	0.0369501	-	-	-	-	-	-	-
PHKG2	PHOSPHORYLASE KINASE, GAMMA 2 (TESTIS)	5261	1.099334	0.0421977	-	-	-	-	-	-	-
ROPN1L	ROPPORIN 1-LIKE	83853	1.09855	0.0472779	-	-	-	-	-	-	-
DHX15	DEAH (ASP-GLU-ALA-HIS) BOX POLYPEPTIDE 15	1665	1.098406	0.0420368	-	-	-	-	-	-	-
CECR2	CAT EYE SYNDROME CHROMOSOME REGION, CANDIDATE 2	27443	1.097941	0.0394526	-	-	-	-	-	-	-
ZNF182	ZINC FINGER PROTEIN 630	57232	1.097322	0.0328784	-	-	-	-	-	-	-
CARS	CYSTEINYL-TRNA SYNTHETASE	833	1.096524	0.0375823	-	-	-	-	-	-	-
CBFA2T2	CORE-BINDING FACTOR, RUNT DOMAIN, ALPHA SUBUNIT 2; TRANSLOCATED TO, 2	9139	1.096412	0.0370061	-	-	-	-	-	-	-

Gene Name	Gene Description	Entrez ID	Mean FC	*p*- value	PPRE (Lemay)	PPRE	NFkB	JUN	TP53	SP1	CTNNB1
DEFB118	DEFENSIN, BETA 118	117285	1.095708	0.0363198	-	-	-	-	-	-	-
NA	ZUOTIN RELATED FACTOR 1	27000	1.09555	0.0405	-	-	-	-	-	-	-
NOL8	NUCLEOLAR PROTEIN 8	55035	1.094919	0.0461197	-	-	-	-	-	-	-
PLEKHM2	PLECKSTRIN HOMOLOGY DOMAIN CONTAINING, FAMILY M (WITH RUN DOMAIN) MEMBER 2	23207	1.092754	0.0424969	-	-	-	-	-	-	-
METTL8	HYPOTHETICAL PROTEIN FLJ13984	79828	1.092131	0.0480882	-	-	-	-	-	-	-
	CHONDROITIN SULFATE GLUCURONYLTRANSFERASE	54480	1.091948	0.0450856	-	-	-	-	-	-	-
FAM108B1	CHROMOSOME 9 OPEN READING FRAME 77	51104	-1.088222	0.0398607	-	-	-	-	-	-	-
PARVB	PARVIN, BETA	29780	-1.091456	0.0382888	-	-	-	-	-	-	-
TES	TESTIS DERIVED TRANSCRIPT (3 LIM DOMAINS)	26136	-1.091919	0.0497563	-	-	-	-	-	-	-
KIAA1797	KIAA1797	54914	-1.092422	0.0395998	-	-	-	-	-	-	-
ALDH9A1	ALDEHYDE DEHYDROGENASE 9 FAMILY, MEMBER A1	223	-1.092937	0.0446182	-	-	-	-	-	-	-
PTPRA	PROTEIN TYROSINE PHOSPHATASE, RECEPTOR TYPE, A	5786	-1.093	0.0413327	-	-	-	-	-	-	-
HSD17B11	DEHYDROGENASE/REDUCTASE (SDR FAMILY) MEMBER 8	51170	-1.093541	0.0385491	-	-	-	-	-	-	-
SNUPN	RNA, U TRANSPORTER 1	10073	-1.09413	0.0436733	-	-	-	-	-	-	-
CRNKL1	CRN, CROOKED NECK-LIKE 1 (DROSOPHILA)	51340	-1.094324	0.030924	-	-	-	-	-	-	-
ALDH3B1	ALDEHYDE DEHYDROGENASE 3 FAMILY, MEMBER B1	221	-1.094343	0.0442461	-	-	-	-	-	-	-
MESDC2	MESODERM DEVELOPMENT CANDIDATE 2	23184	-1.094403	0.0321694	-	-	-	-	-	-	-
CDR2L	CEREBELLAR DEGENERATION-RELATED PROTEIN 2-LIKE	30850	-1.094467	0.040497	-	-	-	-	-	-	-
	KIAA0564 PROTEIN	23078	-1.094732	0.0386522	-	-	-	-	-	-	-
SIPA1L3	SIGNAL-INDUCED PROLIFERATION-ASSOCIATED 1 LIKE 3	23094	-1.09643	0.0324507	-	-	-	-	-	-	-
FER	FER (FPS/FES RELATED) TYROSINE KINASE (PHOSPHOPROTEIN NCP94)	2241	-1.096737	0.0425796	-	-	-	-	-	-	-
TRPM2	TRANSIENT RECEPTOR POTENTIAL CATION CHANNEL, SUBFAMILY M, MEMBER 2	7226	-1.097153	0.0322276	-	-	-	-	-	-	-
MRPL27	MITOCHONDRIAL RIBOSOMAL PROTEIN L27	51264	-1.098686	0.0459507	-	-	-	-	-	-	-
		AFFX-M27	-1.099469	0.0344683	-	-	-	-	-	-	-
SLAMF8	SLAM FAMILY MEMBER 8	56833	-1.099585	0.0284776	-	-	-	-	-	-	-
NA	HYPOTHETICAL PROTEIN LOC646762	646762	-1.10032	0.045474	-	-	-	-	-	-	-
	XTP3-TRANSACTIVATED PROTEIN A	79077	-1.100996	0.0448145	-	-	-	-	-	-	-
CCDC34	HYPOTHETICAL PROTEIN AF301222	91057	-1.101032	0.0480327	-	-	-	-	-	-	-
KIAA1191	KIAA1191	57179	-1.101315	0.045728	-	-	-	-	-	-	-
SERGEF	SECRETION REGULATING GUANINE NUCLEOTIDE EXCHANGE FACTOR	26297	-1.101403	0.0343629	-	-	-	-	-	-	-
	SIMILAR TO SRR1-LIKE PROTEIN	402055	-1.101578	0.0349323	-	-	-	-	-	-	-
CPNE8	COPINE VIII	144402	-1.101614	0.0426394	-	-	-	-	-	-	-
MFSD1	MAJOR FACILITATOR SUPERFAMILY DOMAIN CONTAINING 1	64747	-1.101651	0.0309399	-	-	-	-	-	-	-
CCDC12	COILED-COIL DOMAIN CONTAINING 12	151903	-1.101696	0.0392391	-	-	-	-	-	-	-
	HYPOTHETICAL PROTEIN FLJ20294	55626	-1.101854	0.0363729	-	-	-	-	-	-	-
UTP18	WD REPEAT DOMAIN 50	51096	-1.102187	0.0306054	-	-	-	-	-	-	-
COX7A2	CYTOCHROME C OXIDASE SUBUNIT VIIA POLYPEPTIDE 2 (LIVER)	1347	-1.102478	0.0459824	-	-	-	-	-	-	-
FAM49B	FAMILY WITH SEQUENCE SIMILARITY 49, MEMBER B	51571	-1.103261	0.0355781	-	-	-	-	-	-	-
NA	CYTOCHROME C OXIDASE SUBUNIT VIB POLYPEPTIDE 2 (TESTIS)	125965	-1.103904	0.0215101	-	-	-	-	-	-	-
SIGLEC7	SIALIC ACID BINDING IG-LIKE LECTIN 7	27036	-1.104011	0.0445953	-	-	-	-	-	-	-
ARSD	ARYLSULFATASE D	414	-1.104589	0.0368363	-	-	-	-	-	-	-
CCDC53	COILED-COIL DOMAIN CONTAINING 53	51019	-1.105118	0.0351301	-	-	-	-	-	-	-
KIR2DL1	KILLER CELL IMMUNOGLOBULIN-LIKE RECEPTOR, TWO DOMAINS, LONG CYTOPLASMIC TAIL, 3	3809	-1.105188	0.0482112	-	-	-	-	-	-	-
CBLN2	CEREBELLIN 2 PRECURSOR	147381	-1.106112	0.0475021	-	-	-	-	-	-	-
TBL2	DKFZP434N024 PROTEIN	26608	-1.106252	0.0392171	-	-	-	-	-	-	-
NA	GALECTIN-3 INTERNAL GENE	81625	-1.106458	0.0251063	-	-	-	-	-	-	-
TMEM142A	HYPOTHETICAL PROTEIN FLJ14466	84876	-1.107009	0.0424592	-	-	-	-	-	-	-
MRPS35	MITOCHONDRIAL RIBOSOMAL PROTEIN S35	60488	-1.107103	0.0376932	-	-	-	-	-	-	-
PABPC3	POLY(A) BINDING PROTEIN, CYTOPLASMIC 3	5042	-1.107194	0.0451484	-	-	-	-	-	-	-
GLT25D2	GLYCOSYLTRANSFERASE 25 DOMAIN CONTAINING 2	23127	-1.107214	0.0481334	-	-	-	-	-	-	-
WWC3	KIAA1280 PROTEIN	55841	-1.107277	0.0481385	-	-	-	-	-	-	-
MT1H	HYPOTHETICAL PROTEIN LOC645745	645745	-1.107323	0.0292898	-	-	-	-	-	-	-
SH3KBP1	SH3-DOMAIN KINASE BINDING PROTEIN 1	30011	-1.107735	0.0451802	-	-	-	-	-	-	-
WSB2	WD REPEAT AND SOCS BOX-CONTAINING 2	55884	-1.107873	0.0386884	-	-	-	-	-	-	-
UVRAG	UV RADIATION RESISTANCE ASSOCIATED GENE	7405	-1.107969	0.0400138	-	-	-	-	-	-	-
YEATS2	YEATS DOMAIN CONTAINING 2	55689	-1.10824	0.0467893	-	-	-	-	-	-	-
KATNA1	KATANIN P60 (ATPASE-CONTAINING) SUBUNIT A 1	11104	-1.108286	0.0351603	-	-	-	-	-	-	-
ELL3	ELONGATION FACTOR RNA POLYMERASE II-LIKE 3	80237	-1.108475	0.028114	-	-	-	-	-	-	-
C7orf48	HYPOTHETICAL PROTEIN MGC10911	84262	-1.109122	0.0285562	-	-	-	-	-	-	-
	SIMILAR TO TSG118.1	400506	-1.10983	0.0328431	-	-	-	-	-	-	-
TNFSF13	TUMOR NECROSIS FACTOR (LIGAND) SUPERFAMILY, MEMBER 12-MEMBER 13	407977	-1.109936	0.0418372	-	-	-	-	-	-	-
DYNLL2	DYNEIN, LIGHT CHAIN, LC8-TYPE 2	140735	-1.110133	0.0446136	-	-	-	-	-	-	-
ZBTB8OS	ZINC FINGER AND BTB DOMAIN CONTAINING 8 OPPOSITE STRAND	339487	-1.110472	0.0353602	-	-	-	-	-	-	-
FUBP3	FAR UPSTREAM ELEMENT (FUSE) BINDING PROTEIN 3	8939	-1.110781	0.0488042	-	-	-	-	-	-	-
ANKRD25	ANKYRIN REPEAT DOMAIN 25	25959	-1.110967	0.033537	-	-	-	-	-	-	-
SMNDC1	SURVIVAL MOTOR NEURON DOMAIN CONTAINING 1	10285	-1.111552	0.0428886	-	-	-	-	-	-	-
	DEVELOPMENTALLY REGULATED RNA-BINDING PROTEIN 1	129831	-1.111576	0.0281091	-	-	-	-	-	-	-
TRIM35	TRIPARTITE MOTIF-CONTAINING 35	23087	-1.111638	0.0470611	-	-	-	-	-	-	-
RAB32	RAB32, MEMBER RAS ONCOGENE FAMILY	10981	-1.111729	0.0340091	-	-	-	-	-	-	-
WDFY1	WD REPEAT AND FYVE DOMAIN CONTAINING 1	57590	-1.112302	0.0349224	-	-	-	-	-	-	-
SLC39A14	SOLUTE CARRIER FAMILY 39 (ZINC TRANSPORTER), MEMBER 14	23516	-1.112831	0.0410449	-	-	-	-	-	-	-
OBFC2B	OLIGONUCLEOTIDE/OLIGOSACCHARIDE-BINDING FOLD CONTAINING 2B	79035	-1.113091	0.0155616	-	-	-	-	-	-	-
SGMS2	HYPOTHETICAL PROTEIN MGC26963	166929	-1.113645	0.0484251	-	-	-	-	-	-	-
OSTF1	OSTEOCLAST STIMULATING FACTOR 1	26578	-1.113756	0.0226337	-	-	-	-	-	-	-
NA	CHROMOSOME X OPEN READING FRAME 42	158801	-1.113891	0.0337548	-	-	-	-	-	-	-
COPS4	COP9 CONSTITUTIVE PHOTOMORPHOGENIC HOMOLOG SUBUNIT 4 (ARABIDOPSIS)	51138	-1.114528	0.0316987	-	-	-	-	-	-	-
	SIMILAR TO SIMILAR TO RIKEN CDNA 2310002J15 GENE	653325	-1.114728	0.0185581	-	-	-	-	-	-	-
THUMPD1	THUMP DOMAIN CONTAINING 1	55623	-1.115019	0.0465243	-	-	-	-	-	-	-
KLHL5	KELCH-LIKE 5 (DROSOPHILA)	51088	-1.115056	0.0445654	-	-	-	-	-	-	-
	SIMILAR TO HSPC323	284422	-1.115084	0.0457136	-	-	-	-	-	-	-
MPST	MERCAPTOPYRUVATE SULFURTRANSFERASE	4357	-1.115087	0.0200527	-	-	-	-	-	-	-
RAMP1	RECEPTOR (CALCITONIN) ACTIVITY MODIFYING PROTEIN 1	10267	-1.115425	0.03828	-	-	-	-	-	-	-
CLNS1A	CHLORIDE CHANNEL, NUCLEOTIDE-SENSITIVE, 1A	1207	-1.115875	0.0374433	-	-	-	-	-	-	-
DHX29	DEAH (ASP-GLU-ALA-HIS) BOX POLYPEPTIDE 29	54505	-1.115962	0.046532	-	-	-	-	-	-	-
CSTF2T	CLEAVAGE STIMULATION FACTOR, 3' PRE-RNA, SUBUNIT 2, 64KDA, TAU VARIANT	23283	-1.116149	0.0202161	-	-	-	-	-	-	-
EIF2S3	EUKARYOTIC TRANSLATION INITIATION FACTOR 2, SUBUNIT 3 GAMMA, 52KDA	1968	-1.117245	0.037199	-	-	-	-	-	-	-
POLR1E	POLYMERASE (RNA) I ASSOCIATED FACTOR 1	64425	-1.117705	0.0477633	-	-	-	-	-	-	-
WDR40A	WD REPEAT DOMAIN 40A	25853	-1.118098	0.0183881	-	-	-	-	-	-	-
KIAA1598	KIAA1598	57698	-1.11835	0.0471707	-	-	-	-	-	-	-
ZNF35	ZINC FINGER PROTEIN 35 (CLONE HF.10)	7584	-1.118511	0.0180455	-	-	-	-	-	-	-
	TTMB PROTEIN	399474	-1.118588	0.0253916	-	-	-	-	-	-	-
FAM46C	FAMILY WITH SEQUENCE SIMILARITY 46, MEMBER C	54855	-1.119371	0.0330893	-	-	-	-	-	-	-
TTYH2	TWEETY HOMOLOG 2 (DROSOPHILA)	94015	-1.11956	0.0240889	-	-	-	-	-	-	-
ACTL7A	ACTIN-LIKE 7A	10881	-1.119598	0.0239896	-	-	-	-	-	-	-
CCDC105	HYPOTHETICAL PROTEIN FLJ40365	126402	-1.11973	0.037365	-	-	-	-	-	-	-
PINK1	PTEN INDUCED PUTATIVE KINASE 1	65018	-1.119885	0.012796	-	-	-	-	-	-	-
ZBTB3	ZINC FINGER AND BTB DOMAIN CONTAINING 3	79842	-1.120232	0.0358587	-	-	-	-	-	-	-
OR1F1	OLFACTORY RECEPTOR, FAMILY 1, SUBFAMILY F, MEMBER 1	4992	-1.120826	0.0368894	-	-	-	-	-	-	-
	SIMILAR TO CG14903-PA	391356	-1.1209	0.032417	-	-	-	-	-	-	-
	HYPOTHETICAL PROTEIN	26005	-1.121239	0.0196745	-	-	-	-	-	-	-
SACM1L	SAC1 SUPPRESSOR OF ACTIN MUTATIONS 1-LIKE (YEAST)	22908	-1.121472	0.0158438	-	-	-	-	-	-	-
SLC25A24	SOLUTE CARRIER FAMILY 25 (MITOCHONDRIAL CARRIER; PHOSPHATE CARRIER), MEMBER 24	29957	-1.121608	0.0306858	-	-	-	-	-	-	-
TIPRL	TIP41, TOR SIGNALLING PATHWAY REGULATOR-LIKE (S. CEREVISIAE)	261726	-1.121681	0.0413143	-	-	-	-	-	-	-
PPA1	PYROPHOSPHATASE (INORGANIC) 1	5464	-1.122286	0.0263553	-	-	-	-	-	-	-
THOC7	NGG1 INTERACTING FACTOR 3 LIKE 1 BINDING PROTEIN 1	80145	-1.122312	0.0449763	-	-	-	-	-	-	-
FAHD2A	FUMARYLACETOACETATE HYDROLASE DOMAIN CONTAINING 2A	51011	-1.122684	0.0252304	-	-	-	-	-	-	-
MRPL12	MITOCHONDRIAL RIBOSOMAL PROTEIN L12	6182	-1.122822	0.0265498	-	-	-	-	-	-	-
POLR1B	SIMILAR TO DNA-DIRECTED RNA POLYMERASE I (135 KDA)	84172	-1.122897	0.0283314	-	-	-	-	-	-	-
C14orf129	CHROMOSOME 14 OPEN READING FRAME 129	51527	-1.12298	0.020465	-	-	-	-	-	-	-
SUMF1	SULFATASE MODIFYING FACTOR 1	285362	-1.123697	0.0135184	-	-	-	-	-	-	-
BLVRA	BILIVERDIN REDUCTASE A	644	-1.123758	0.0177427	-	-	-	-	-	-	-
ZNF362	FLJ25476 PROTEIN	149076	-1.123788	0.0275556	-	-	-	-	-	-	-
CENTB2	CENTAURIN, BETA 2	23527	-1.124041	0.0433437	-	-	-	-	-	-	-

Gene Name	Gene Description	Entrez ID	Mean FC	*p*-value	PPRE (Lemay)	PPRE	NFkB	JUN	TP53	SP1	CTNNB1
IGLL3	SIMILAR TO OMEGA PROTEIN	91353	-1.124538	0.0364515	-	-	-	-	-	-	-
IQSEC2	IQ MOTIF AND SEC7 DOMAIN 2	23096	-1.124635	0.0485337	-	-	-	-	-	-	-
UBTD1	UBIQUITIN DOMAIN CONTAINING 1	80019	-1.125153	0.0332785	-	-	-	-	-	-	-
EDEM2	CHROMOSOME 20 OPEN READING FRAME 31	55741	-1.125235	0.0086357	-	-	-	-	-	-	-
	HYPOTHETICAL PROTEIN FLJ20489	55652	-1.125535	0.0458502	-	-	-	-	-	-	-
NA	HYPOTHETICAL GENE SUPPORTED BY BC038466; BC062790	401115	-1.125631	0.0161916	-	-	-	-	-	-	-
RAB31	RAB31, MEMBER RAS ONCOGENE FAMILY	11031	-1.125939	0.0144436	-	-	-	-	-	-	-
SLC4A1AP	SOLUTE CARRIER FAMILY 4 (ANION EXCHANGER), MEMBER 1, ADAPTOR PROTEIN	22950	-1.126	0.023911	-	-	-	-	-	-	-
C9orf167	HYPOTHETICAL PROTEIN FLJ20245	54863	-1.126165	0.0242484	-	-	-	-	-	-	-
MXRA7	FLJ46603 PROTEIN	439921	-1.126634	0.0297829	-	-	-	-	-	-	-
PPP1CB	PROTEIN PHOSPHATASE 1, CATALYTIC SUBUNIT, BETA ISOFORM	5500	-1.126809	0.0353865	-	-	-	-	-	-	-
C12orf23	CHROMOSOME 12 OPEN READING FRAME 23	90488	-1.126833	0.0405602	-	-	-	-	-	-	-
ACN9	ACN9 HOMOLOG (S. CEREVISIAE)	57001	-1.127118	0.0455765	-	-	-	-	-	-	-
DARS	ASPARTYL-TRNA SYNTHETASE	1615	-1.128303	0.0483069	-	-	-	-	-	-	-
NA	T CELL RECEPTOR ALPHA VARIABLE 27	28655	-1.128502	0.0424714	-	-	-	-	-	-	-
CHCHD3	COILED-COIL-HELIX-COILED-COIL-HELIX DOMAIN CONTAINING 3	54927	-1.128676	0.0445311	-	-	-	-	-	-	-
NR1D2	NUCLEAR RECEPTOR SUBFAMILY 1, GROUP D, MEMBER 2	9975	-1.128801	0.0367954	-	-	-	-	-	-	-
RPP40	RIBONUCLEASE P 40KDA SUBUNIT	10799	-1.128854	0.0104106	-	-	-	-	-	-	-
NTAN1	N-TERMINAL ASPARAGINE AMIDASE	123803	-1.128951	0.0236221	-	-	-	-	-	-	-
ANP32E	ACIDIC (LEUCINE-RICH) NUCLEAR PHOSPHOPROTEIN 32 FAMILY, MEMBER E	81611	-1.129201	0.0421187	-	-	-	-	-	-	-
ATP6V1H	ATPASE, H+ TRANSPORTING, LYSOSOMAL 50/57KDA, V1 SUBUNIT H	51606	-1.129211	0.0499417	-	-	-	-	-	-	-
PCID1	DENDRITIC CELL PROTEIN	10480	-1.129556	0.0284787	-	-	-	-	-	-	-
MRPL33	MITOCHONDRIAL RIBOSOMAL PROTEIN L33	9553	-1.130058	0.0255991	-	-	-	-	-	-	-
LGICZ1	LIGAND-GATED ION CHANNEL, ZINC ACTIVATED 1	353174	-1.13006	0.0204693	-	-	-	-	-	-	-
CAPZA1	CAPPING PROTEIN (ACTIN FILAMENT) MUSCLE Z-LINE, ALPHA 1	829	-1.130513	0.0424693	-	-	-	-	-	-	-
SGPL1	SPHINGOSINE-1-PHOSPHATE LYASE 1	8879	-1.130546	0.0260381	-	-	-	-	-	-	-
FCN1	FICOLIN (COLLAGEN/FIBRINOGEN DOMAIN CONTAINING) 1	2219	-1.130961	0.0134095	-	-	-	-	-	-	-
NA	CHROMOSOME 8 OPEN READING FRAME 59	401466	-1.131041	0.02685	-	-	-	-	-	-	-
C20orf29	CHROMOSOME 20 OPEN READING FRAME 29	55317	-1.131047	0.0323771	-	-	-	-	-	-	-
NUCB2	NUCLEOBINDIN 2	4925	-1.131757	0.0407727	-	-	-	-	-	-	-
SCML2	SEX COMB ON MIDLEG-LIKE 2 (DROSOPHILA)	10389	-1.132031	0.0218875	-	-	-	-	-	-	-
C6orf145	CHROMOSOME 6 OPEN READING FRAME 145	221749	-1.132035	0.0148029	-	-	-	-	-	-	-
BLVRB	BILIVERDIN REDUCTASE B (FLAVIN REDUCTASE (NADPH))	645	-1.13225	0.0223231	-	-	-	-	-	-	-
ALKBH3	ALKB, ALKYLATION REPAIR HOMOLOG 3 (E. COLI)	221120	-1.132343	0.0135116	-	-	-	-	-	-	-
RAP2A	RAP2A, MEMBER OF RAS ONCOGENE FAMILY	5911	-1.132742	0.0231628	-	-	-	-	-	-	-
LIMK2	LIM DOMAIN KINASE 2	3985	-1.132854	0.0380547	-	-	-	-	-	-	-
PFDN4	PREFOLDIN SUBUNIT 4	5203	-1.133228	0.021261	-	-	-	-	-	-	-
SIGLEC5	SIALIC ACID BINDING IG-LIKE LECTIN 5	8778	-1.133471	0.0152213	-	-	-	-	-	-	-
GLT1D1	GLYCOSYLTRANSFERASE 1 DOMAIN CONTAINING 1	144423	-1.133858	0.012265	-	-	-	-	-	-	-
MID1IP1	MID1 INTERACTING PROTEIN 1 (GASTRULATION SPECIFIC G12-LIKE (ZEBRAFISH))	58526	-1.134174	0.0110138	-	-	-	-	-	-	-
MRPL11	CGI-113 PROTEIN	65003	-1.134425	0.0306785	-	-	-	-	-	-	-
PRRT3	PROLINE-RICH TRANSMEMBRANE PROTEIN 3	285368	-1.134559	0.027325	-	-	-	-	-	-	-
	KIAA1754-LIKE	150771	-1.134643	0.0131861	-	-	-	-	-	-	-
NET1	NEUROEPITHELIAL CELL TRANSFORMING GENE 1	10276	-1.134844	0.0477247	-	-	-	-	-	-	-
NRM	NURIM (NUCLEAR ENVELOPE MEMBRANE PROTEIN)	11270	-1.134962	0.0258146	-	-	-	-	-	-	-
NLRP9	NACHT, LEUCINE RICH REPEAT AND PYD CONTAINING 9	338321	-1.13507	0.0184454	-	-	-	-	-	-	-
	CBF1 INTERACTING COREPRESSOR	9541	-1.135753	0.0361533	-	-	-	-	-	-	-
COX15	COX15 HOMOLOG, CYTOCHROME C OXIDASE ASSEMBLY PROTEIN (YEAST)	1355	-1.135838	0.0253014	-	-	-	-	-	-	-
PNPLA1	PATATIN-LIKE PHOSPHOLIPASE DOMAIN CONTAINING 1	285848	-1.136036	0.0192036	-	-	-	-	-	-	-
PPT1	PALMITOYL-PROTEIN THIOESTERASE 1 (CEROID-LIPOFUSCINOSIS, NEURONAL 1, INFANTILE)	5538	-1.136107	0.0213032	-	-	-	-	-	-	-
CNN2	CALPONIN 2	1265	-1.136651	0.0073716	-	-	-	-	-	-	-
RGL1	RAL GUANINE NUCLEOTIDE DISSOCIATION STIMULATOR-LIKE 1	23179	-1.136781	0.0161836	-	-	-	-	-	-	-
COTL1	COACTOSIN-LIKE 1 (DICTYOSTELIUM)	23406	-1.137769	0.0216617	-	-	-	-	-	-	-
JAKMIP1	JANUS KINASE AND MICROTUBULE INTERACTING PROTEIN 1	152789	-1.137842	0.0164127	-	-	-	-	-	-	-
C10orf125	CHROMOSOME 10 OPEN READING FRAME 125	282969	-1.138048	0.0080689	-	-	-	-	-	-	-
MYEOV	MYELOMA OVEREXPRESSED GENE (IN A SUBSET OF T(11;14) POSITIVE MULTIPLE MYELOMAS)	26579	-1.138151	0.0125314	-	-	-	-	-	-	-
ANKRD47	ANKYRIN REPEAT DOMAIN 47	256949	-1.139952	0.0382978	-	-	-	-	-	-	-
NFAM1	NFAT ACTIVATING PROTEIN WITH ITAM MOTIF 1	150372	-1.140516	0.0083413	-	-	-	-	-	-	-
C12orf32	CHROMOSOME 12 OPEN READING FRAME 32	83695	-1.140709	0.0254413	-	-	-	-	-	-	-
NA	E1A-LIKE INHIBITOR OF DIFFERENTIATION 3	493861	-1.141321	0.0412132	-	-	-	-	-	-	-
ARHGAP10	RHO GTPASE ACTIVATING PROTEIN 10	79658	-1.142347	0.0092519	-	-	-	-	-	-	-
NA	TESCALCIN	54997	-1.142407	0.0232411	-	-	-	-	-	-	-
	SIMILAR TO OPIOID BINDING PROTEIN/CELL ADHESION MOLECULE-LIKE	402665	-1.142676	0.0388926	-	-	-	-	-	-	-
CLIC3	CHLORIDE INTRACELLULAR CHANNEL 3	9022	-1.142981	0.0311085	-	-	-	-	-	-	-
ATP5I	ATP SYNTHASE, H+ TRANSPORTING, MITOCHONDRIAL F0 COMPLEX, SUBUNIT E	521	-1.143566	0.0229032	-	-	-	-	-	-	-
C6orf125	CHROMOSOME 6 OPEN READING FRAME 125	84300	-1.14413	0.0346924	-	-	-	-	-	-	-
CHCHD5	COILED-COIL-HELIX-COILED-COIL-HELIX DOMAIN CONTAINING 5	84269	-1.145077	0.0191784	-	-	-	-	-	-	-
DBR1	DEBRANCHING ENZYME HOMOLOG 1 (S. CEREVISIAE)	51163	-1.145342	0.0472849	-	-	-	-	-	-	-
C22orf9	CHROMOSOME 22 OPEN READING FRAME 9	23313	-1.145495	0.0130239	-	-	-	-	-	-	-
TCEAL4	TRANSCRIPTION ELONGATION FACTOR A (SII)-LIKE 4	79921	-1.145508	0.0205042	-	-	-	-	-	-	-
SORCS2	SORTILIN-RELATED VPS10 DOMAIN CONTAINING RECEPTOR 2	57537	-1.14664	0.0149217	-	-	-	-	-	-	-
TMEM154	HYPOTHETICAL PROTEIN FLJ32028	201799	-1.146802	0.0251245	-	-	-	-	-	-	-
TMEM123	TRANSMEMBRANE PROTEIN 123	114908	-1.146845	0.0118454	-	-	-	-	-	-	-
GTF2IRD1	GTF2I REPEAT DOMAIN CONTAINING 1	9569	-1.148884	0.0044615	-	-	-	-	-	-	-
HDHD2	HALOACID DEHALOGENASE-LIKE HYDROLASE DOMAIN CONTAINING 2	84064	-1.149771	0.0195798	-	-	-	-	-	-	-
GALNT11	UDP-N-ACETYL-ALPHA-D-GALACTOSAMINE:POLYPEPTIDE N-ACETYLGALACTOSAMINYLTRANSFERASE 11 (GALNAC-T11)	63917	-1.150296	0.049263	-	-	-	-	-	-	-
PALM2	A KINASE (PRKA) ANCHOR PROTEIN 2	11217	-1.150321	0.0152487	-	-	-	-	-	-	-
PALM2	A KINASE (PRKA) ANCHOR PROTEIN 2	445815	-1.150321	0.0152487	-	-	-	-	-	-	-
CCDC23	COILED-COIL DOMAIN CONTAINING 23	374969	-1.151543	0.016278	-	-	-	-	-	-	-
ZSCAN1	ZINC FINGER AND SCAN DOMAIN CONTAINING 1	284312	-1.151852	0.0315525	-	-	-	-	-	-	-
	HYPOTHETICAL PROTEIN MGC3196	79064	-1.152533	0.0186681	-	-	-	-	-	-	-
GRASP	GRP1 (GENERAL RECEPTOR FOR PHOSPHOINOSITIDES 1)-ASSOCIATED SCAFFOLD PROTEIN	160622	-1.153155	0.0177166	-	-	-	-	-	-	-
ADI1	ACIREDUCTONE DIOXYGENASE 1	55256	-1.153555	0.0060089	-	-	-	-	-	-	-
RNF169	RING FINGER PROTEIN 169	254225	-1.153626	0.0326129	-	-	-	-	-	-	-
COMMD2	COMM DOMAIN CONTAINING 2	51122	-1.153767	0.0475755	-	-	-	-	-	-	-
SIGLEC10	SIALIC ACID BINDING IG-LIKE LECTIN 10	89790	-1.15394	0.0231416	-	-	-	-	-	-	-
TUBB2A	TUBULIN, BETA 2A	7280	-1.154355	0.0415636	-	-	-	-	-	-	-
NA	15 KDA SELENOPROTEIN	9403	-1.15511	0.0403664	-	-	-	-	-	-	-
MT1H	METALLOTHIONEIN 1H	4496	-1.155315	0.0405802	-	-	-	-	-	-	-
		727730	-1.155315	0.0405802	-	-	-	-	-	-	-
IMMP1L	IMP1 INNER MITOCHONDRIAL MEMBRANE PEPTIDASE-LIKE (S. CEREVISIAE)	196294	-1.15543	0.0486022	-	-	-	-	-	-	-
CLIC2	CHLORIDE INTRACELLULAR CHANNEL 2	1193	-1.155451	0.0299939	-	-	-	-	-	-	-
C12orf65	HYPOTHETICAL PROTEIN FLJ38663	91574	-1.155524	0.0120764	-	-	-	-	-	-	-
NIPSNAP3A	NIPSNAP HOMOLOG 3A (C. ELEGANS)	25934	-1.156694	0.0491797	-	-	-	-	-	-	-
JAM2	JUNCTIONAL ADHESION MOLECULE 2	58494	-1.157206	0.0141452	-	-	-	-	-	-	-
TMEM106B	TRANSMEMBRANE PROTEIN 106B	54664	-1.157232	0.0452735	-	-	-	-	-	-	-
SPINK2	SERINE PEPTIDASE INHIBITOR, KAZAL TYPE 2 (ACROSIN-TRYPSIN INHIBITOR)	6691	-1.15786	0.0072024	-	-	-	-	-	-	-
SPIN1	SPINDLIN	10927	-1.15829	0.0368201	-	-	-	-	-	-	-
ASGR1	ASIALOGLYCOPROTEIN RECEPTOR 1	432	-1.158318	0.0392295	-	-	-	-	-	-	-
	SIMILAR TO MICROTUBULE ASSOCIATED TESTIS SPECIFIC SERINE/THREONINE PROTEIN KINASE	375449	-1.158333	0.0133773	-	-	-	-	-	-	-
FXYD6	FXYD DOMAIN CONTAINING ION TRANSPORT REGULATOR 6	53826	-1.158538	0.0029666	-	-	-	-	-	-	-
RASSF4	RAS ASSOCIATION (RALGDS/AF-6) DOMAIN FAMILY 4	83937	-1.158768	0.0082368	-	-	-	-	-	-	-
SDAD1	SDA1 DOMAIN CONTAINING 1	55153	-1.158874	0.0061854	-	-	-	-	-	-	-
AIM1	ABSENT IN MELANOMA 1	202	-1.159148	0.0056822	-	-	-	-	-	-	-
		728179	-1.161321	0.0074253	-	-	-	-	-	-	-
CEP170	CENTROSOMAL PROTEIN 170KDA	9859	-1.161453	0.026624	-	-	-	-	-	-	-
MKRN3	MAKORIN, RING FINGER PROTEIN, 3	7681	-1.162159	0.0105943	-	-	-	-	-	-	-
OPA3	OPTIC ATROPHY 3 (AUTOSOMAL RECESSIVE, WITH CHOREA AND SPASTIC PARAPLEGIA)	80207	-1.162525	0.0041532	-	-	-	-	-	-	-
MRPL13	MITOCHONDRIAL RIBOSOMAL PROTEIN L13	28998	-1.162859	0.0329774	-	-	-	-	-	-	-
		AFFX-r2-B	-1.163048	0.0478688	-	-	-	-	-	-	-
BBS10	CHROMOSOME 12 OPEN READING FRAME 58	79738	-1.16366	0.016058	-	-	-	-	-	-	-

Gene Name	Gene Description	Entrez ID	Mean FC	*p*- value	PPRE (Lemay)	PPRE	NFkB	JUN	TP53	SP1	CTNNB1
PYGL	PHOSPHORYLASE, GLYCOGEN; LIVER (HERS DISEASE, GLYCOGEN STORAGE DISEASE TYPE VI)	5836	-1.163769	0.0059577	-	-	-	-	-	-	-
NA	HIGH-MOBILITY GROUP (NONHISTONE CHROMOSOMAL) PROTEIN 4-LIKE	128872	-1.164903	0.0044057	-	-	-	-	-	-	-
PLXND1	PLEXIN D1	23129	-1.165226	0.0039283	-	-	-	-	-	-	-
NUCKS1	NUCLEAR CASEIN KINASE AND CYCLIN-DEPENDENT KINASE SUBSTRATE 1	64710	-1.165875	0.0159653	-	-	-	-	-	-	-
PLEKHB2	PLECKSTRIN HOMOLOGY DOMAIN CONTAINING, FAMILY B (EVECTINS) MEMBER 2	55041	-1.166555	0.0155951	-	-	-	-	-	-	-
SLC39A11	SOLUTE CARRIER FAMILY 39 (METAL ION TRANSPORTER), MEMBER 11	201266	-1.166857	0.0033486	-	-	-	-	-	-	-
SDCCAG10	SEROLOGICALLY DEFINED COLON CANCER ANTIGEN 10	10283	-1.167058	0.0079403	-	-	-	-	-	-	-
PPP2R5E	PROTEIN PHOSPHATASE 2, REGULATORY SUBUNIT B (B56), EPSILON ISOFORM	5529	-1.167289	0.0135507	-	-	-	-	-	-	-
CSDA	COLD SHOCK DOMAIN PROTEIN A	8531	-1.167414	0.0180892	-	-	-	-	-	-	-
		AFFX-r2-E	-1.167818	0.0492289	-	-	-	-	-	-	-
UBE2E2	UBIQUITIN-CONJUGATING ENZYME E2E 2 (UBC4/5 HOMOLOG, YEAST)	7325	-1.168145	0.0115302	-	-	-	-	-	-	-
RPS3A	SIMILAR TO RIBOSOMAL PROTEIN S3A	439992	-1.168365	0.0190551	-	-	-	-	-	-	-
TUBA1A	TUBULIN, ALPHA 3	7846	-1.168632	0.0118547	-	-	-	-	-	-	-
ZBTB1	ZINC FINGER AND BTB DOMAIN CONTAINING 1	22890	-1.16937	0.0427042	-	-	-	-	-	-	-
C14orf126	CHROMOSOME 14 OPEN READING FRAME 126	112487	-1.169411	0.0194996	-	-	-	-	-	-	-
ISLR2	IMMUNOGLOBULIN SUPERFAMILY CONTAINING LEUCINE-RICH REPEAT 2	57611	-1.169499	0.011931	-	-	-	-	-	-	-
APOBEC3A	APOLIPOPROTEIN B MRNA EDITING ENZYME, CATALYTIC POLYPEPTIDE-LIKE 3A	200315	-1.169932	0.0184374	-	-	-	-	-	-	-
C9orf78	HYPOTHETICAL PROTEIN HSPC220	51759	-1.17008	0.0089992	-	-	-	-	-	-	-
MAGEH1	MELANOMA ANTIGEN FAMILY H, 1	28986	-1.170204	0.0118216	-	-	-	-	-	-	-
	SIGNAL-REGULATORY PROTEIN BETA 2	284759	-1.170389	0.0358908	-	-	-	-	-	-	-
COMMD9	COMM DOMAIN CONTAINING 9	29099	-1.170405	0.0017629	-	-	-	-	-	-	-
RENBP	RENIN BINDING PROTEIN	5973	-1.170447	0.0040795	-	-	-	-	-	-	-
		AFFX-LysX	-1.171156	0.0495352	-	-	-	-	-	-	-
GNG10	GUANINE NUCLEOTIDE BINDING PROTEIN (G PROTEIN), GAMMA 10	2790	-1.171204	0.0130373	-	-	-	-	-	-	-
GNG10	GUANINE NUCLEOTIDE BINDING PROTEIN (G PROTEIN), GAMMA 10	552891	-1.171204	0.0130373	-	-	-	-	-	-	-
	HYPOTHETICAL PROTEIN MGC61571	152100	-1.171468	0.0417326	-	-	-	-	-	-	-
	P18SRP PROTEIN	285672	-1.172755	0.0039412	-	-	-	-	-	-	-
		AFFX-PheX	-1.173784	0.0424334	-	-	-	-	-	-	-
TBC1D2	TBC1 DOMAIN FAMILY, MEMBER 2	55357	-1.173801	0.0028476	-	-	-	-	-	-	-
LSM5	LSM5 HOMOLOG, U6 SMALL NUCLEAR RNA ASSOCIATED (S. CEREVISIAE)	23658	-1.173874	0.0331908	-	-	-	-	-	-	-
LHFP	LIPOMA HMGIC FUSION PARTNER	10186	-1.174024	0.0085318	-	-	-	-	-	-	-
UBLCP1	UBIQUITIN-LIKE DOMAIN CONTAINING CTD PHOSPHATASE 1	134510	-1.174147	0.010435	-	-	-	-	-	-	-
NA	SIMILAR TO CYTOCHROME C, SOMATIC	374408	-1.174153	0.0274455	-	-	-	-	-	-	-
PET112L	PET112-LIKE (YEAST)	5188	-1.174535	0.0048535	-	-	-	-	-	-	-
SEC24D	SEC24 RELATED GENE FAMILY, MEMBER D (S. CEREVISIAE)	9871	-1.176188	0.0353673	-	-	-	-	-	-	-
NA	HYPOTHETICAL LOC389362	389362	-1.176509	0.0230799	-	-	-	-	-	-	-
TMEM16K	TRANSMEMBRANE PROTEIN 16K	55129	-1.176606	0.0112776	-	-	-	-	-	-	-
COMMD8	COMM DOMAIN CONTAINING 8	54951	-1.177045	0.0110733	-	-	-	-	-	-	-
BMP2K	BMP2 INDUCIBLE KINASE	55589	-1.17723	0.0114283	-	-	-	-	-	-	-
	HYPOTHETICAL PROTEIN MGC21675	92070	-1.177705	0.027341	-	-	-	-	-	-	-
NA	SIMILAR TO 60S RIBOSOMAL PROTEIN L29 (P23)	391132	-1.177821	0.0166345	-	-	-	-	-	-	-
ASRGL1	ASPARAGINASE LIKE 1	80150	-1.178152	0.0042982	-	-	-	-	-	-	-
C1orf163	CHROMOSOME 1 OPEN READING FRAME 163	65260	-1.178905	0.0014313	-	-	-	-	-	-	-
GAS2L1	GROWTH ARREST-SPECIFIC 2 LIKE 1	10634	-1.179225	0.001178	-	-	-	-	-	-	-
ARHGEF10L	RHO GUANINE NUCLEOTIDE EXCHANGE FACTOR (GEF) 10-LIKE	55160	-1.1806	0.0015057	-	-	-	-	-	-	-
NA	CHROMOSOME 6 OPEN READING FRAME 160	387066	-1.180775	0.0363965	-	-	-	-	-	-	-
C16orf54	CHROMOSOME 16 OPEN READING FRAME 54	283897	-1.180868	0.0292188	-	-	-	-	-	-	-
FPGT	FUCOSE-1-PHOSPHATE GUANYLYLTRANSFERASE	8790	-1.181749	0.0432267	-	-	-	-	-	-	-
ZFP1	ZINC FINGER PROTEIN 1 HOMOLOG (MOUSE)	162239	-1.182129	0.0380297	-	-	-	-	-	-	-
		730249	-1.182606	0.0130136	-	-	-	-	-	-	-
PAQR3	PROGESTIN AND ADIPOQ RECEPTOR FAMILY MEMBER III	152559	-1.182647	0.0295862	-	-	-	-	-	-	-
FAM33B	FAMILY WITH SEQUENCE SIMILARITY 33, MEMBER A	348235	-1.182864	0.0051567	-	-	-	-	-	-	-
ZNF467	ZINC FINGER PROTEIN 467	168544	-1.184361	0.0018014	-	-	-	-	-	-	-
ARRB1	ARRESTIN, BETA 1	408	-1.184493	0.0114396	-	-	-	-	-	-	-
RTN1	RETICULON 1	6252	-1.184851	0.0012822	-	-	-	-	-	-	-
LYRM5	HYPOTHETICAL PROTEIN LOC144363	144363	-1.185792	0.0365637	-	-	-	-	-	-	-
OAZ3	ORNITHINE DECARBOXYLASE ANTIZYME 3	51686	-1.186194	0.0204961	-	-	-	-	-	-	-
CCDC58	COILED-COIL DOMAIN CONTAINING 58	131076	-1.186428	0.0185802	-	-	-	-	-	-	-
CLEC4A	C-TYPE LECTIN DOMAIN FAMILY 4, MEMBER A	50856	-1.187405	0.0091159	-	-	-	-	-	-	-
RWDD1	RWD DOMAIN CONTAINING 1	51389	-1.187515	0.0138635	-	-	-	-	-	-	-
C10orf54	CHROMOSOME 10 OPEN READING FRAME 54	64115	-1.189189	0.0030342	-	-	-	-	-	-	-
MRPL52	MITOCHONDRIAL RIBOSOMAL PROTEIN L52	122704	-1.189854	0.0059971	-	-	-	-	-	-	-
NEFH	NEUROFILAMENT, HEAVY POLYPEPTIDE 200KDA	4744	-1.190091	0.0084483	-	-	-	-	-	-	-
DDX6	DEAD (ASP-GLU-ALA-ASP) BOX POLYPEPTIDE 6	1656	-1.19039	0.0279737	-	-	-	-	-	-	-
DTX4	DELTEX 4 HOMOLOG (DROSOPHILA)	23220	-1.190469	0.0056332	-	-	-	-	-	-	-
CETN3	CENTRIN, EF-HAND PROTEIN, 3 (CDC31 HOMOLOG, YEAST)	1070	-1.191756	0.0124117	-	-	-	-	-	-	-
		AFFX-BioB	-1.19194	0.0477618	-	-	-	-	-	-	-
FAM105A	FAMILY WITH SEQUENCE SIMILARITY 105, MEMBER A	54491	-1.192204	0.0364961	-	-	-	-	-	-	-
MIDN	MIDNOLIN	90007	-1.193745	0.009001	-	-	-	-	-	-	-
LINGO2	LEUCINE RICH REPEAT NEURONAL 6C	158038	-1.194635	0.0277725	-	-	-	-	-	-	-
CCR2	C-C CHEMOKINE RECEPTOR TYPE 2	729230	-1.195063	0.0171392	-	-	-	-	-	-	-
C1orf54	CHROMOSOME 1 OPEN READING FRAME 54	79630	-1.195882	0.0111799	-	-	-	-	-	-	-
C4orf34	HYPOTHETICAL PROTEIN LOC201895	201895	-1.196227	0.0062029	-	-	-	-	-	-	-
SLC25A43	SIMILAR TO SOLUTE CARRIER FAMILY 25 , MEMBER 16	203427	-1.196464	0.0259704	-	-	-	-	-	-	-
MRPL19	MITOCHONDRIAL RIBOSOMAL PROTEIN L19	9801	-1.198924	0.0097521	-	-	-	-	-	-	-
SECTM1	SECRETED AND TRANSMEMBRANE 1	6398	-1.199314	0.0027162	-	-	-	-	-	-	-
GPR137B	G PROTEIN-COUPLED RECEPTOR 137B	7107	-1.200063	0.0043895	-	-	-	-	-	-	-
		AFFX-Dap	-1.200219	0.0367189	-	-	-	-	-	-	-
TIPIN	TIMELESS-INTERACTING PROTEIN	54962	-1.201476	0.0077318	-	-	-	-	-	-	-
RP2	RETINITIS PIGMENTOSA 2 (X-LINKED RECESSIVE)	6102	-1.203056	0.0328341	-	-	-	-	-	-	-
RIN2	RAS AND RAB INTERACTOR 2	54453	-1.203088	0.0073548	-	-	-	-	-	-	-
	TESTIS/PROSTATE/PLACENTA-EXPRESSED PROTEIN, ISOFORM 2	374739	-1.203228	0.0498032	-	-	-	-	-	-	-
FAM26F	HYPOTHETICAL PROTEIN LOC441168	441168	-1.205923	0.0174491	-	-	-	-	-	-	-
	CDC42 GTPASE-ACTIVATING PROTEIN	57514	-1.207647	0.0068501	-	-	-	-	-	-	-
NSL1	CHROMOSOME 1 OPEN READING FRAME 48	25936	-1.207676	0.0092761	-	-	-	-	-	-	-
C5orf20	CHROMOSOME 5 OPEN READING FRAME 20	140947	-1.208094	0.0176661	-	-	-	-	-	-	-
PSTPIP2	PROLINE-SERINE-THREONINE PHOSPHATASE INTERACTING PROTEIN 2	9050	-1.20901	0.0041413	-	-	-	-	-	-	-
		AFFX-r2-B	-1.209511	0.0482592	-	-	-	-	-	-	-
RRAGD	RAS-RELATED GTP BINDING D	58528	-1.210098	0.0017077	-	-	-	-	-	-	-
DDEF2	DEVELOPMENT AND DIFFERENTIATION ENHANCING FACTOR 2	8853	-1.210852	0.016878	-	-	-	-	-	-	-
		AFFX-r2-B	-1.211417	0.0304278	-	-	-	-	-	-	-
C4orf18	CHROMOSOME 4 OPEN READING FRAME 18	51313	-1.21318	0.0453996	-	-	-	-	-	-	-
C17orf75	CHROMOSOME 17 OPEN READING FRAME 75	64149	-1.214491	0.0193072	-	-	-	-	-	-	-
SUCNR1	SUCCINATE RECEPTOR 1	56670	-1.214671	0.0153125	-	-	-	-	-	-	-
		AFFX-r2-B	-1.21475	0.0224684	-	-	-	-	-	-	-
FRMD4B	FERM DOMAIN CONTAINING 4B	23150	-1.216206	0.0204847	-	-	-	-	-	-	-
SLC7A7	SOLUTE CARRIER FAMILY 7 (CATIONIC AMINO ACID TRANSPORTER, Y+ SYSTEM), MEMBER 7	9056	-1.218751	0.0040842	-	-	-	-	-	-	-
SGTB	SMALL GLUTAMINE-RICH TETRATRICOPEPTIDE REPEAT (TPR)-CONTAINING, BETA	54557	-1.219892	0.0074884	-	-	-	-	-	-	-
	HYPOTHETICAL PROTEIN LOC647115	647115	-1.220979	0.0152368	-	-	-	-	-	-	-
CPM	CARBOXYPEPTIDASE M	1368	-1.2214	0.0072475	-	-	-	-	-	-	-
C5orf23	HYPOTHETICAL PROTEIN FLJ14054	79614	-1.222186	0.0012533	-	-	-	-	-	-	-
C1orf115	CHROMOSOME 1 OPEN READING FRAME 115	79762	-1.22622	0.0002815	-	-	-	-	-	-	-
METTL7A	METHYLTRANSFERASE LIKE 7A	25840	-1.226819	0.0011181	-	-	-	-	-	-	-
PIGW	PHOSPHATIDYLINOSITOL GLYCAN, CLASS W	284098	-1.230831	0.0105573	-	-	-	-	-	-	-
BPGM	2,3-BISPHOSPHOGLYCERATE MUTASE	669	-1.232606	0.0119299	-	-	-	-	-	-	-
TMEM176B	LR8 PROTEIN	28959	-1.235899	0.0004675	-	-	-	-	-	-	-
CPVL	CARBOXYPEPTIDASE, VITELLOGENIC-LIKE	54504	-1.237635	0.0022844	-	-	-	-	-	-	-
HIST4H4	H4 HISTONE, FAMILY 2	8364	-1.238709	0.0071903	-	-	-	-	-	-	-
GNPTAB	HYPOTHETICAL PROTEIN DKFZP762B226	79158	-1.239624	0.000794	-	-	-	-	-	-	-
C1orf85	CHROMOSOME 1 OPEN READING FRAME 85	112770	-1.240986	0.0004147	-	-	-	-	-	-	-
SEC22L1	SEC22 VESICLE TRAFFICKING PROTEIN-LIKE 1 (S. CEREVISIAE)	9554	-1.242171	0.0006142	-	-	-	-	-	-	-
NA	HYPOTHETICAL GENE SUPPORTED BY AK098314	400446	-1.251283	0.0067499	-	-	-	-	-	-	-
TMEM55A	TRANSMEMBRANE PROTEIN 55A	55529	-1.253384	0.0131413	-	-	-	-	-	-	-

Gene Name	Gene Description	Entrez ID	Mean FC	*p*- value	PPRE (Lemay)	PPRE	NFkB	JUN	TP53	SP1	CTNNB1
		AFFX-r2-B	-1.260215	0.0114312	-	-	-	-	-	-	-
C20orf160	CHROMOSOME 20 OPEN READING FRAME 160	140706	-1.268924	0.0178641	-	-	-	-	-	-	-
C17orf58	CHROMOSOME 17 OPEN READING FRAME 58	284018	-1.269633	0.001732	-	-	-	-	-	-	-
MARCO	MACROPHAGE RECEPTOR WITH COLLAGENOUS STRUCTURE	8685	-1.270863	0.0004688	-	-	-	-	-	-	-
NA	HYPOTHETICAL PROTEIN LOC283143	283143	-1.272363	0.0135311	-	-	-	-	-	-	-
CH25H	CHOLESTEROL 25-HYDROXYLASE	9023	-1.273423	0.0184788	-	-	-	-	-	-	-
NFE2L3	NUCLEAR FACTOR (ERYTHROID-DERIVED 2)-LIKE 3	9603	-1.274559	0.0010315	-	-	-	-	-	-	-
ZNRF2	ZINC AND RING FINGER 2	223082	-1.275468	0.0011366	-	-	-	-	-	-	-
TMEM176A	HEPATOCELLULAR CARCINOMA-ASSOCIATED ANTIGEN 112	55365	-1.287801	4.95E-05	-	-	-	-	-	-	-
ZNF329	ZINC FINGER PROTEIN 329	79673	-1.288796	0.0008987	-	-	-	-	-	-	-
CAPG	CAPPING PROTEIN (ACTIN FILAMENT), GELSOLIN-LIKE	822	-1.297507	0.0001064	-	-	-	-	-	-	-
C3orf64	HYPOTHETICAL PROTEIN FLJ13078	285203	-1.301481	0.0173524	-	-	-	-	-	-	-
NLN	NEUROLYSIN (METALLOPEPTIDASE M3 FAMILY)	57486	-1.316094	0.0040963	-	-	-	-	-	-	-
PRR16	MESENCHYMAL STEM CELL PROTEIN DSC54	51334	-1.32879	0.0032186	-	-	-	-	-	-	-
LILRA5	LEUKOCYTE IMMUNOGLOBULIN-LIKE RECEPTOR, SUBFAMILY B (WITH TM AND ITIM DOMAINS), MEMBER 7	353514	-1.337664	0.0149745	-	-	-	-	-	-	-
		728598	-1.341195	0.0011096	-	-	-	-	-	-	-
LILRA3	LEUKOCYTE IMMUNOGLOBULIN-LIKE RECEPTOR, SUBFAMILY A (WITHOUT TM DOMAIN), MEMBER 3	11026	-1.358668	0.0007127	-	-	-	-	-	-	-
SRGAP2P1	SIMILAR TO SLIT-ROBO RHO GTPASE-ACTIVATING PROTEIN 2 (SRGAP2) (FORMIN-BINDING PROTEIN 2)	653464	-1.366441	0.0089241	-	-	-	-	-	-	-
ADAMDEC1	ADAM-LIKE, DECYSIN 1	27299	-1.370034	0.0002758	-	-	-	-	-	-	-
		AFFX-HUM	-1.376275	0.0135264	-	-	-	-	-	-	-
NA	HYPOTHETICAL LOC440731	440731	-1.378109	0.003014	-	-	-	-	-	-	-
CD302	CD302 ANTIGEN	9936	-1.380236	0.0005615	-	-	-	-	-	-	-
EMR1	EGF-LIKE MODULE CONTAINING, MUCIN-LIKE, HORMONE RECEPTOR-LIKE 1	2015	-1.381868	1.30E-05	-	-	-	-	-	-	-
OLFML2B	OLFACTOMEDIN-LIKE 2B	25903	-1.423198	0.0022713	-	-	-	-	-	-	-
ARNT2	ARYL-HYDROCARBON RECEPTOR NUCLEAR TRANSLOCATOR 2	9915	-1.438528	0.0021102	-	-	-	-	-	-	-
		AFFX-HUM	-1.455044	0.023053	-	-	-	-	-	-	-
		AFFX-HUM	-1.506677	0.0379754	-	-	-	-	-	-	-
		728177	-1.654891	0.0132532	-	-	-	-	-	-	-
